# Palladium-catalyzed picolinamide-directed iodination of remote *ortho*-C−H bonds of arenes: Synthesis of tetrahydroquinolines

**DOI:** 10.3762/bjoc.12.119

**Published:** 2016-06-17

**Authors:** William A Nack, Xinmou Wang, Bo Wang, Gang He, Gong Chen

**Affiliations:** 1 Department of Chemistry, The Pennsylvania State University, University Park, Pennsylvania 16802, United States; 2 State Key Laboratory and Institute of Elemento-Organic Chemistry, Nankai University, Tianjin 300071, China; 3 Collaborative Innovation Center of Chemical Science and Engineering (Tianjin), Tianjin 300071, China

**Keywords:** iodination, palladium, remote C–H activation, tetrahydroquinoline

## Abstract

A new palladium-catalyzed picolinamide (PA)-directed *ortho*-iodination reaction of ε-C(sp^2^)−H bonds of γ-arylpropylamine substrates is reported. This reaction proceeds selectively with a variety of γ-arylpropylamines bearing strongly electron-donating or withdrawing substituents, complementing our previously reported PA-directed electrophilic aromatic substitution approach to this transformation. As demonstrated herein, a three step sequence of Pd-catalyzed γ-C(sp^3^)−H arylation, Pd-catalyzed ε-C(sp^2^)−H iodination, and Cu-catalyzed C−N cyclization enables a streamlined synthesis of tetrahydroquinolines bearing diverse substitution patterns.

## Introduction

**Scheme 1:**
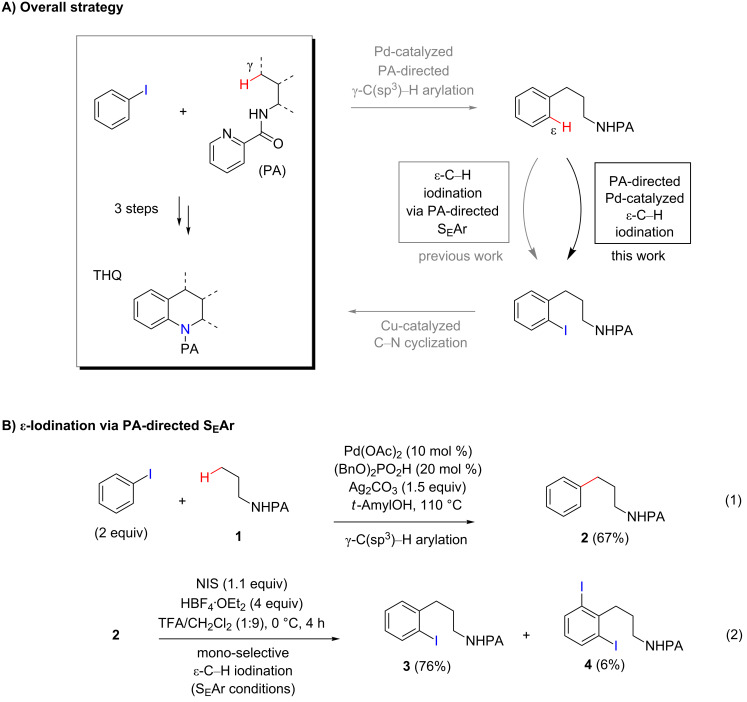
New synthetic strategy for THQs via PA-directed C−H functionalization.

Tetrahydroquinoline (THQ) is an important *N*-heterocyclic scaffold found in many natural products and pharmaceutical agents [1–2]. Efficient and generally applicable methods for the synthesis of THQs with complex substitution patterns are still in great demand [3–7]. Recently, we reported a synthetic strategy for THQs based on picolinamide (PA)-directed sequential C−H functionalization reactions starting from readily accessible aryl iodide and alkylamine precursors (Scheme 1) [8]. Alkylpicolinamides were first subjected to Pd-catalyzed γ-C(sp^3^)−H arylation with aryl iodides to form γ-arylpropylpicolinamides [9–20]. These γ-arylpropylpicolinamides were then selectively iodinated at the remote ε-C(sp^2^)−H position via a rarely precedented PA-directed electrophilic aromatic substitution (S_E_Ar) reaction (Scheme 1, reaction 2) [21–22]. Copper-catalyzed intramolecular C−N cyclization of these *ortho-*iodinated intermediates provided PA-coupled THQ products in good yields.

Although ε-C−H iodination via directed S_E_Ar proceeds with excellent yield and mono-selectivity for many γ-arylpropylpicolinamides, the scope of these PA-directed S_E_Ar reactions is limited to arenes bearing moderate electron-donating or withdrawing groups. Arene substrates bearing strongly electron-donating substituents typically gave substantial amounts of undesired iodinated side products via competing innate S_E_Ar processes, and arene substrates bearing strongly electron-withdrawing substituents were often unreactive. Herein, we report our development of a Pd-catalyzed PA-directed iodination reaction of ε-C(sp^2^)−H bonds of γ-arylpropylpicolinamides. This Pd-catalyzed reaction is complementary in scope to the directed S_E_Ar iodination approach and allows for the efficient synthesis of a broad range of THQs with diverse substitution patterns.

## Results and Discussion

Methods for metal-catalyzed halogenation of *ortho* C−H bonds at the more remote ε position are scarce, in contrast to the large number of *ortho* C−H halogenation reactions of arenes effected by more proximal directing groups [23–33]. Fundamentally, it is challenging to achieve efficient reactions through kinetically unfavorable seven-membered palladacycle intermediates. Furthermore, the electrophilic reagents used for C–H halogenation can often react with arenes through undirected S_E_Ar pathways, which need to be suppressed for regioselectivity. To address this issue upfront, we commenced our study of Pd-catalyzed ε-C−H halogenation with 3-arylpropylpicolinamide **5** bearing a strongly electron-donating OMe group (Table 1, see Supporting Information File 1 for the preparation of **5**). Iodination of **5** under our previous S_E_Ar protocol gave undirected iodination product **7** as the major product; only a trace amount of *ortho*-iodination product **6** was detected (Table 1, entries 1 and 2). Iodination of **5** under a variety of Pd-catalyzed oxidative conditions gave either low conversion or poor regioselectivity (Table 1, entries 3–5). To our delight, the use of a combination of 2 equiv of I_2_ and 2 equiv of PhI(OAc)_2_ in DMF at 110 °C gave the desired product **6** in good yield and moderate selectivity. Similar conditions were reported by Yu to effect the Pd-catalyzed NHTf-directed iodination of δ-C(sp^2^)−H bonds of β-phenylethyl triflamides [33]. IOAc generated in situ is believed to be the active iodinating species. DMF was found to be the best solvent for this reaction (Table 1, entry 9 vs 11 and 12). Moreover, we found that the choice of alkali carbonate base was important: replacing K_2_CO_3_ with KHCO_3_ or Na_2_CO_3_ gave notably improved yields and ortho selectivity (Table 1, entries 9 and 10) [34–35]. By analogy with similar Pd-catalyzed directed C–H halogenation reactions, we speculate that the catalytic cycle follows a sequence of C−H palladation, oxidative addition and reductive elimination [36–37].

**Table 1:** Optimization of Pd-catalyzed *ortho* C−H iodination of **5**.^a^

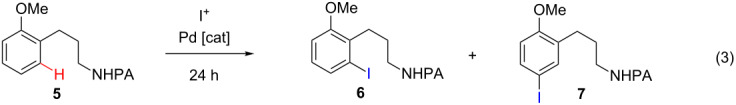					

entry	reagents (equiv)	solvent	temperature (°C)	yield (%)^b^	
				**6**	**7**

1	NIS (1.5), HBF_4_·EtO_2_ (4.0)	T/D^c^	0	<2	68
2	NIS (1.5)	T/D	0	<2	82
3	Pd(OAc)_2_ (10 mol %), NIS (1.5)	chlorobenzene	110	<2	74
4	Pd(OAc)_2_ (10 mol %), NaI (1.5), NaIO_3_ (1.5), K_2_S_2_O_8_ (2.0)	*n*-BuOH	110	<2	<2
5	Pd(OAc)_2_ (10 mol %), I_2_ (2.0), K_2_S_2_O_8_ (2)	DMF	110	<2	60
6	Pd(OAc)_2_ (10 mol %), I_2_ (2.0), PhI(OAc)_2_ (2.0)	DMF	110	43	25
7	Pd(OAc)_2_ (10 mol %), I_2_ (2.0), PhI(OAc)_2_ (2.0), K_2_CO_3_ (1.0)	DMF	110	14	11
8	Pd(OAc)_2_ (10 mol %), I_2_ (2.0), PhI(OAc)_2_ (2.0), KHCO_3_ (2.0)	DMF	110	45	12
9	Pd(OAc)_2_ (10 mol %), I_2_ (2.0), PhI(OAc)_2_ (2.0), KHCO_3_ (1.0)	DMF	110	75 (72)^d^	9 (5)^d^
10	Pd(OAc)_2_ (10 mol %), I_2_ (2.0), PhI(OAc)_2_ (2.0), Na_2_CO_3_ (1.0)	DMF	110	80	8
11	Pd(OAc)_2_ (10 mol %), I_2_ (2.0), PhI(OAc)_2_ (2.0), KHCO_3_ (1.0)	dichloroethane	110	16	58
12	Pd(OAc)_2_ (10 mol %), I_2_ (2.0), PhI(OAc)_2_ (2.0), KHCO_3_ (1.0)	dioxane	110	13	65
13	I_2_ (2.0), PhI(OAc)_2_ (2.0), KHCO_3_ (1.0)	DMF	110	<2	64

^a^All screening reactions were carried out in a 10 mL glass vial on a 0.2 mmol scale: ^b^Yields are based on ^1^H NMR analysis of the reaction mixture using CH_2_Br_2_ as internal standard; ^c^T/D: TFA (T)/CH_2_Cl_2_ (D); ^d^isolated yield.

With the best conditions in hand (Table 1, entries 9 and 10), we then examined the substrate scope of this Pd-catalyzed iodination of γ-arylpropylpicolinamides (Table 2). The γ-arylpropylpicolinamides were prepared from the corresponding *N*-alkylpicolinamides and aryl iodides under our (BnO)_2_PO_2_H-promoted Pd-catalyzed γ-C(sp^3^)−H arylation conditions (see Supporting Information File 1 for details). The substrate scope was chosen to complement the S_E_Ar method, which is notably incompatible with NO_2_, F and OMe substituents. In contrast to the mono-selectivity of the directed S_E_Ar approach (reaction 2, Scheme 1), iodination of γ-phenylpropylpicolinamide **2** bearing two equivalent *ortho* C−H bonds under Pd-catalyzed conditions A gave a mixture of mono-iodinated **3** and *ortho* diiodinated product **4**. However, no *para*-iodinated side product was formed. With 4 equiv of PhI(OAc)_2_/I_2_ and 1 equiv of KHCO_3_, **4** can be formed as the major product in 69% yield.

**Table 2:** Substrate scope of Pd-catalyzed ε-C−H iodination and Cu-catalyzed C−N cyclization to form THQs^a^.

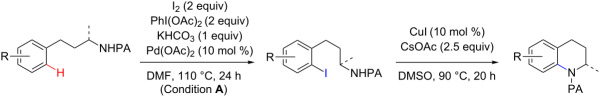			

C–H arylation^b^	iodination		C–N cyclization

	Pd catalyzed	directed S_E_Ar	

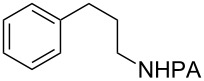 **2** (67%)	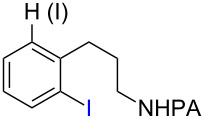 **3** (mono-I, 47%) + **4** (di-I, 25%)^c^	**3** (76%) + **4** (6%)	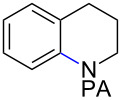 **8** (93%)
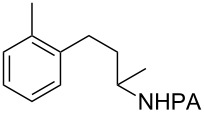 **9** (81%)	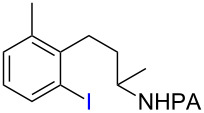 **10** (75%)	**10** (60%) (*o*/*x* = 5:3)^c^	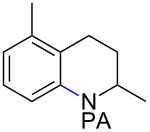 **11** (96%)
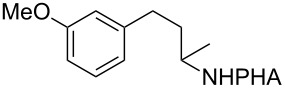 **12** (81%)	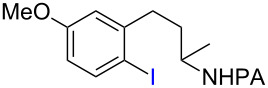 **13** (68%)	**13** (50%) (*o*/*x* = 5:4)^c^	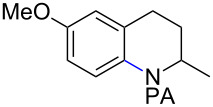 **14** (94%)
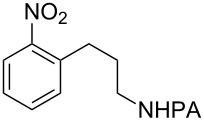 **15** (28%)	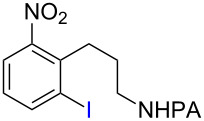 **16** (68%)	NR	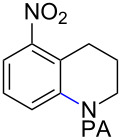 **17** (47%)
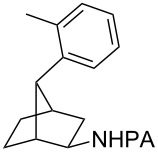 **18** (60%)	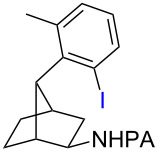 **19** (56%)	**19** (20%) (*o*/*x* = 1:4)^c^	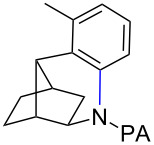 **20** (85%)
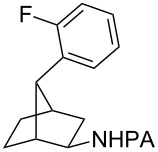 **21** (95%)	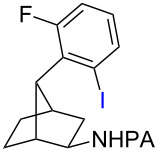 **22** (53% or 85%^d^)	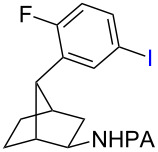 **23** (90%) X-ray	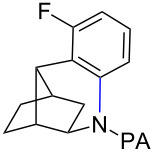 **24** (78%)

^a^Yields are based on isolated product on a 0.2 mmol scale; ^b^see reaction 1 in Scheme 1B for conditions for Pd-catalyzed C−H arylation; ^c^di: *ortho*-diiodinated isomer, *x*: mixture of other iodinated isomers; ^d^conditions B: I_2_ (2 equiv), PhI(OAc)_2_ (2 equiv), Pd(OAc)_2_ (10 mol %), Na_2_CO_3_ (1 equiv), DMF, 110 °C, 24 h.

**Scheme 2:**
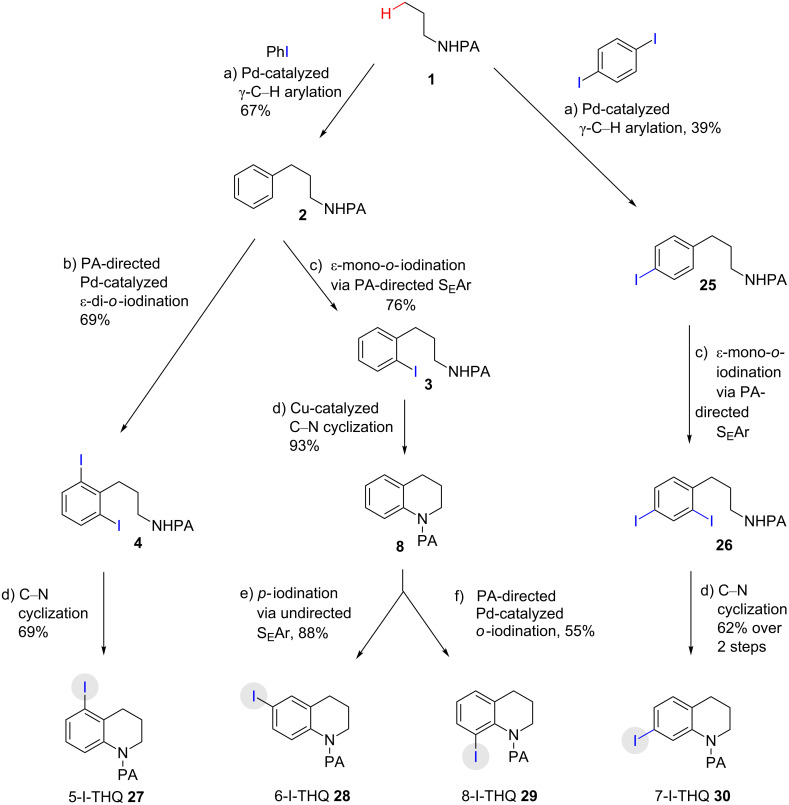
Preparation of iodo-substituted THQs via PA-directed C−H functionalization strategy. a) ArI (2 equiv), Pd(OAc)_2_ (10 mol %), (BnO)_2_PO_2_H (20 mol %), Ag_2_CO_3_ (1.5 equiv), *t*-AmylOH, 110 °C, 24h; b) Pd(OAc)_2_ (10 mol %), I_2_ (4 equiv), PhI(OAc)_2_ (4 equiv), KHCO_3_ (1 equiv), 130 °C, DMF, 24 h; c) NIS (1.1 equiv), HBF_4_**^.^**OEt_2_ (4), TFA/DCM (1:9), 2.5 mM, 0 °C, 4 h; d) CuI (10 mol %), CsOAc (2.5 equiv), DMSO, Ar, 90 °C, 20 h; e) NIS (1.1 equiv), TFA/DCM (1:9), 2.5 mM, rt, 16 h; f) Pd(OAc)_2_ (15 mol %), NIS (2.5 equiv), α,α,α-trifluorotoluene, Ar, 100 °C, 24 h.

Arenes bearing *meta*-substituents (e.g., **12**) were selectively iodinated at the less hindered *ortho* position. Pd-catalyzed iodination of substrate **15** bearing a strongly electron-withdrawing NO_2_ group also proceeded smoothly to give **16**; this substrate is unreactive to directed S_E_Ar. The rigid arylnorbornane scaffold **18** is incompatible with directed S_E_Ar, but was iodinated selectively at the ortho position under Pd-catalyzed conditions without the formation of regioisomeric side products. The strong *para*-directing effect exerted by aryl fluoride substituents overrides directed S_E_Ar selectivity [38–39]. Thus, we observed only *para*-iodinated compound **23** when **21** was subjected to the directed S_E_Ar protocol. In contrast, using our Pd-catalyzed iodination (conditions B), *ortho-*iodinated product **22** was obtained via Pd-catalyzed iodination as the only product in excellent yield. The iodinated intermediates could be readily cyclized under our previously reported Cu-catalyzed conditions to give PA-coupled THQ products with various substitution patterns in good yields (Scheme 2) [8].

**Scheme 3:**
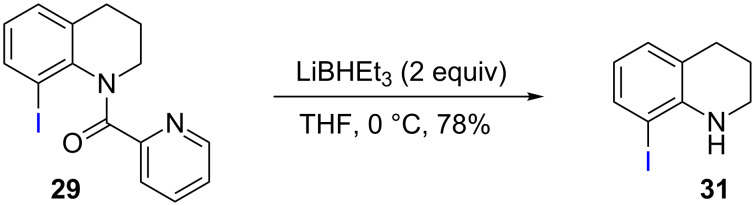
Removal of PA auxiliary from THQ product.

As shown in Scheme 2, Pd-catalyzed PA-directed ε-C−H iodination can be used in concert with PA-directed γ-C−H arylation, PA-directed S_E_Ar iodination, and undirected S_E_Ar iodination to quickly access THQs **27**–**30** bearing iodo groups at different positions on the arene ring [40–42]. *Ortho*-diiodinated product **4** was obtained from **2** in 69% yield using optimized Pd-catalyzed ε-C–H iodination conditions, and Cu-catalyzed C–N cyclization of **4** gave 5-iodo-THQ **27**. PA-THQ **8** was susceptible to iodination at two positions. Under undirected S_E_Ar conditions, 6-iodo-THQ **28** was produced in excellent yield and regioselectivity. Alternatively, a Pd-catalyzed C–H iodination reaction of **8** was developed which provides 8-iodo-THQ **29**. Pd-catalyzed C−H arylation of **1** with *para*-diiodobenzene under the standard arylation conditions gave **25** in moderate yield. Iodination of **25** via PA-directed S_E_Ar gave diiodinated compound **26**, which was cyclized under Cu catalysis to give 7-iodo-THQ **30** in good yield. The PA group of 8-iodo-THQ **29** was readily removed with LiBHEt_3_ to give **31** (Scheme 3) [10].

## Conclusion

In summary, we have developed a new palladium-catalyzed picolinamide (PA)-directed iodination reaction of ε-C(sp^2^)−H bonds of γ-arylpropylamine substrates. This method works well for arenes with a broad range of substituents and offers a complementary scope to our previously reported PA-directed S_E_Ar approach. This Pd-catalyzed PA-directed ε-C−H iodination can be used in concert with the PA-directed γ-C−H arylation, PA-directed S_E_Ar iodination, undirected S_E_Ar iodination, and Cu-catalyzed C−N cyclization to quickly access tetrahydroquinolines bearing diverse substitution patterns from readily accessible starting materials.

## Supporting Information

File 1 Detailed synthetic procedures and characterizations of all new compounds.
