# Supplementary material for: Palladium-catalyzed picolinamide-directed iodination of remote ortho-C−H bonds of arenes: Synthesis of tetrahydroquinolines
Source: Beilstein J Org Chem. 2016 Jun 17;12:1243–9. doi: 10.3762/bjoc.12.119 (PMC4979757; doi:10.3762/bjoc.12.119)
Supplement: File 1 — Detailed synthetic procedures and characterizations of all new compounds. [file Beilstein_J_Org_Chem-12-1243-s001.pdf]

Supporting Information

for

Palladium-catalyzed picolinamide-directed iodination of remote *ortho*-C−H bonds of arenes: Synthesis of tetrahydroquinolines

William A. Nack[1], Xinmou Wang[2], Bo Wang[3], Gang He[*,1] and Gong Chen[*,1,2,3]

Address: [1]Department of Chemistry, The Pennsylvania State University, University Park, Pennsylvania 16802, United States, [2]State Key Laboratory and Institute of Elemento-Organic Chemistry, Nankai University, Tianjin 300071, China and [3]Collaborative Innovation Center of Chemical Science and Engineering (Tianjin), Tianjin 300071, China

Email: Gong Chen - gongchen@nankai.edu.cn; Gang He - hegang@nankai.eud.cn

*Corresponding author

Detailed synthetic procedures and characterizations of all new compounds

1. **Reagents, S2**
2. **Instruments, S2**
3. **Preparation of picolinamide substrates, S2**
4. **General procedure for the preparation of γ-arylpropylamine substrates via γ C–H arylation, S3**
5. **Reaction optimization in Table 1, S6**
6. **Preparation of iodinated compounds via Pd-catalyzed ε C–H iodination, S7**
7. **Preparation of iodinated compounds via directed electrophilic aromatic iodination, S11**
8. **Preparation of tetrahydroquinolines via Cu-catalyzed cyclization, S12**
9. **Preparation of iodinated THQs, S15**
10. **Removal of PA group, S19**
11. **Crystallographic data of 23, S20**
12. **References, S20**
13. **Spectra, S21**

1. Reagents: All commercial materials were used as received unless otherwise noted. Anhydrous solvents were obtained from a JC Meyer solvent dispensing system and used without further purification. Flash chromatography was performed using 230–400 mesh SiliaFlash 60® silica gel (Silicycle Inc.). The following reagents were used as received: $Pd(OAc)_2$ (98%, Aldrich), silver carbonate (99%, Aldrich), iodine (Alfa, 99.8%), iodosobenzene diacetate (Alfa, 98%), dibenzylphosphate (Acros, 98%), copper iodide (Aldrich, 99.999%), cesium acetate (Acros, 99%), *N*-iodosuccinimide (Matrix Scientific, 97%), $HBF_4{\bullet}Et_2O$ (Aldrich), trifluoroacetic acid (Alfa, 99.5%), and α,α,α-trifluorotoluene (Aldrich, 99%).

2. Instruments: NMR spectra were recorded on a Bruker AV-3-HD-500 instrument and calibrated using residual solvent peaks as internal reference. Multiplicities are recorded as: s = singlet, d = doublet, t = triplet, dd = doublet of doublets, td = triplet of doublets, br s = broad singlet, m = multiplet. High resolution ESI mass experiments were operated on a Waters LCT Premier instrument.

3. Preparation of picolinamide substrates

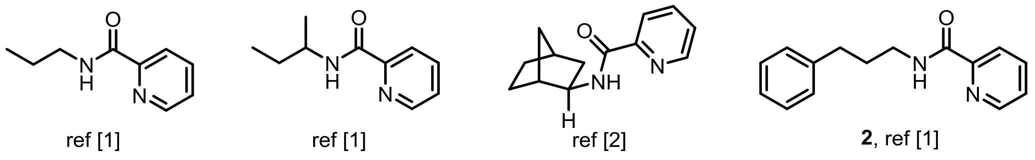

Scheme S1. List of all substrates used in this study.

All known compounds were prepared following the reported procedure and spectral data are consistent with those reported in the literature.[1,2]

4. General procedure for the preparation of γ-arylpropylamine substrates via γ C–H arylation.

Conditions A for substrates 9, 12, 18, 21: A mixture of picolinamide substrate (1.0 equiv), $Pd(OAc)_2$ (0.1 equiv), Ag_2CO_3 (1.5 equiv), aryl iodide (2.0 equiv), and $(BnO)_2PO_2H$ (0.2 equiv) in *t*-AmylOH (0.2M) in a 4 mL glass vial (purged with Ar, sealed with PTFE cap) was heated at 110 ^oC for 24 hours. The reaction mixture was cooled to rt, and filtered through a pad of celite. The filtrate was concentrated in vacuo and the resulting residue was purified by silica gel flash chromatography to give the arylated product.

Conditions B for substrates 5, 15: A mixture of picolinamide substrate (1.0 equiv), $Pd(OAc)_2$ (0.1 equiv), AgOAc (1.5 equiv), and aryl iodide (5.0 equiv) in a 10 mL glass vial (purged with Ar, sealed with PTFE cap) was heated at 150 ^oC for 12 hours.[3] The reaction mixture was cooled to rt, before ethyl acetate was added, and then the mixture was filtered through a pad of celite. The filtrate was concentrated in vacuo and the resulting residue was purified by silica gel flash chromatography to give the arylated product.

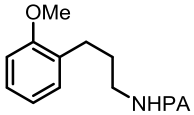

5

Compound **5** was isolated in 36% yield as a colorless oil. ^1H NMR (400 MHz, $CDCl_3$) δ 8.56 (d, *J* = 4.5 Hz, 1H), 8.28 (s, 1H), 8.22 (d, *J* = 5.7 Hz, 1H), 7.85 (td, *J* = 7.7, 1.6 Hz, 1H), 7.44-7.39 (m, 1H), 7.21-7.15 (m, 1H), 6.91-6.84 (m, 1H), 3.85 (s, 3H), 3.49 (q, *J* = 6.7 Hz, 2H), 2.74 (t, *J* =

7.2 Hz, 2H), 1.96-1.89 (m, 2H); ^{13}C NMR (126 MHz, $CDCl_3$) δ 163.87, 157.06, 149.81, 147.67, 136.90, 129.62, 129.32, 126.92, 125.65, 121.73, 120.21, 109.89, 54.83, 38.38, 29.52, 26.94.

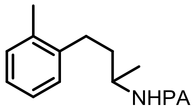

9

Compound **9** was prepared in 81% yield as a colorless oil using arylation conditions **A**. ^{1}H NMR (500 MHz, $CDCl_3$) δ 8.55 (d, *J* = 4.1 Hz, 1H), 8.23 (d, *J* = 7.8 Hz, 1H), 7.98 (d, *J* = 7.9 Hz, 1H), 7.85 (td, *J* = 7.7, 1.6 Hz, 1H), 7.42 (m, 1H), 7.19-7.05 (m, 4H), 4.35-4.24 (m, 1H), 2.77-2.62 (m, 2H), 2.30 (s, 3H), 1.92-1.79 (m, 2H), 1.34 (d, *J* = 6.6 Hz, 3H); ^{13}C NMR (126 MHz, $CDCl_3$) δ 163.71, 150.14, 148.03, 140.07, 137.49, 135.86, 130.28, 128.79, 126.16, 126.11, 126.08, 122.33, 45.52, 37.69, 30.01, 21.18, 19.35; HRMS: calculated for $C_{17}H_{20}N_2O$ [M+H$^+$]: 269.1649, found: 269.1652.

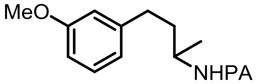

12

Compound **12** was prepared in 81% yield as a colorless oil using arylation conditions **A**. ^{1}H NMR (500 MHz, $CDCl_3$) δ 8.52 (d, *J* = 4.2 Hz, 1H), 8.20 (d, *J* = 7.8 Hz, 1H), 7.93 (d, *J* = 8.3 Hz, 1H), 7.82 (td, *J* = 7.7, 1.6 Hz, 1H), 7.39 (ddd, *J* = 7.4, 4.8, 0.9 Hz, 1H), 7.16 (t, *J* = 7.8 Hz, 1H), 6.78 (d, *J* = 7.5 Hz, 1H), 6.74 (s, 1H), 6.69 (dd, *J* = 8.2, 2.0 Hz, 1H), 4.33-4.15 (m, 1H), 3.76 (s, 3H), 2.68 (t, *J* = 8.0 Hz, 2H), 1.99-1.79 (m, 2H), 1.29 (d, *J* = 6.6 Hz, 3H); ^{13}C NMR (126 MHz, $CDCl_3$) δ 163.61, 159.67, 150.06, 147.96, 143.43, 137.39, 129.36, 126.09, 122.23, 120.76, 114.05, 111.27, 55.12, 45.16, 38.66, 32.65, 21.15; HRMS: calculated for $C_{17}H_{20}N_2O_2$ [M+H$^+$]: 285.1598, found: 285.1604.

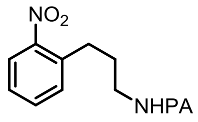

15

Compound **15** was prepared in 28% yield as a colorless oil using arylation conditions **B**. ^{1}H NMR (500 MHz, $CDCl_3$) δ 8.52 (d, *J* = 4.2 Hz, 1H), 8.18 (s, 1H), 8.17 (d, *J* = 7.8 Hz, 1H), 7.88 (dd, *J* = 8.1, 0.8 Hz, 1H), 7.82 (td, *J* = 7.7, 1.6 Hz, 1H), 7.49 (td, *J* = 7.6, 1.1 Hz, 1H), 7.44-7.38 (m, 1H), 7.37 (d, *J* = 7.7 Hz, 1H), 7.34-7.28 (m, 1H), 3.54 (m, 2H), 3.03-2.92 (m, 2H), 2.00 (m, 2H); ^{13}C NMR (126 MHz, $CDCl_3$) δ 164.48, 149.87, 149.28, 148.10, 137.45, 136.63, 133.15, 132.09, 127.27, 126.22, 124.88, 122.23, 39.10, 30.62, 30.61; HRMS: calculated for $C_{15}H_{15}N_3O_3$ [M+H$^+$]: 286.1186, found: 286.1188.

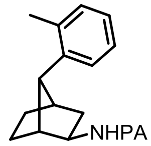

18

Compound **18** was prepared in 60% yield white solid using arylation conditions **A**. ^{1}H NMR (400 MHz, $CDCl_3$) δ 8.13 (d, *J* = 4.2 Hz, 1H), 7.99 (d, *J* = 7.8 Hz, 1H), 7.67 (t, *J* = 7.4 Hz, 1H), 7.36-7.30 (m, 2H), 7.27-7.13 (m, 3H), 7.09-7.07 (m, 1H), 4.14 (td, *J* = 9.0, 3.2 Hz, 1H), 2.98 (s, 1H), 2.80-2.78 (m, 2H), 2.30 (s, 3H), 2.10-1.98 (m, 2H), 1.93-1.85 (m, 1H), 1.76-1.70 (m, 1H), 1.52-1.46 (m, 1H), 1.37-1.31 (m, 1H); ^{13}C NMR (101 MHz, $CDCl_3$) δ 162.74, 149.78, 147.47, 138.53, 137.47, 136.84, 131.01, 127.41, 126.25, 126.17, 125.52, 121.52, 52.85, 52.23, 46.33, 38.56, 38.31, 28.91, 27.68, 20.74; HRMS Calcd for $C_{20}H_{22}N_2O$ [M+H$^+$]: 307.1805; Found: 307.1809.

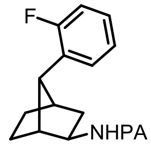

21

Compound **21** was prpared in 94% yield as a white solid using arylation conditions **A**. ^{1}H NMR (400 MHz, $CDCl_3$) δ 8.16 (dd, *J* = 4.7, 0.6 Hz, 1H), 8.03 (d, *J* = 7.8 Hz, 1H), 7.72 (td, *J* = 7.8, 1.5 Hz, 1H), 7.40-7.25 (m, 3H), 7.24-7.19 (m, 1H), 7.12 (t, *J* = 7.5 Hz, 1H), 6.99-6.89 (m, 1H), 4.11 (td, *J* = 8.8, 3.8 Hz, 1H), 3.00 (s, 1H), 2.92 (s, 1H), 2.84 (t, *J* = 3.8 Hz, 1H), 2.04-1.99 (m, 1H), 1.94–1.80 (m, 2H), 1.78-1.70 (m, 1H), 1.54-1.45 (m, 1H), 1.40-1.29 (m, 1H); ^{13}C NMR (101 MHz, $CDCl_3$) δ 163.13, 162.90, 160.68, 149.90, 147.52, 137.06, 129.62, 129.57, 127.97, 127.88, 127.71, 127.56, 125.71, 124.42, 124.39, 121.79, 116.08, 115.85, 52.86, 48.51, 46.66, 46.63, 38.31, 37.88, 28.50, 27.86; HRMS Calcd for $C_{19}H_{19}FN_2O$ [M+H$^+$]: 311.1554, Found: 311.1556.

5. Reaction optimization in Table 1

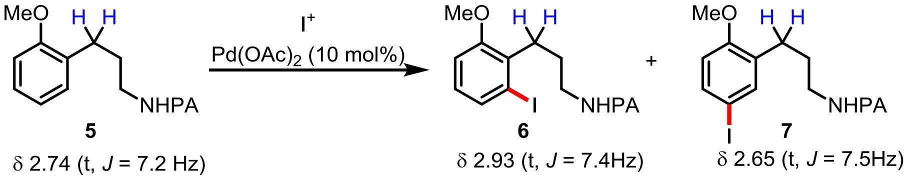

Scheme S2

Reactions were carried out in a 10 mL sealed vial at a 0.2 mmol scale according to the condition in **Table 1**. After 24 hours, the reaction mixtures were cooled to room temperature and water was added. The mixture was extracted with ethyl acetate for three times. The combined organic layer was washed with water and brine, dried over Na_2SO_4, filtered and concentrated in vacuo. The resulting residue was dissolved in 1 mL of deuterated chloroform for ^{1}H NMR analysis. Dibromomethane (34.8 mg, 0.2 mmol, 1 equiv, a singlet peak around 4.95 ppm was set as 1.00) was added as internal standard. Yields of compounds **6**, **7** were determined based on the following method:

Yield of **6** = integration of peak (δ 2.93, triplet) × 100%

Yield of **7** = integration of peak (δ 2.65, triplet) × 100%

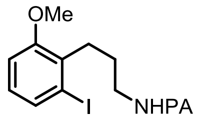

6

^{1}H NMR (400 MHz, $CDCl_3$) δ 8.56 (d, *J* = 4.1 Hz, 1H), 8.35 (s, 1H), 8.21 (d, *J* = 7.8 Hz, 1H), 7.84 (td, *J* = 7.7, 1.6 Hz, 1H), 7.43-7.41 (m, 2H), 6.89-6.81 (m, 2H), 3.84 (s, 3H), 3.49 (q, *J* = 6.6 Hz, 2H), 2.93 (t, *J* =7.4 Hz, 2H), 1.90-1.83 (m, 2H); ^{13}C NMR (101 MHz, CDCl3) δ 164.33, 157.53, 152.20, 150.35, 148.13, 147.68, 137.42, 132.97, 131.73, 128.68, 126.10, 122.32, 110.42, 102.02, 100.08, 55.86, 38.71, 31.91, 28.54; HRMS Calcd for $C_{16}H_{17}IN_2O_2$ [M+H$^+$]: 397.0407, Found: 397.0410.

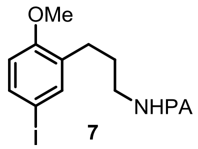

7

^{1}H NMR (400 MHz, $CDCl_3$) δ 8.54 (d, *J* = 4.5 Hz, 1H), 8.22 (s, 1H), 8.18 (d, *J* = 7.8 Hz, 1H) 7.84-7.81 (m, 1H), 7.44-7.39 (m, 3H), 6.59 (d, *J* = 8.2 Hz, 1H), 3.80 (s, 3H), 3.45 (m, 2H), 2.65 (t, *J* = 7.5 Hz, 2H), 1.92-1.85 (m, 2H); ^{13}C NMR (101 MHz, $CDCl_3$) δ 164.28, 157.44, 150.12, 148.07, 138.44, 137.38, 136.01, 132.76, 126.10, 122.22, 112.63, 82.96, 55.48, 38.79, 29.73, 27.14; HRMS Calcd for $C_{16}H_{17}IN_2O_2$ [M+H$^+$]: 397.0407, Found: 397.0411.

6. Preparation of iodinated compounds via Pd-catalyzed ε C–H iodination

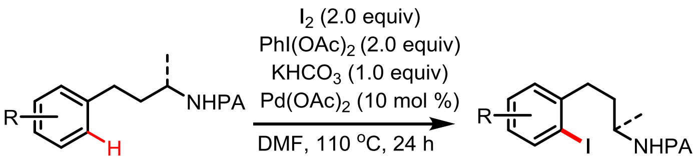

Scheme S3

A mixture of picolinamide substrate (0.2 mol, 1.0 equiv), $Pd(OAc)_2$ (4.5 mg, 0.02 mol, 0.1 equiv), I_2 (101.6 mg, 0.4 mol, 2.0 equiv), $PhI(OAc)_2$ (129 mg, 0.4 mol, 2.0 equiv), and $KHCO_3$

(20 mg, 0.2 mol, 1.0 equiv) in DMF 4 mL in a 10 mL glass vial (purged with Ar, sealed with PTFE cap) was heated at 110 °C for 24 hours. Then the reactions mixture was cooled to room temperature and water was added. The mixture was extracted with ethyl acetate for three times. The combined organic layer was washed with water and brine, dried over Na_2SO_4, filtered and concentrated in vacuo. The resulting residue was purified by silica gel flash chromatography to give the iodinated product.

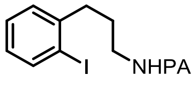

3

Compound **3** was isolated in 47% yield as a colorless oil. Compound **3** has been previously reported and its spectra are consistent with those from the literature.[1]

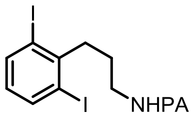

4

Compound **4** was isolated in 25% yield as a white solid. ^{1}H NMR (500 MHz, $CDCl_3$) δ 8.54 (d, *J* = 3.3 Hz, 1H), 8.25 (s, 1H), 8.22 (d, *J* = 7.7 Hz, 1H), 7.85 (t, *J* = 7.4 Hz, 1H), 7.79 (d, *J* = 7.8 Hz, 2H), 7.47-7.37 (m, 1H), 6.49 (t, *J* = 7.8 Hz, 1H), 3.64 (q, *J* = 6.6 Hz, 2H), 3.23-3.09 (m, 2H), 1.95-1.82 (m, 2H); ^{13}C NMR (126 MHz, $CDCl_3$) δ 164.50, 150.10, 148.15, 145.39, 140.24, 137.50, 129.64, 126.22, 122.38, 99.10, 44.50, 39.21, 28.42; HRMS: calculated for $C_{15}H_{14}I_2N_2O$ [M+H$^+$]: 492.9268, found: 492.9281.

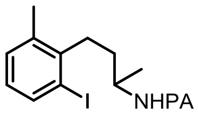

10

Compound **10** was isolated in 75% yield as a colorless oil. ^{1}H NMR (500 MHz, $CDCl_3$) δ 8.54 (d, *J* = 4.1 Hz, 1H), 8.22 (d, *J* = 7.8 Hz, 1H), 8.08 (d, *J* = 8.2 Hz, 1H), 7.84 (td, *J* = 7.7, 1.5 Hz, 1H), 7.64 (d, *J* = 7.8 Hz, 1H), 7.41 (m, 1H), 7.07 (d, *J* = 7.4 Hz, 1H), 6.74 (t, *J* = 7.7 Hz, 1H), 4.40-4.28 (m, 1H), 2.93-2.81 (m, 2H), 2.36 (s, 3H), 1.83-1.69 (m, 2H), 1.37 (d, J = 6.6 Hz, 3H); ^{13}C NMR (126 MHz, $CDCl_3$) δ 163.82, 150.13, 148.08, 142.46, 137.66, 137.46, 137.32, 130.62, 127.83, 126.16, 122.33, 101.93, 45.70, 35.67, 35.06, 21.10, 21.02; HRMS: calculated for $C_{17}H_{19}IN_2O$ [M+H$^+$]: 395.0615; found: 395.0614.

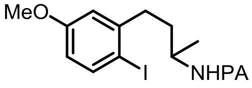

13

Compound **13** was isolated in 68% yield as a colorless oil. ^{1}H NMR (500 MHz, $CDCl_3$) δ 8.54 (d, *J* = 4.3 Hz, 1H), 8.21 (d, *J* = 7.7 Hz, 1H), 8.00 (d, *J* = 8.0 Hz, 1H), 7.84 (t, *J* = 7.2 Hz, 1H), 7.62 (d, *J* = 8.6 Hz, 1H), 7.42 (dd, *J* = 6.6, 5.2 Hz, 1H), 6.82 (d, *J* = 2.7 Hz, 1H), 6.47 (dd, *J* = 8.6, 2.7 Hz, 1H), 4.37-4.20 (m, 1H), 3.75 (s, 3H), 2.88-2.66 (m, 2H), 1.85 (m, 2H), 1.33 (d, *J* = 6.6 Hz, 3H); ^{13}C NMR (126 MHz, $CDCl_3$) δ 163.76, 160.16, 150.15, 148.07, 145.43, 139.94, 137.48, 126.18, 122.35, 115.47, 114.10, 88.91, 55.43, 45.20, 37.84, 37.55, 21.22; HRMS: calculated for $C_{17}H_{19}IN_2O_2$ [M+H$^+$]: 411.0564, found: 411.0564.

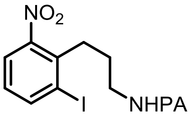

16

Compound **16** was isolated in 68% yield as a colorless oil. ^{1}H NMR (500 MHz, $CDCl_3$) δ 8.55 (d, *J* = 4.2 Hz, 1H), 8.24 (s, 1H), 8.21 (d, *J* = 7.8 Hz, 1H), 8.05 (d, *J* = 7.8 Hz, 1H), 7.85 (td, *J* = 7.7, 1.5 Hz, 1H), 7.73 (d, *J* = 8.0 Hz, 1H), 7.42 (dd, *J* = 6.6, 4.9 Hz, 1H), 7.04 (t, *J* = 8.0 Hz, 1H), 3.64 (q, *J* = 6.6 Hz, 2H), 3.04-2.94 (m, 2H), 2.11-1.97 (m, 2H); ^{13}C NMR (126 MHz, $CDCl_3$) δ

164.56, 150.38, 149.99, 148.16, 144.03, 137.92, 137.51, 128.58, 126.25, 124.54, 122.37, 102.96, 39.29, 35.39, 29.30; HRMS: calculated for $C_{15}H_{14}IN_3O_3$ [M+H$^+$]: 412.0153; found: 412.0153.

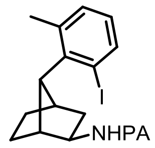

19

Compound **19** was isolated in 56% yield as a colorless oil. ^{1}H NMR (400 MHz, $CDCl_3$) δ 8.16 (d, *J* = 4.1 Hz, 1H), 7.99 (d, *J* = 7.8 Hz, 1H), 7.71 (d, *J* = 7.0 Hz, 1H), 7.70 (td, *J* = 7.7, 1.6 Hz, 1H), 7.27-7.24 (m, 2H), 7.09 (s, 1H), 6.75 (t, *J* = 7.3 Hz, 1H), 4.06 (td, *J* = 8.8, 3.0 Hz, 1H), 3.27 (s, 1H), 3.18 (s, 1H) 2.99 (s, 1H), 2.56 (s, 3H), 2.16-1.89 (m, 3H), 1.80-1.77 (m, 1H), 1.42-1.37 (m, 2H); ^{13}C NMR (101 MHz, $CDCl_3$) δ 162.89, 149.86, 147.46, 139.42, 136.99, 133.51, 127.19, 125.68, 121.70, 60.21, 52.22, 49.65, 41.22, 38.70, 30.65, 25.22, 23.14.

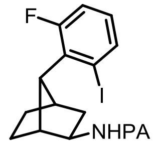

22

Compound **22** was isolated in 53% yield as a white solid and the yield of **22** was improved to 85% when 1.0 equiv of Na_2CO_3 was used instead of $KHCO_3$. ^{1}H NMR (500 MHz, $CDCl_3$) δ 8.13 (d, *J* = 4.0 Hz, 1H), 8.01 (d, *J* = 7.8 Hz, 1H), 7.71 (td, *J* = 7.7, 1.6 Hz, 1H), 7.64 (d, *J* = 7.7 Hz, 1H), 7.39 (d, *J* = 5.5 Hz, 1H), 7.32-7.19 (m, 1H), 7.04 (dd, *J* = 11.4, 8.7 Hz, 1H), 6.84 (td, *J* = 7.9, 5.5 Hz, 1H), 4.05 (dd, *J* = 14.0, 6.0 Hz, 1H), 3.30 (d, *J* = 4.5 Hz, 1H), 2.98 (s, 1H), 2.88 (s, 1H), 2.08 (m, 2H), 1.95-1.80 (m, 1H), 1.80-1.61 (m, 1H), 1.45-1.31 (m, 2H); ^{13}C NMR (126 MHz, $CDCl_3$) δ 162.81, 160.60, 158.60, 149.80, 147.37, 137.12, 136.89, 136.87, 129.87, 129.75, 128.84, 128.77, 125.71, 121.77, 117.65, 117.44, 102.56, 102.52, 56.76, 56.73, 52.71, 47.92, 40.48, 40.40, 39.06, 39.00, 29.99, 25.49; HRMS: calculated for $C_{19}H_{18}FIN_2O$ [M+H$^+$]: 437.0521; found: 437.0516.

7. Preparation of iodinated compounds via directed electrophilic aromatic iodination

Directed electrophilic aromatic iodination reactions were carried out following the literature protocol [1]: Picolinamide substrate (0.2 mmol, 1 equiv) and NIS (49.5 mg, 0.22 mmol, 1.1 equiv) were dissolved in anhydrous CH_2Cl_2 (27 mL) and cooled to 0 °C under Ar. TFA (3 mL) and HBF_4•Et_2O (0.11 mL, 0.8 mmol, 4 equiv) were added, and the reaction mixture was stirred at 0 °C for 4 hours. Solvents were then removed in vacuo or with stream of N_2. The residue was redissolved in CH_2Cl_2 (15 mL), washed with aq $NaHCO_3$, aq $Na_2S_2O_3$, brine, dried over anhydrous Na_2SO_4, and concentrated in vacuo. The resulting residue was purified by silica gel flash chromatography to give the iodinated products. Yields of *ortho*-iodinated products were obtained using ^{1}H NMR with 1,1,2,2-tetrachloroethane standard for substrates **2**, **9**, **12**, **15**, and **18**.

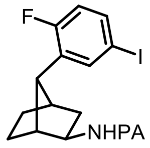

23

Compound **23** was isolated via silica gel flash chromatography in 90% yield as a white solid. ^{1}H NMR (500 MHz, $CDCl_3$) δ 8.26 (d, *J* = 4.2 Hz, 1H), 8.03 (d, *J* = 7.8 Hz, 1H), 7.72 (td, *J* = 7.7, 1.5 Hz, 1H), 7.61 (d, *J* = 6.0 Hz, 1H), 7.51-7.44 (m, 1H), 7.34 (d, *J* = 7.5 Hz, 1H), 7.29 (dd, *J* = 6.7, 4.9 Hz, 1H), 6.66 (dd, J = 10.3, 8.7 Hz, 1H), 4.05 (td, *J* = 8.5, 3.7 Hz, 1H), 2.92 (s, 1H), 2.90 (s, 1H), 2.79 (s, 1H), 2.00 (dd, *J* = 13.5, 8.7 Hz, 1H), 1.82 (m, 1H), 1.78-1.63 (m, 2H), 1.52-1.39 (m, 1H), 1.39-1.28 (m, 1H); ^{13}C NMR (126 MHz, $CDCl_3$) δ 162.89, 162.62, 160.65, 149.65, 147.66, 138.43, 138.39, 137.08), 136.80, 136.74, 130.69, 130.57, 125.80, 121.70, 118.21,

118.02, 87.51, 87.48, 52.84, 48.36, 46.39, 46.36, 38.03, 37.86, 37.85, 28.46, 27.50; HRMS: calculated for $C_{19}H_{18}FIN_2O$ [M+H$^+$]: 437.0521; found: 437.0520.

8. Preparation of tetrahydroquinolines via Cu-catalyzed cyclization[4]

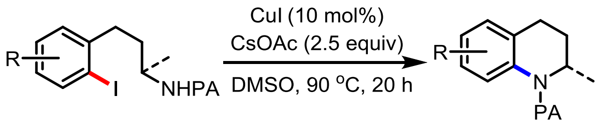

Scheme S4

A mixture of *ortho*-iodinated compound (0.2 mmol, 1.0 equiv), copper iodide (3.8 mg, 0.02 mmol, 0.1 equiv), and CsOAc (96 mg, 0.5 mmol, 2.5 equiv) in DMSO (2 mL) was heated at 90 °C under Ar for 20 hours. The reaction mixture was cooled to rt, diluted with EtOAc, washed with water and brine. The organic phase was dried over anhydrous Na_2SO_4, filtered and the filtration was concentrated in vacuo. The resulting residue was purified by silica gel flash chromatography to give the cyclized product.

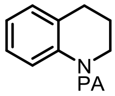

8

Compound **8** was isolated in 93% yield as a white solid. Compound **8** has been previously reported and its spectra are consistent with those from the literature.[1]

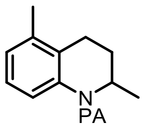

11

Compound **11** was isolated in 96% yield as a colorless oil. ^{1}H NMR (500 MHz, $CDCl_3$) δ 8.43 (d, *J* = 1.8 Hz, 1H), 7.55 (s, 1H), 7.26 (s, 1H), 7.16 (s, 1H), 6.82 (d, *J* = 7.3 Hz, 1H), 6.69 (s, 1H), 6.40 (s, 1H), 4.83 (s, 1H), 2.72 (m, 1H), 2.65-2.47 (m, 1H), 2.32 (m, 1H), 2.22 (s, 3H), 1.58 (m, 1H), 1.19 (d, *J* = 6.6 Hz, 3H); ^{13}C NMR (126 MHz, $CDCl_3$) δ 167.85, 155.05, 148.83, 136.64, 136.31, 135.54, 130.31, 126.38, 125.02, 124.00, 123.95, 123.26, 48.46, 30.56, 21.74, 19.2, 18.36; HRMS: calculated for $C_{17}H_{18}N_2O$ [M+H$^+$]: 267.1492; found: 267.1495.

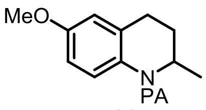

14

Compound **14** was isolated in 94% yield as a pale yellow oil. ^{1}H NMR (500 MHz, $CDCl_3$) δ 8.50 (s, 1H), 7.60 (s, 1H), 7.21 (s, 2H), 6.69 (s, 1H), 6.34 (s, 2H), 4.90 (s, 1H), 3.72 (s, 3H), 2.73 (m, 2H), 2.43 (s, 1H), 1.49 (s, 1H), 1.25 (d, *J* = 6.4 Hz, 3H); ^{13}C NMR (126 MHz, $CDCl_3$) δ 167.49, 157.00, 155.13, 148.99, 136.56, 127.13, 124.11, 123.40, 112.88), 111.47, 55.43, 49.34, 32.31, 26.45, 19.87; HRMS: calculated for $C_{17}H_{18}N_2O_2$ [M+H$^+$]: 283.1441; found: 283.1442.

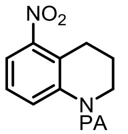

17

Compound **17** was isolated in 47% yield as a yellow solid. ^{1}H NMR (500 MHz, $CDCl_3$) δ 8.48 (s, 1H), 7.79 (t, *J* = 7.4 Hz, 1H), 7.68 (d, *J* = 7.6 Hz, 1H), 7.63 (d, *J* = 8.1 Hz, 1H), 7.38-7.29 (m, 1H), 7.14 (s, 1H), 7.08 (s, 1H), 3.94 (t, *J* = 6.0 Hz, 2H), 3.14 (t, *J* = 6.6 Hz, 2H), 2.06 (m, 2H); ^{13}C NMR (126 MHz, $CDCl_3$) δ 168.75, 153.76, 149.48, 148.78, 140.83, 137.28), 129.69, 125.85, 125.18, 124.40, 120.79, 44.98, 23.78, 23.30; HRMS: calcullated for $C_{15}H_{13}N_3O_3$ [M+H$^+$]: 284.1030; found: 284.1030.

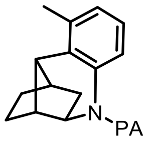

20

Compound **20** was isolated in 85% yield as an off white solid. ^{1}H NMR (500 MHz, $CDCl_3$) δ 8.59 (s, 1H), 7.77 (s, 1H), 7.57 (s, 1H), 7.32 (s, 1H), 6.87 (s, 2H), 2.91 (s, 1H), 2.42 (s, 1H), 2.31 (m, 4H), 1.93-1.71 (m, 2H), 1.72-1.58 (m, 1H), 1.53 (dd, *J* = 13.5, 7.3 Hz, 2H); ^{13}C NMR (126 MHz, $CDCl_3$) δ 167.44, 155.51, 148.97, 137.09, 136.04, 135.91, 129.63, 126.06, 125.73, 124.47, 123.68, 121.58, 46.97, 44.67, 39.78, 34.46, 29.81, 29.19, 24.89, 19.58; HRMS: calculated for $C_{20}H_{20}N_2O$ [M+H$^+$]: 305.1649; found: 305.1650.

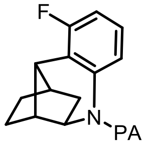

20

Compound 24 was isolated in 78% yield as a colorless oil. ^{1}H NMR (400 MHz, $CDCl_3$) δ 8.59 (d, *J* = 4.0 Hz, 1H), 7.81-7.77 (m, 1H), 7.62 (d, *J* = 7.6 Hz, 1H), 7.35 (dd, *J* = 6.9, 5.1 Hz, 1H), 7.01 (s, 1H), 6.74 (t, *J* = 8.6 Hz, 1H), 4.42-4.38 (m, 1H), 3.08 (s, 1H), 2.50 (s, 1H), 2.27 (s, 1H), 1.84-1.79 (m, 2H), 1.57-1.63 (m, 1H), 1.56-1.52 (m, 1H), 1.26-1.21 (m, 2H); ^{13}C NMR (101 MHz, $CDCl_3$) δ 167.72, 161.40, 158.99, 154.98, 148.95, 137.64, 137.19, 126.79, 124.75, 123.77, 119.02, 118.82, 118.71, 118.68, 110.52, 110.30, 45.51, 42.71, 38.82, 34.77, 29.15, 24.64; HRMS Calcd for $C_{19}H_{17}FN_2O$ [M+H$^+$]: 309.1398, Found: 309.1402.

9. Preparation of iodinated THQs

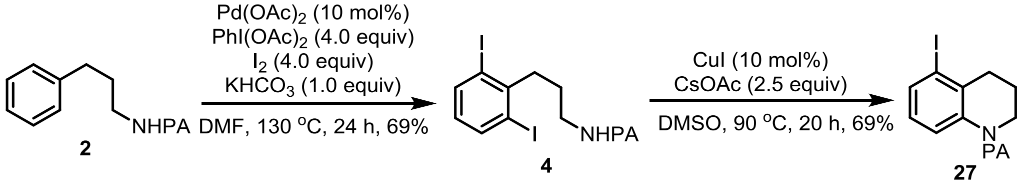

Scheme S5

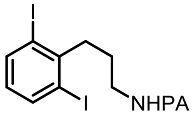

4

Compound **2** (48 mg, 0.2 mmol, 1.0 equiv), $PhI(OAc)_2$ (260 mg, 0.8 mmol, 4.0 equiv), I_2 (200 mg, 0.8 mmol, 4.0 equiv), $Pd(OAc)_2$ (4.5 mg, 0.02 mmol, 0.1 equiv), and $KHCO_3$ (20 mg, 0.2 mmol, 1.0 equiv) were dissolved in anhydrous DMF (6 mL, 0.03 M) in a 10 mL vial. 4 Å molecular sieves (200 mg) was added and the reaction vial was purged with Ar and sealed with a PTFE cap. The mixture was stirred at 25 °C for 1 h, then heated to 130 °C for 24 h. The reaction mixture was cooled to room temperature, diluted with EtOAc, then washed with aq $Na_2S_2O_3$ (sat.), H_2O, and brine. The organic layer was dried over anhydrous Na_2SO_4, filtered, and concentrated in vacuo. The resulting residue was purified by silica gel flash chromatography to give **4** in 69% yield as a white solid.

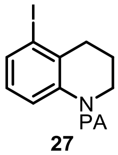

27

Compound **27** was obtained from compound **4** using the standard Cu-catalyzed cross coupling protocol in 69% yield as a white solid. ^{1}H NMR (500 MHz, $CDCl_3$) δ 8.50 (s, 1H), 7.74 (t, *J* =

7.2 Hz, 1H), 7.56 (s, 2H), 7.39-7.27 (m, 1H), 7.15-6.70 (m, 1H), 6.62 (s, 1H), 3.89 (s, 2H), 2.85 (t, J = 6.6 Hz, 2H), 2.24-1.85 (m, 2H); ^{13}C NMR (126 MHz, $CDCl_3$) δ 168.24, 154.39, 148.84, 139.78, 137.03, 135.60, 133.47, 127.04, 125.37, 124.77, 124.08, 101.42, 44.72, 33.65, 24.56; HRMS: calculated for $C_{15}H_{13}IN_2O$ [M+H$^+$]: 365.0145, found: 365.0150.

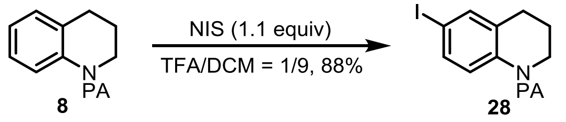

Scheme S6

Compound **28** was prepared from compound **8** using the NIS-mediated S_EAr iodination protocol: Picolinamide substrate **8** (47.6 mg, 0.2 mmol, 1 equiv) and NIS (49.5 mg, 0.22 mmol, 1.1 equiv) were dissolved in a mixture of anhydrous CH_2Cl_2 (27 mL) and TFA (3 mL) at room temperature. After 16 hours, solvents were removed in vacuo or with a stream of N_2. The residue was redissolved in CH_2Cl_2 (15 mL), washed with aq $NaHCO_3$, aq $Na_2S_2O_3$, brine, dried over anhydrous Na_2SO_4, and concentrated in vacuo. The resulting residue was purified by silica gel flash chromatography to give the iodinated products in 88% yield as a yellow solid. ^{1}H NMR (500 MHz, $CDCl_3$) δ 8.49 (s, 1H), 7.73 (t, J = 7.4 Hz, 1H), 7.55 (d, J = 7.2 Hz, 1H), 7.47 (s, 1H), 7.33-7.27 (m, 1H), 7.19 (s, 1H), 6.94-6.17 (m, 1H), 3.88 (s, 2H), 2.80 (t, J = 6.6 Hz, 2H), 2.09-1.94 (m, 2H); ^{13}C NMR (126 MHz, $CDCl_3$) δ 168.31, 154.35, 148.87, 137.51, 137.02, 134.67, 126.53, 124.78, 123.98, 88.85, 26.71, 23.57; HRMS: calculated for $C_{15}H_{13}IN_2O$ [M+H$^+$]: 365.0145; found: 365.0146.

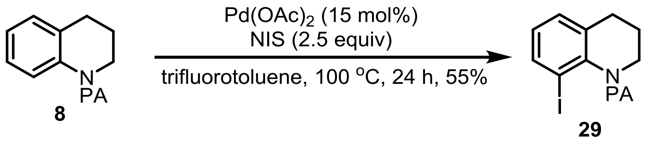

Scheme S7

Compound **8** (48 mg, 0.2 mmol, 1.0 equiv), NIS (113 mg, 0.5 mmol, 2.5 equiv), and $Pd(OAc)_2$ (6.5 mg, 0.03 mmol, 0.015 equiv) were dissolved in α,α,α-trifluorotoluene (2 mL, 0.1 M) in a 10 mL vial. The vial was purged with Ar, sealed with a PTFE cap, and heated to 100 °C. After 24 h, the reaction was cooled to room temperature, filtered through a pad of celite and eluted with EtOAc. The filtrate was washed with aq $Na_2S_2O_3$ (sat.), dried over anhydrous Na_2SO_4, and concentrated in vacuo. The resulting residue was purified by silica gel flash chromatography to give **29** as a white solid (40 mg, 55%). The NMR spectrum of **29** at room temperature in $CDCl_3$ represents a 2:1 mixture of rotamers. ^{1}H NMR (500 MHz, $CDCl_3$) δ 8.72 (s, 1H), 8.06 (d, *J* = 3.8 Hz, 2H), 7.93-7.80 (m, 4H), 7.77 (d, *J* = 7.9 Hz, 1H), 7.66 (t, *J* = 7.2 Hz, 2H), 7.46-7.34 (m, 3H), 7.24-7.08 (m, 6H), 6.90 (t, *J* = 7.7 Hz, 1H), 6.82 (t, *J* = 7.7 Hz, 2H), 4.78 (m, 2H), 4.25-4.13 (m, 1H), 3.54-3.41 (m, 1H), 3.21-3.06 (m, 2H), 2.90-2.72 (m, 6H), 2.47-2.32 (m, 2H), 2.17-2.03 (m, 1H), 1.87-1.64 (m, 3H); ^{13}C NMR (126 MHz, $CDCl_3$) δ 168.48, 167.54, 154.31, 153.92, 148.91, 147.10, 143.54, 141.59, 138.79, 138.23, 137.76, 137.19, 137.06, 136.62, 136.00, 128.52, 127.83, 127.72, 127.43, 125.86, 125.31, 124.73, 96.30, 94.76, 47.00, 42.69, 27.76, 26.62, 25.02, 24.54; HRMS: calculated for $C_{15}H_{13}IN_2O$ $[M+H^+]$: 365.0145, found: 365.0145.

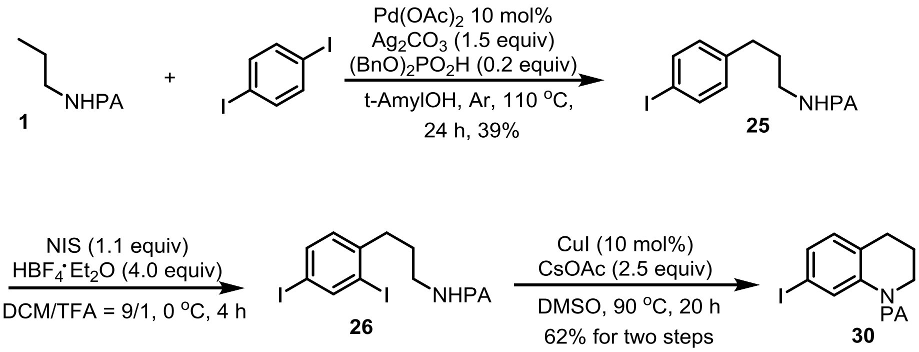

Scheme S8

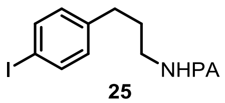

25

Compound **25** was isolated in 39% yield as a pale yellow oil using C-H arylation conditions **A**. ^{1}H NMR (500 MHz, $CDCl_3$) δ 8.54 (d, *J* = 4.3 Hz, 1H), 8.19 (d, *J* = 7.8 Hz, 1H), 8.07 (s, 1H), 7.84 (td, *J* = 7.7, 1.6 Hz, 1H), 7.59 (d, *J* = 8.2 Hz, 2H), 7.42 (m, 1H), 6.96 (d, *J* = 8.2 Hz, 2H), 3.50 (m, 2H), 2.73-2.60 (m, 2H), 2.00-1.89 (m, 2H); ^{13}C NMR (126 MHz, $CDCl_3$) δ 164.43, 150.01, 148.16, 141.23, 137.59, 137.50, 130.65, 126.26, 122.30, 91.13, 39.00, 32.95, 31.20; HRMS: calculated for $C_{15}H_{16}IN_2O$ [M+H$^+$] : 367.0302, found: 367.0305.

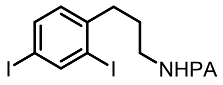

26

Compound **26** was prepared via the standard directed electrophilic aromatic iodination protocol, and was applied directly to the next step.

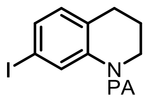

30

Compound **30** was isolated in 62% yield (over 2 steps) as a colorless oil via the standard cross coupling protocol. ^{1}H NMR (500 MHz, $CDCl_3$) δ 8.51 (s, 1H), 7.77 (t, *J* = 7.5 Hz, 1H), 7.58 (d, *J*

= 7.0 Hz, 1H), 7.40-7.27 (m, 2H), 7.24-6.93 (m, 1H), 6.86 (d, *J* = 8.0 Hz, 1H), 3.88 (t, *J* = 5.6 Hz, 2H), 2.80 (t, *J* = 6.6 Hz, 2H), 2.12-1.95 (m, 2H); ^{13}C NMR (126 MHz, $CDCl_3$) δ 168.38, 154.29, 148.82, 139.98, 137.05, 133.56, 133.55, 130.31, 124.88, 124.04, 89.49, 44.97, 26.79, 23.59; HRMS: calculated for $C_{15}H_{13}IN_2O$ [M+H$^+$]: 365.0145, found: 365.0147.

10. Removal of PA group

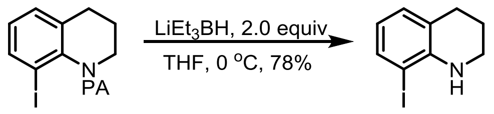

Scheme S9

Compound **29** (0.3 mmol, 70 mg, 1.0 equiv) was dissolved in anhydrous THF (2 mL) in an oven-dried 10 mL vial at 0 °C, and $LiEt_3BH$ (0.6 mL of 1 M solution in THF) was added. The mixture was stirred at 0 °C for 4 h, then aq NH_4Cl (sat.) followed by 1 M NaOH (aq) was added. The mixture was extracted with CH_2Cl_2, the combined organic layers were dried with anhydrous Na_2SO_4 and concentrated in vacuo. The resulting residue was purified by silica gel flash chromatography (2% ether in hexanes) to give **31** as a pale yellow oil in 78% yield. ^{1}H NMR (500 MHz, $CDCl_3$) δ 7.47 (d, *J* = 7.8 Hz, 1H), 6.91 (d, *J* = 7.2 Hz, 1H), 6.34 (t, *J* = 7.6 Hz, 1H), 4.30 (bs, 1H), 3.49-3.31 (m, 2H), 2.75 (t, *J* = 6.3 Hz, 2H), 2.00-1.82 (m, 2H); ^{13}C NMR (126 MHz, $CDCl_3$) δ 144.10, 136.72, 129.59, 122.49, 118.10, 84.67, 42.68, 27.84, 21.98; HRMS: calculated for $C_9H_{10}IN$ [M+H$^+$]: 259.9931, found: 259.9932.

11. Crystallographic data of 23

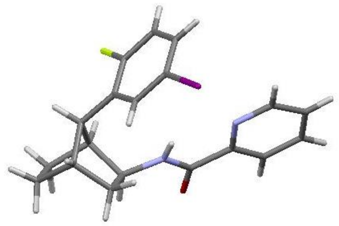

Figure S1. ORTEP view of compound **23**

The X-ray structure is deposited in the Cambridge Crystallographic Data Centre CCDC 1466492.

12. References

(1) Nack, W. A.; He, G.; Zhang, S.; Lu, C.; Chen, G. *Org. Lett.* **2013**, *15*, 3440-3443.

(2) He, G.; Chen, G. *Angew. Chem., Int. Ed.* **2011**, *50*, 5192-5196.

(3) Zaitsev, V. G.; Shabashov, D.; Daugulis, O. *J. Am. Chem. Soc.* **2005**, *127*, 13154-13155

(4) Kubo, T.; Katoh, C.; Yamada, K.; Okano, K.; Tokuyama, H.; Fukuyama, T. *Tetrahedron* **2008**, *64*, 11230-11236.

13. Spectra

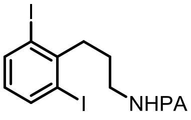

I
I
NHPA

4

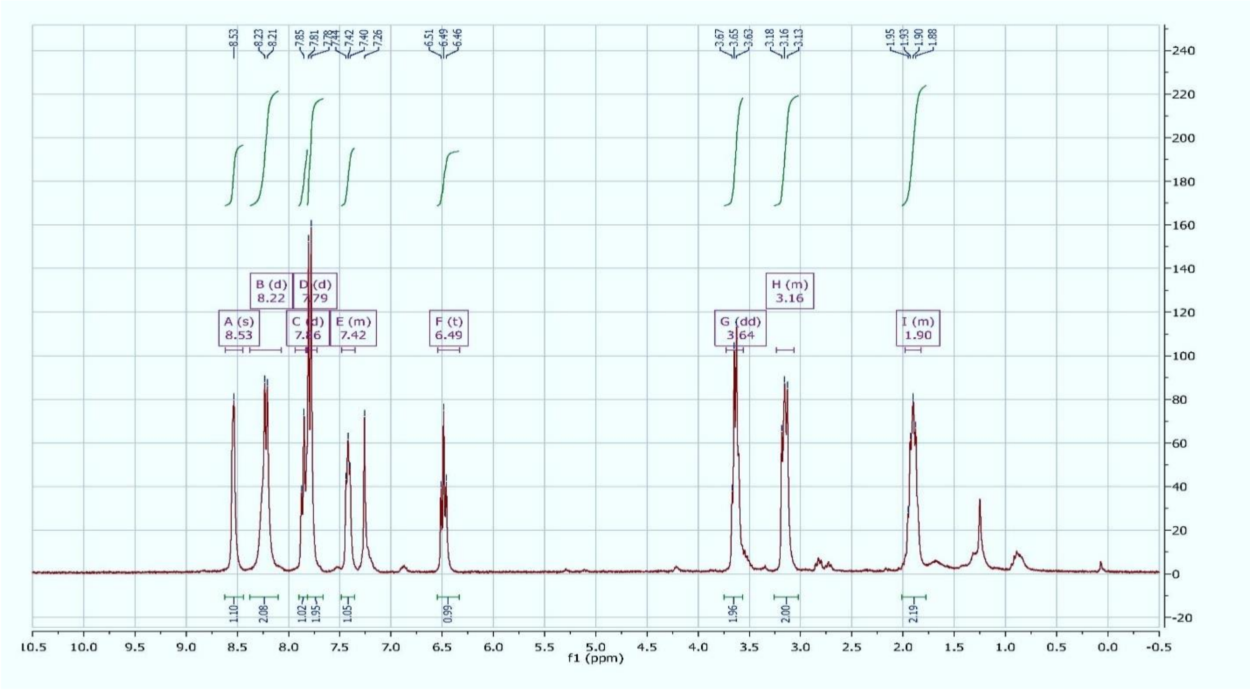

A (s) 8.53
B (d) 8.22
C (d) 7.86
D (d) 7.79
E (m) 7.42
F (t) 6.49
G (dd) 3.64
H (m) 3.16
I (m) 1.90
f1 (ppm)

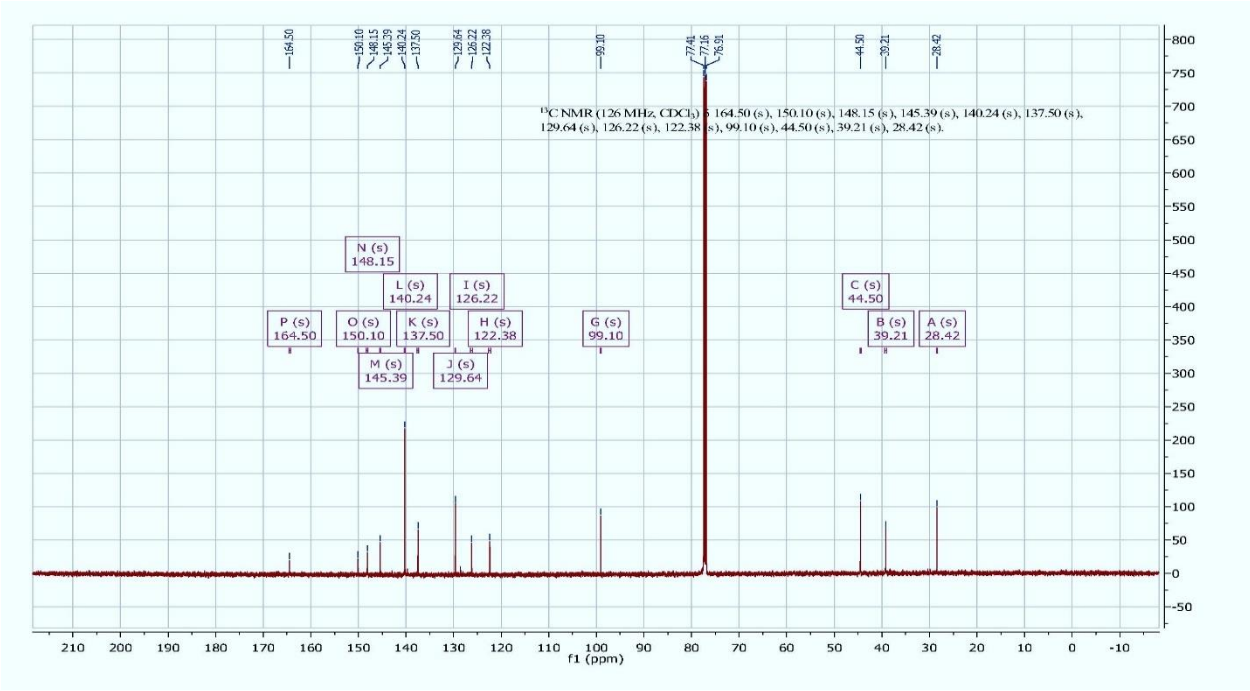

13C NMR (126 MHz, CDCl3) δ 164.50 (s), 150.10 (s), 148.15 (s), 145.39 (s), 140.24 (s), 137.50 (s), 129.64 (s), 126.22 (s), 122.38 (s), 99.10 (s), 44.50 (s), 39.21 (s), 28.42 (s).
f1 (ppm)

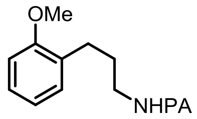
OMe
NHPA

5

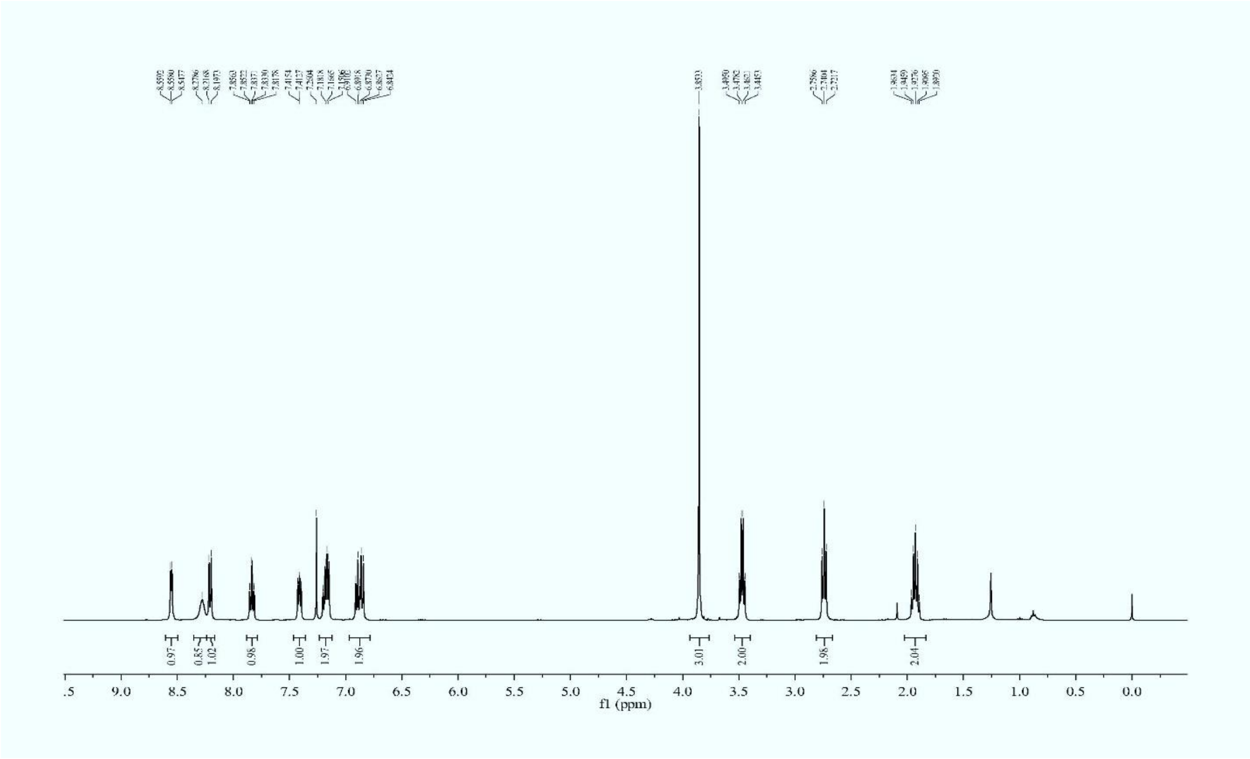
0.97
0.85
1.02
0.98
1.00
1.97
1.96
3.01
2.00
1.96
2.04
.5 9.0 8.5 8.0 7.5 7.0 6.5 6.0 5.5 5.0 4.5 4.0 3.5 3.0 2.5 2.0 1.5 1.0 0.5 0.0
f1 (ppm)

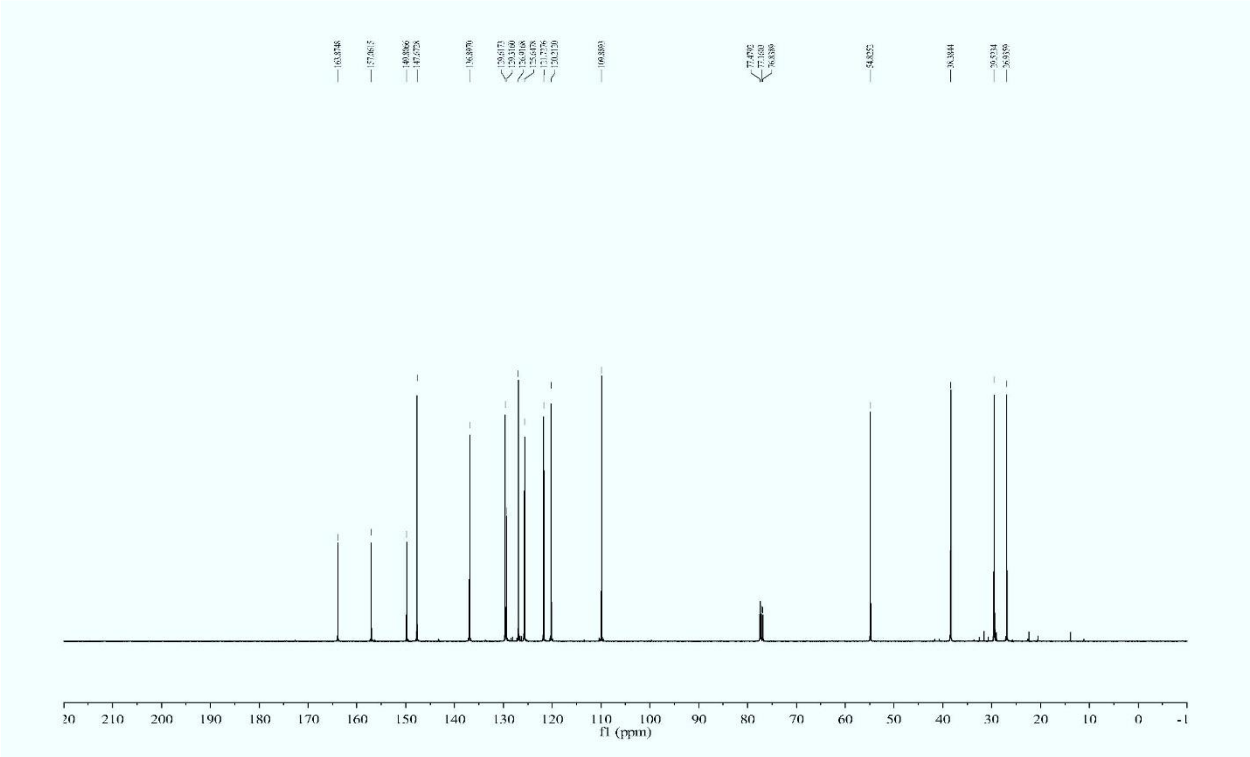
20 210 200 190 180 170 160 150 140 130 120 110 100 90 80 70 60 50 40 30 20 10 0 -1
f1 (ppm)

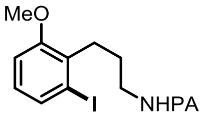

6

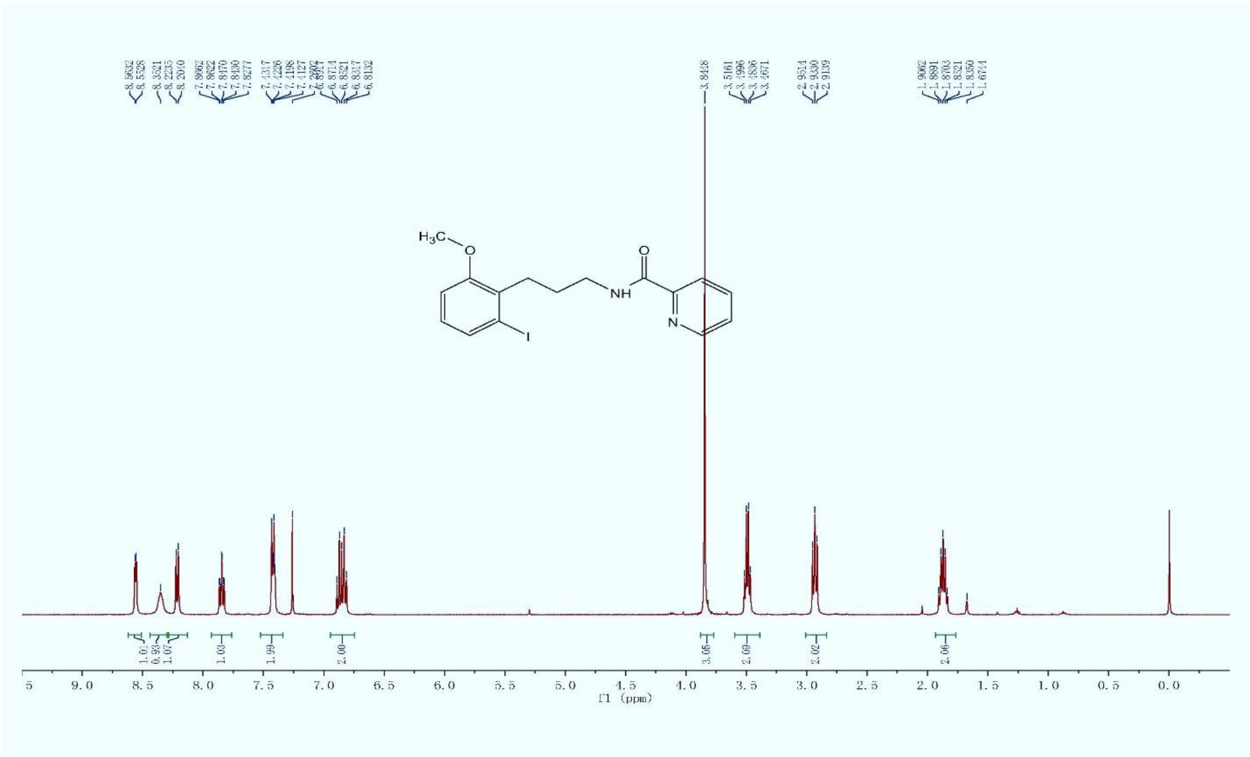

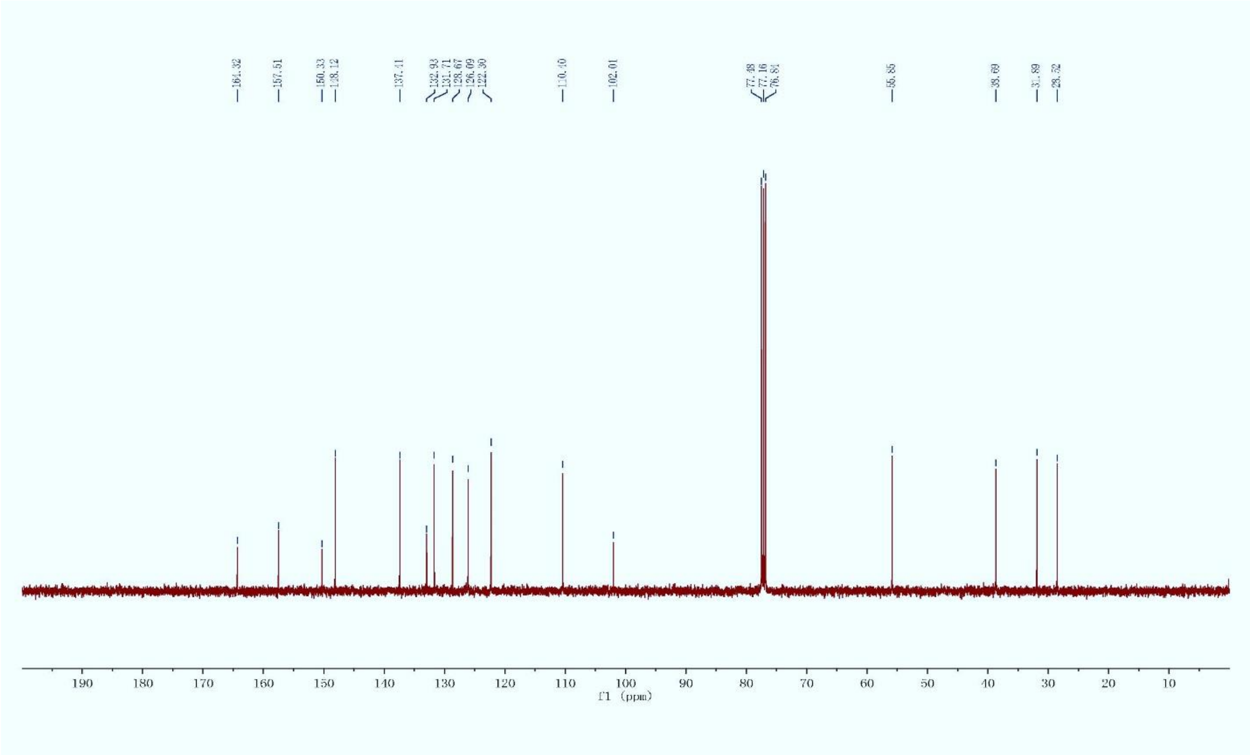

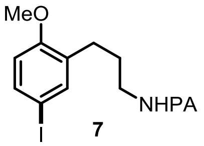

7

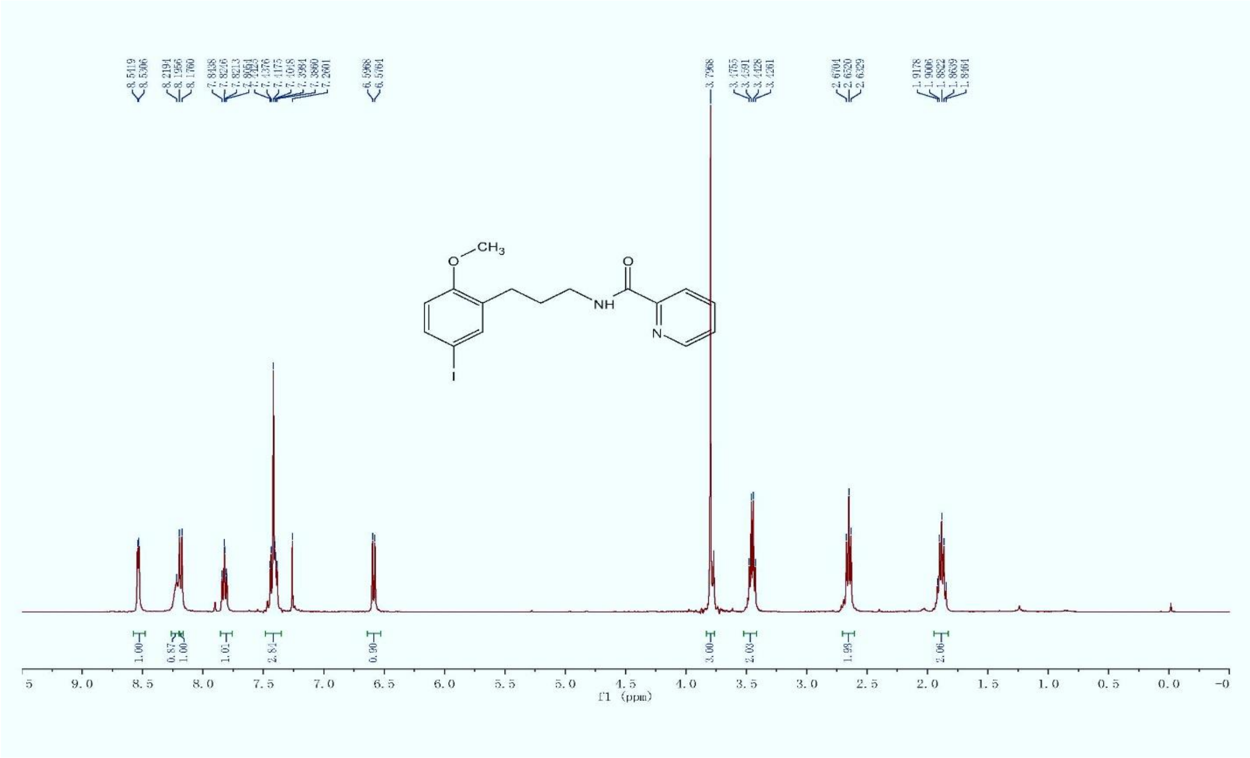

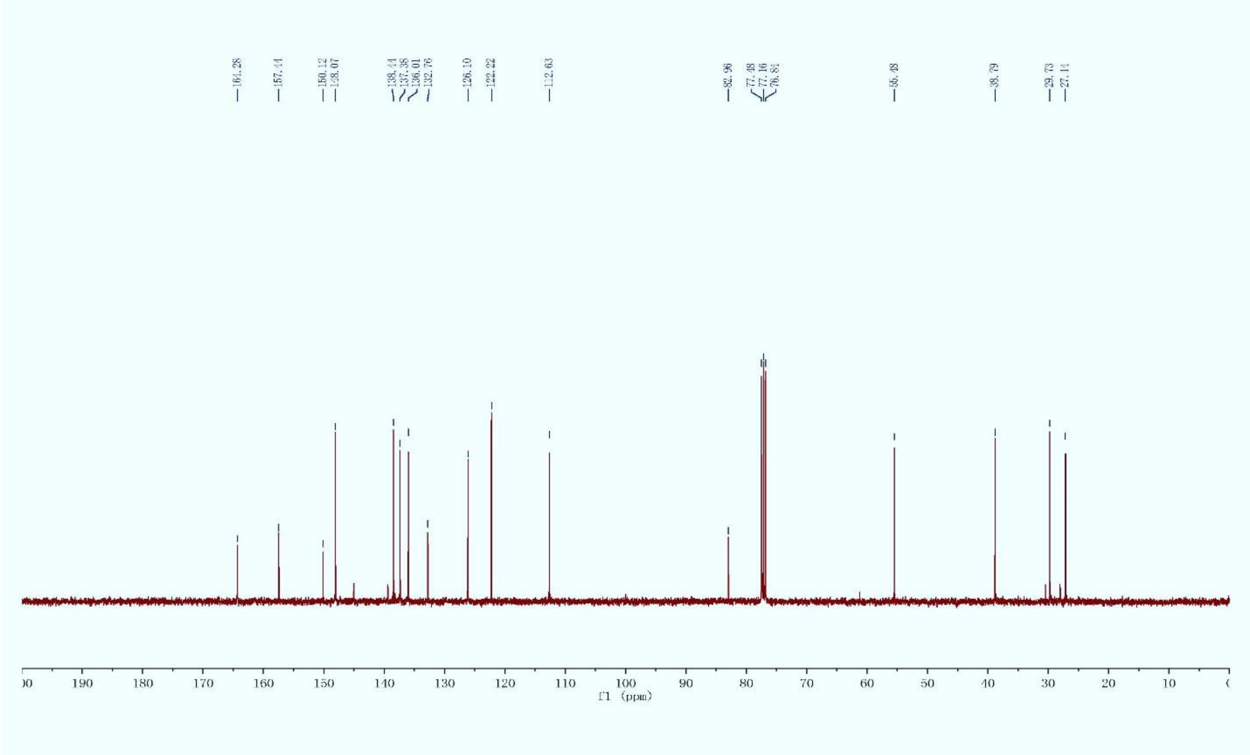

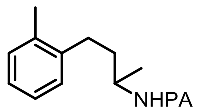
NHPA

9

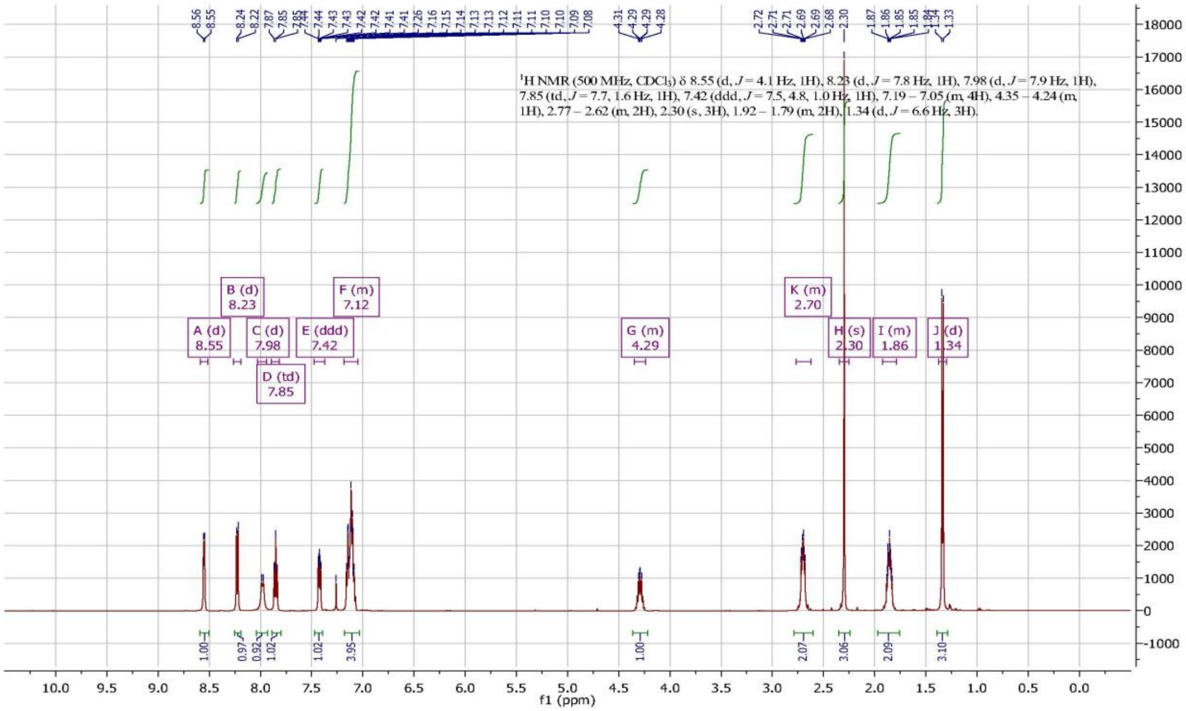
¹H NMR (500 MHz, CDCl₃) δ 8.55 (d, J = 4.1 Hz, 1H), 8.23 (d, J = 7.8 Hz, 1H), 7.98 (d, J = 7.9 Hz, 1H), 7.85 (td, J = 7.7, 1.6 Hz, 1H), 7.42 (ddd, J = 7.5, 4.8, 1.0 Hz, 1H), 7.19 – 7.05 (m, 4H), 4.35 – 4.24 (m, 1H), 2.77 – 2.62 (m, 2H), 2.30 (s, 3H), 1.92 – 1.79 (m, 2H), 1.34 (d, J = 6.6 Hz, 3H).
A (d) 8.55
B (d) 8.23
C (d) 7.98
D (td) 7.85
E (ddd) 7.42
F (m) 7.12
G (m) 4.29
K (m) 2.70
H (s) 2.30
I (m) 1.86
J (d) 1.34
f1 (ppm)

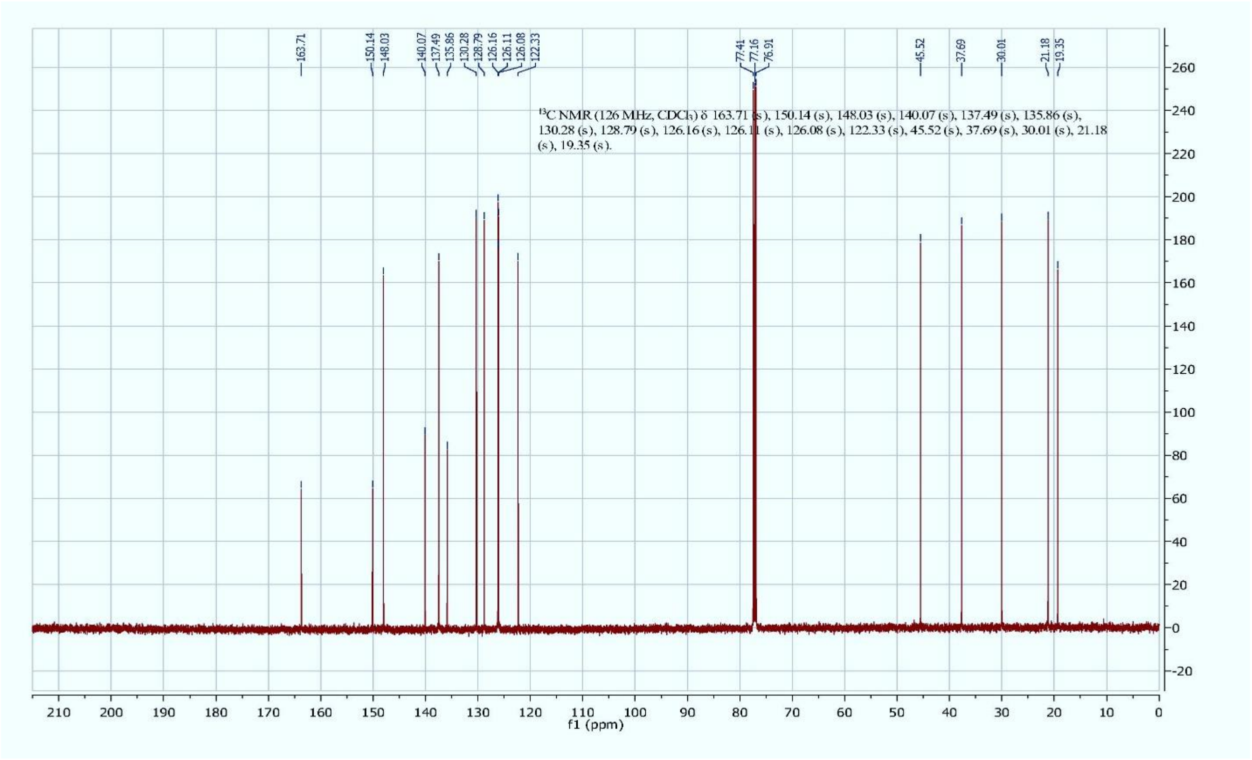
¹³C NMR (126 MHz, CDCl₃) δ 163.71 (s), 150.14 (s), 148.03 (s), 140.07 (s), 137.49 (s), 135.86 (s), 130.28 (s), 128.79 (s), 126.16 (s), 126.11 (s), 126.08 (s), 122.33 (s), 45.52 (s), 37.69 (s), 30.01 (s), 21.18 (s), 19.35 (s).
f1 (ppm)

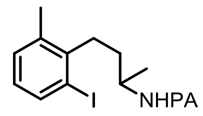
I
NHPA

10

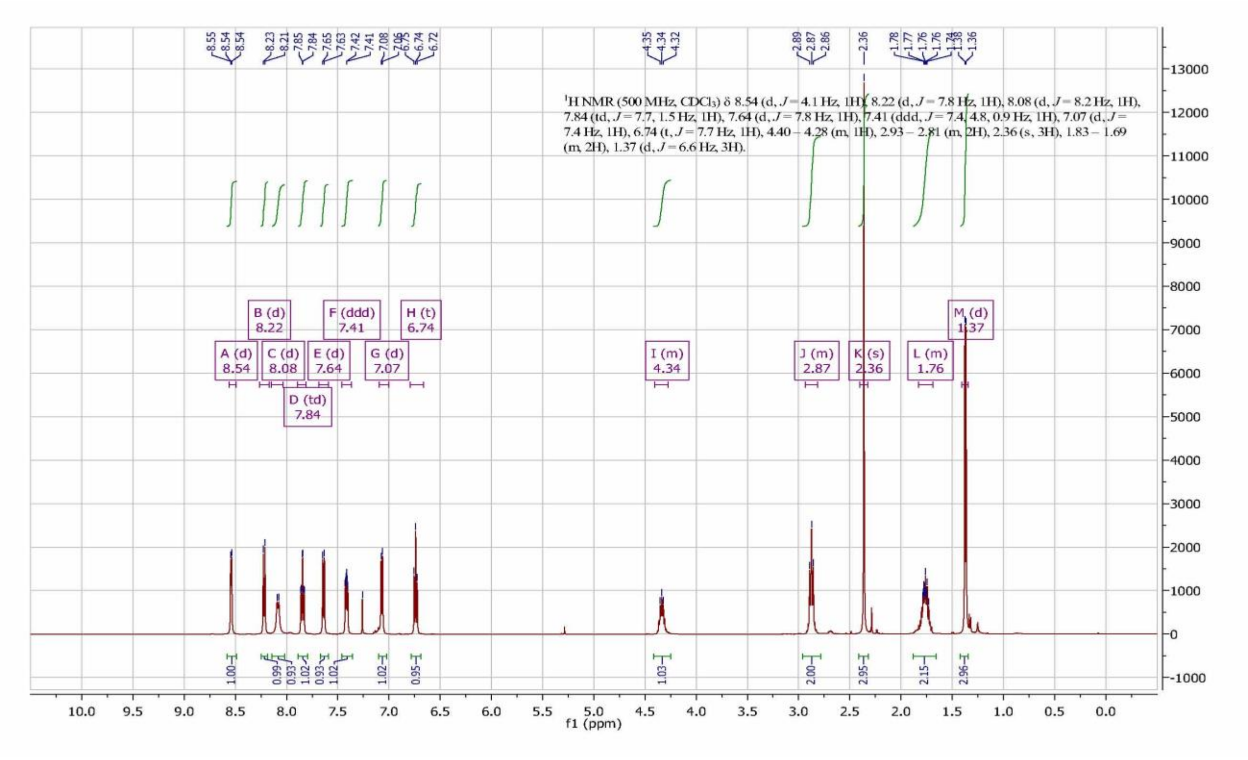
1H NMR (500 MHz, CDCl3) δ 8.54 (d, J = 4.1 Hz, 1H), 8.22 (d, J = 7.8 Hz, 1H), 8.08 (d, J = 8.2 Hz, 1H), 7.84 (td, J = 7.7, 1.5 Hz, 1H), 7.64 (d, J = 7.8 Hz, 1H), 7.41 (ddd, J = 7.4, 4.8, 0.9 Hz, 1H), 7.07 (d, J = 7.4 Hz, 1H), 6.74 (t, J = 7.7 Hz, 1H), 4.40 – 4.28 (m, 1H), 2.93 – 2.81 (m, 2H), 2.36 (s, 3H), 1.83 – 1.69 (m, 2H), 1.37 (d, J = 6.6 Hz, 3H).
A (d) 8.54
B (d) 8.22
C (d) 8.08
D (td) 7.84
E (d) 7.64
F (ddd) 7.41
G (d) 7.07
H (t) 6.74
I (m) 4.34
J (m) 2.87
K (s) 2.36
L (m) 1.76
M (d) 1.37
f1 (ppm)

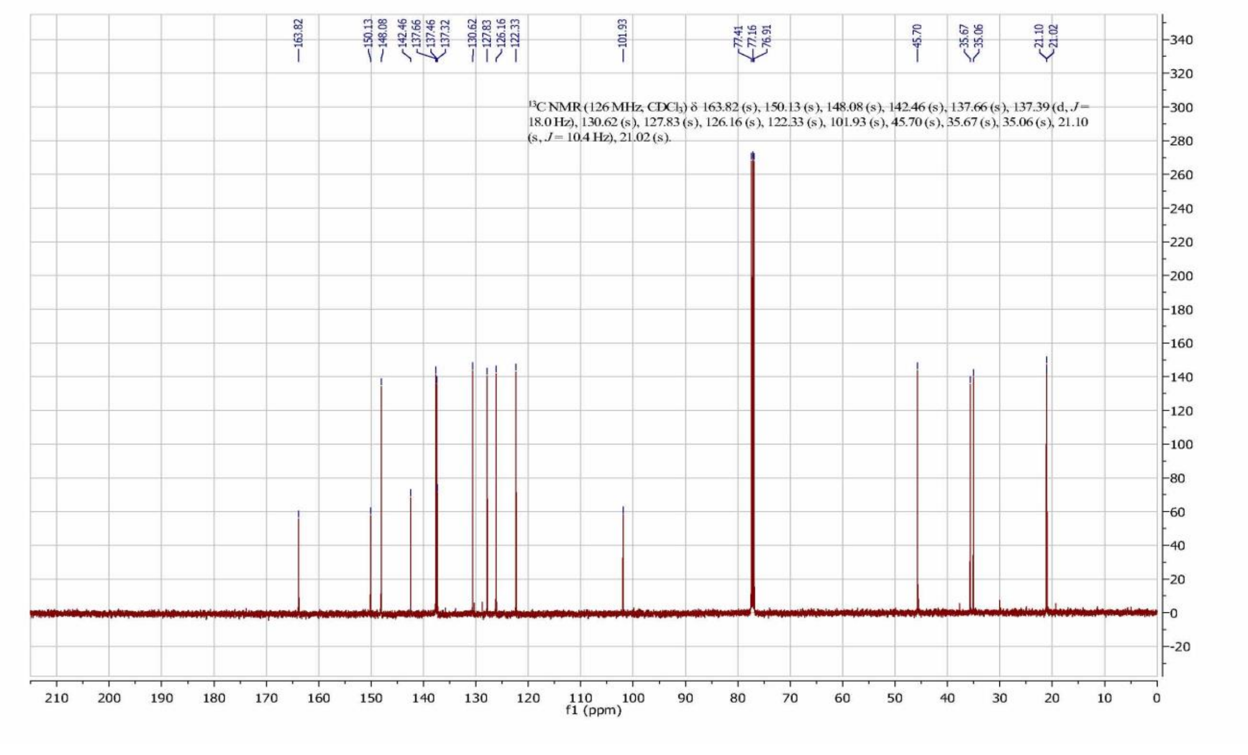
13C NMR (126 MHz, CDCl3) δ 163.82 (s), 150.13 (s), 148.08 (s), 142.46 (s), 137.66 (s), 137.39 (d, J = 18.0 Hz), 130.62 (s), 127.83 (s), 126.16 (s), 122.33 (s), 101.93 (s), 45.70 (s), 35.67 (s), 35.06 (s), 21.10 (s, J = 10.4 Hz), 21.02 (s).
f1 (ppm)

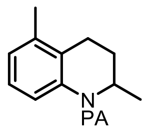
N
PA

11

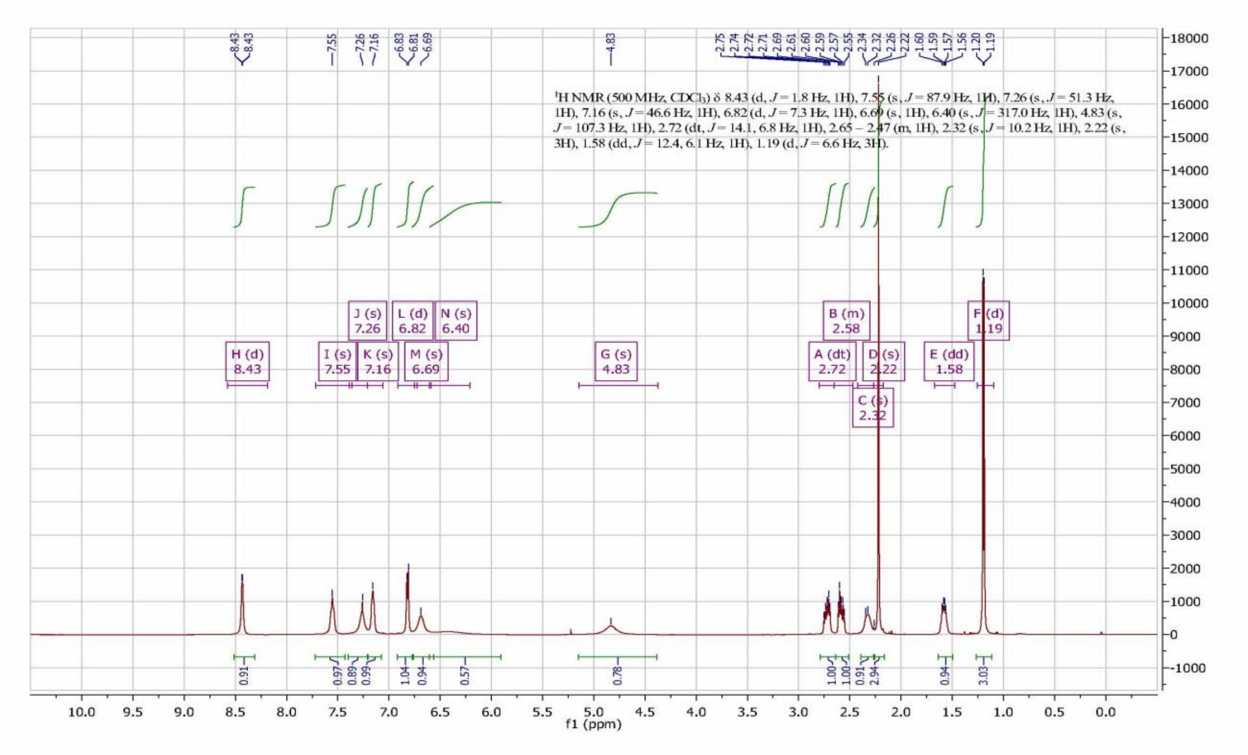
1H NMR (500 MHz, CDCl3) δ 8.43 (d, J = 1.8 Hz, 1H), 7.55 (s, J = 87.9 Hz, 1H), 7.26 (s, J = 51.3 Hz, 1H), 7.16 (s, J = 46.6 Hz, 1H), 6.82 (d, J = 7.3 Hz, 1H), 6.69 (s, 1H), 6.40 (s, J = 317.0 Hz, 1H), 4.83 (s, J = 107.3 Hz, 1H), 2.72 (dt, J = 14.1, 6.8 Hz, 1H), 2.65 – 2.47 (m, 1H), 2.32 (s, J = 10.2 Hz, 1H), 2.22 (s, 3H), 1.58 (dd, J = 12.4, 6.1 Hz, 1H), 1.19 (d, J = 6.6 Hz, 3H).
f1 (ppm)

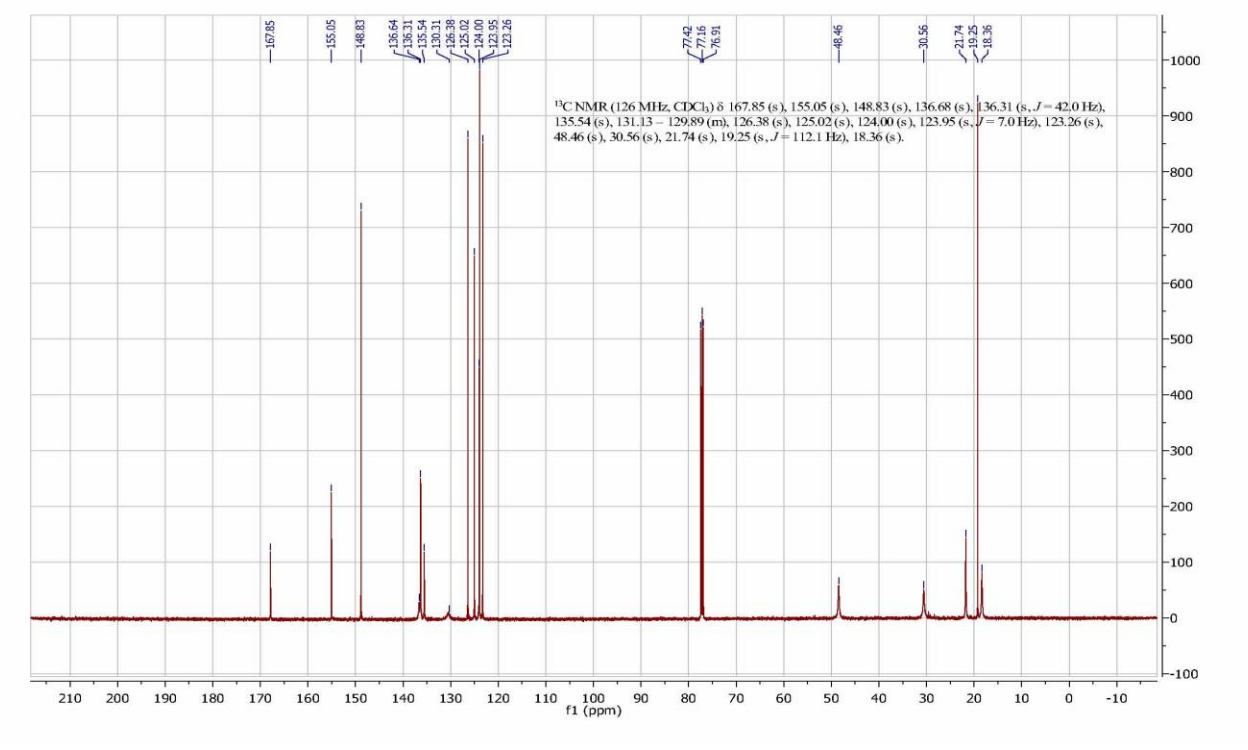
13C NMR (126 MHz, CDCl3) δ 167.85 (s), 155.05 (s), 148.83 (s), 136.68 (s), 136.31 (s, J = 42.0 Hz), 135.54 (s), 131.13 – 129.89 (m), 126.38 (s), 125.02 (s), 124.00 (s), 123.95 (s, J = 7.0 Hz), 123.26 (s), 48.46 (s), 30.56 (s), 21.74 (s), 19.25 (s, J = 112.1 Hz), 18.36 (s).
f1 (ppm)

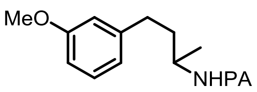
MeO
NHPA

12

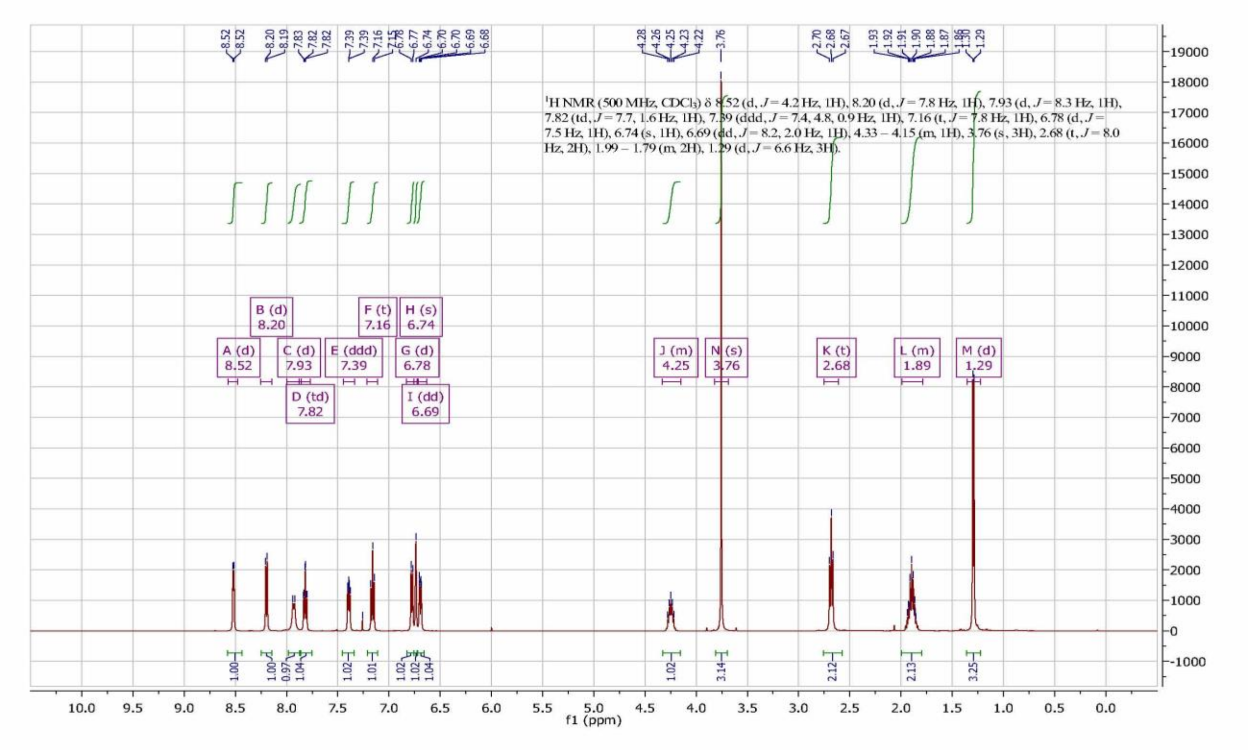
¹H NMR (500 MHz, CDCl₃) δ 8.52 (d, J = 4.2 Hz, 1H), 8.20 (d, J = 7.8 Hz, 1H), 7.93 (d, J = 8.3 Hz, 1H), 7.82 (td, J = 7.7, 1.6 Hz, 1H), 7.39 (ddd, J = 7.4, 4.8, 0.9 Hz, 1H), 7.16 (t, J = 7.8 Hz, 1H), 6.78 (d, J = 7.5 Hz, 1H), 6.74 (s, 1H), 6.69 (dd, J = 8.2, 2.0 Hz, 1H), 4.33 – 4.15 (m, 1H), 3.76 (s, 3H), 2.68 (t, J = 8.0 Hz, 2H), 1.99 – 1.79 (m, 2H), 1.29 (d, J = 6.6 Hz, 3H).
A (d) 8.52
B (d) 8.20
C (d) 7.93
D (td) 7.82
E (ddd) 7.39
F (t) 7.16
G (d) 6.78
H (s) 6.74
I (dd) 6.69
J (m) 4.25
N (s) 3.76
K (t) 2.68
L (m) 1.89
M (d) 1.29
f1 (ppm)

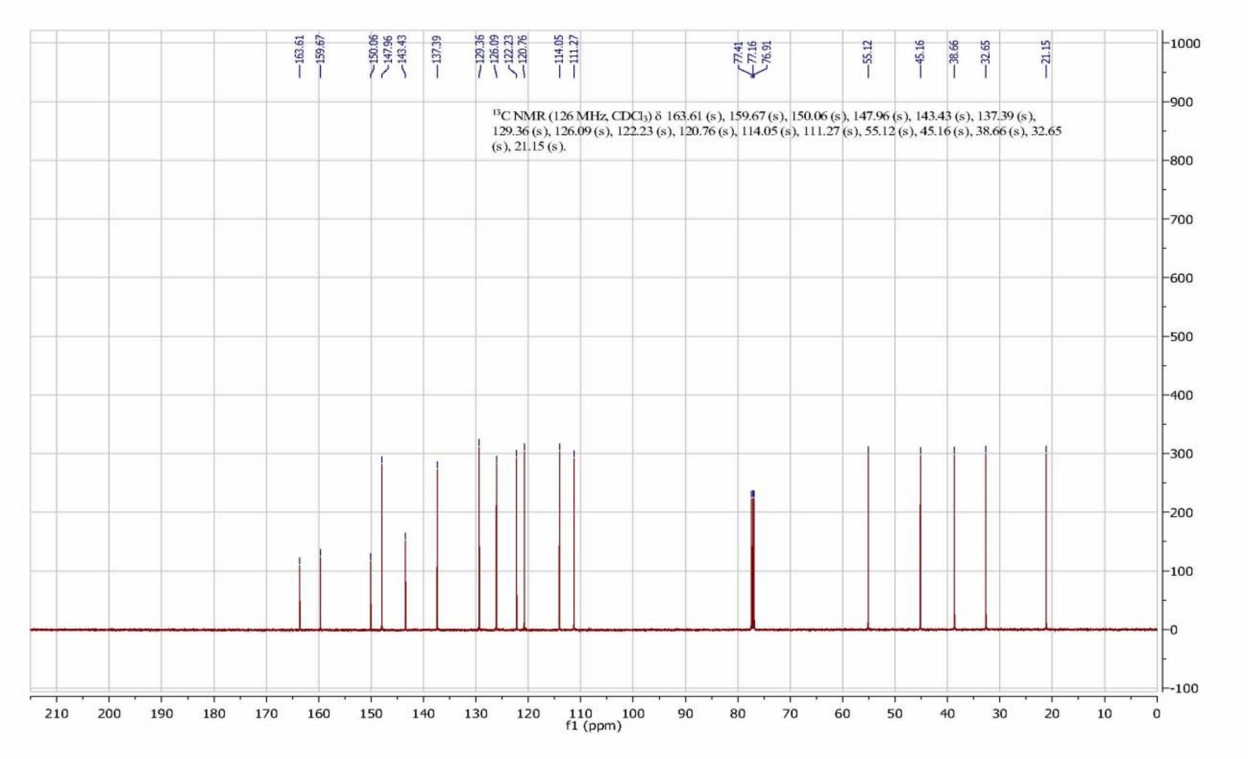
¹³C NMR (126 MHz, CDCl₃) δ 163.61 (s), 159.67 (s), 150.06 (s), 147.96 (s), 143.43 (s), 137.39 (s), 129.36 (s), 126.09 (s), 122.23 (s), 120.76 (s), 114.05 (s), 111.27 (s), 55.12 (s), 45.16 (s), 38.66 (s), 32.65 (s), 21.15 (s).
f1 (ppm)

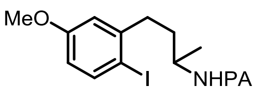
MeO
I
NHPA

13

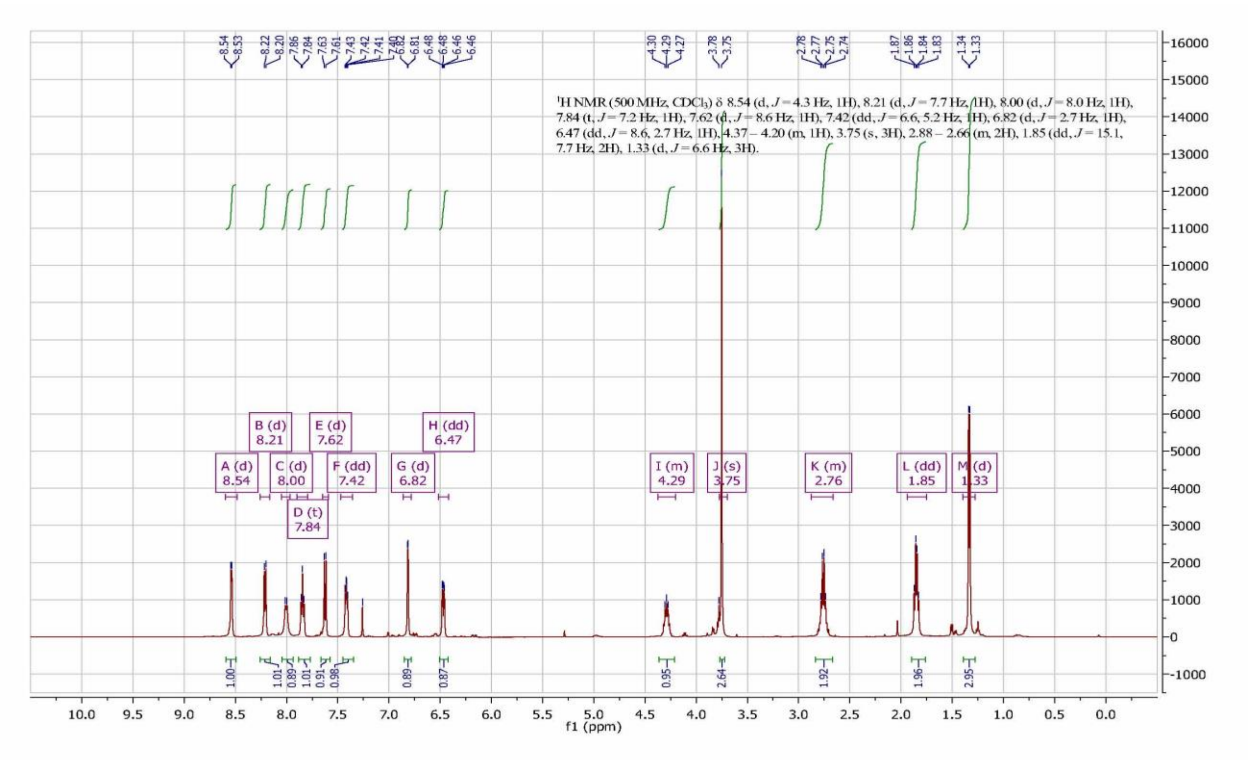
¹H NMR (500 MHz, CDCl₃) δ 8.54 (d, J = 4.3 Hz, 1H), 8.21 (d, J = 7.7 Hz, 1H), 8.00 (d, J = 8.0 Hz, 1H), 7.84 (t, J = 7.2 Hz, 1H), 7.62 (d, J = 8.6 Hz, 1H), 7.42 (dd, J = 6.6, 5.2 Hz, 1H), 6.82 (d, J = 2.7 Hz, 1H), 6.47 (dd, J = 8.6, 2.7 Hz, 1H), 4.37 – 4.20 (m, 1H), 3.75 (s, 3H), 2.88 – 2.66 (m, 2H), 1.85 (dd, J = 15.1, 7.7 Hz, 2H), 1.33 (d, J = 6.6 Hz, 3H).
f1 (ppm)

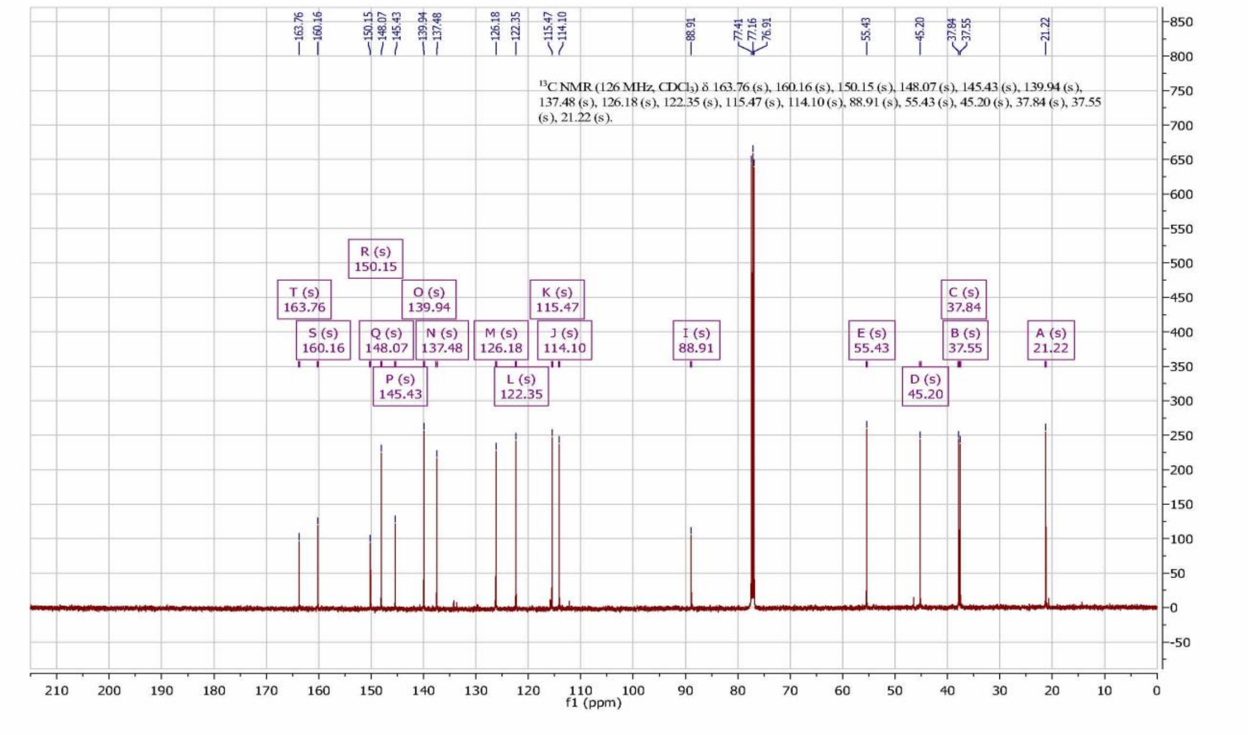
¹³C NMR (126 MHz, CDCl₃) δ 163.76 (s), 160.16 (s), 150.15 (s), 148.07 (s), 145.43 (s), 139.94 (s), 137.48 (s), 126.18 (s), 122.35 (s), 115.47 (s), 114.10 (s), 88.91 (s), 55.43 (s), 45.20 (s), 37.84 (s), 37.55 (s), 21.22 (s).
f1 (ppm)

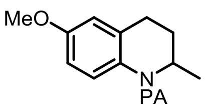
MeO
N
PA

14

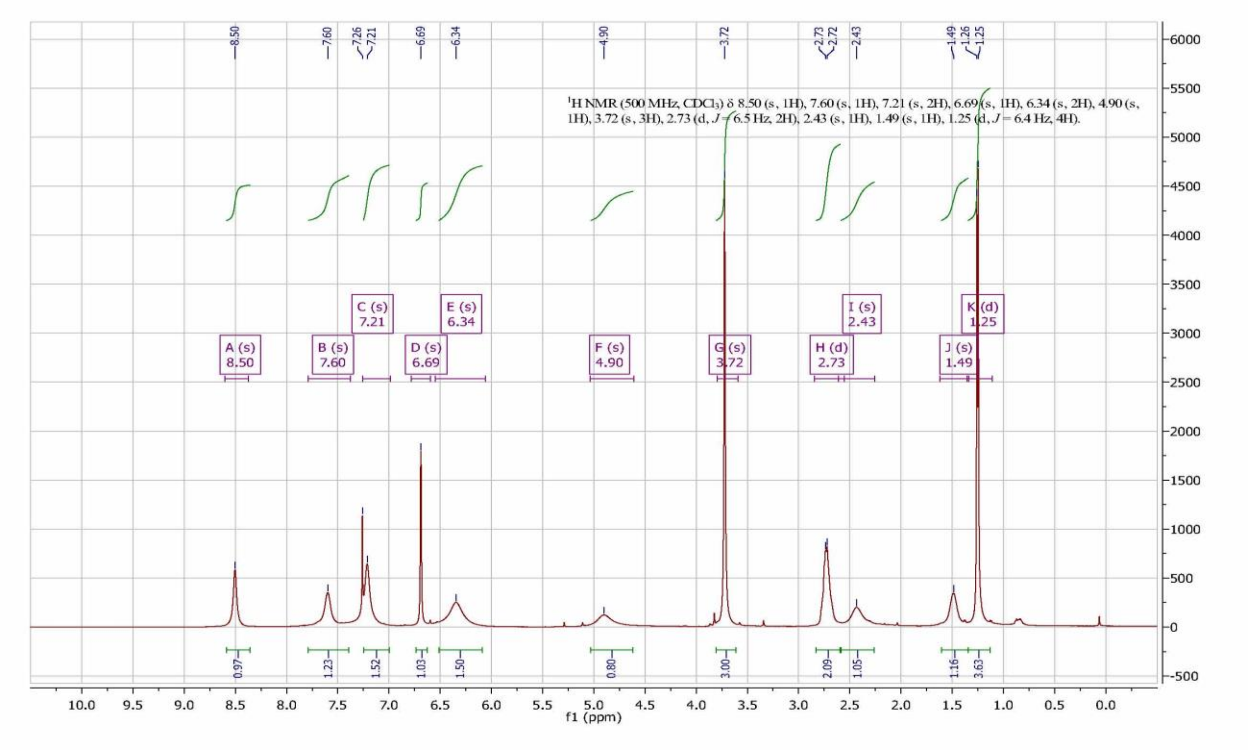
^{1}H NMR (500 MHz, CDCl$_3$) δ 8.50 (s, 1H), 7.60 (s, 1H), 7.21 (s, 2H), 6.69 (s, 1H), 6.34 (s, 2H), 4.90 (s, 1H), 3.72 (s, 3H), 2.73 (d, J = 6.5 Hz, 2H), 2.43 (s, 1H), 1.49 (s, 1H), 1.25 (d, J = 6.4 Hz, 4H).
f1 (ppm)

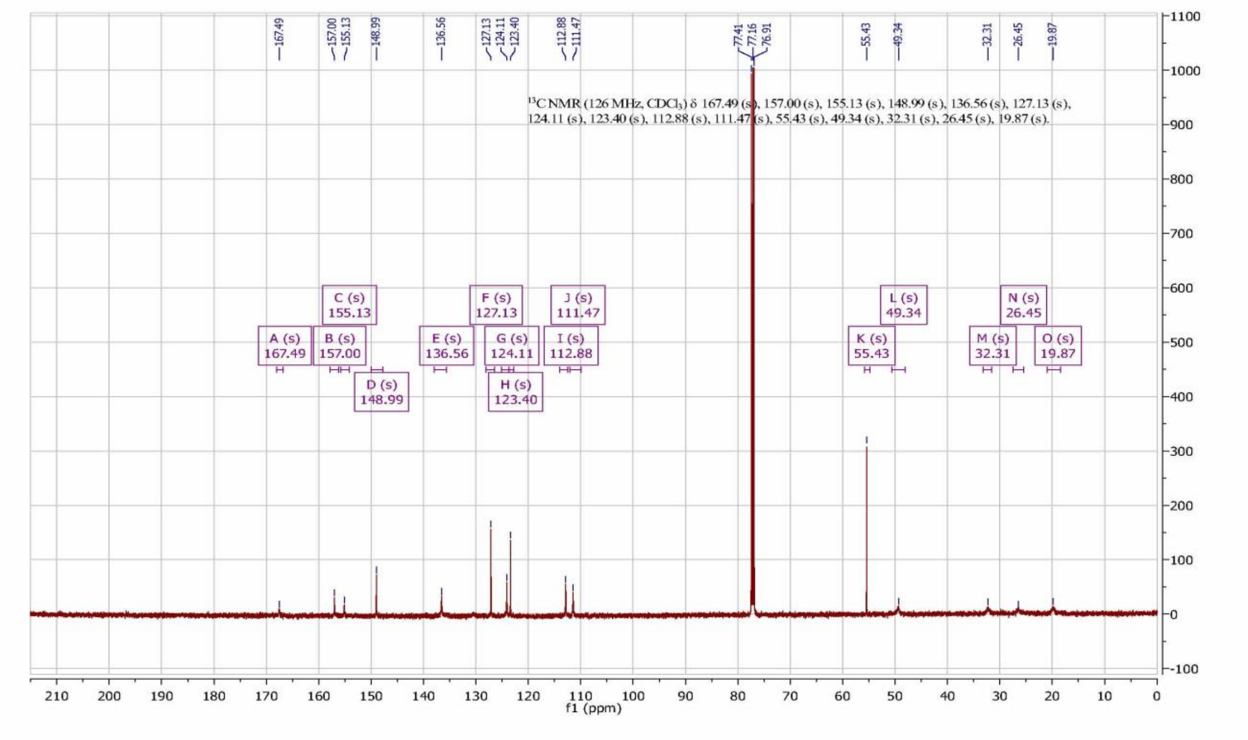
^{13}C NMR (126 MHz, CDCl$_3$) δ 167.49 (s), 157.00 (s), 155.13 (s), 148.99 (s), 136.56 (s), 127.13 (s), 124.11 (s), 123.40 (s), 112.88 (s), 111.47 (s), 55.43 (s), 49.34 (s), 32.31 (s), 26.45 (s), 19.87 (s).
f1 (ppm)

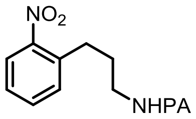

NO_2
NHPA

15

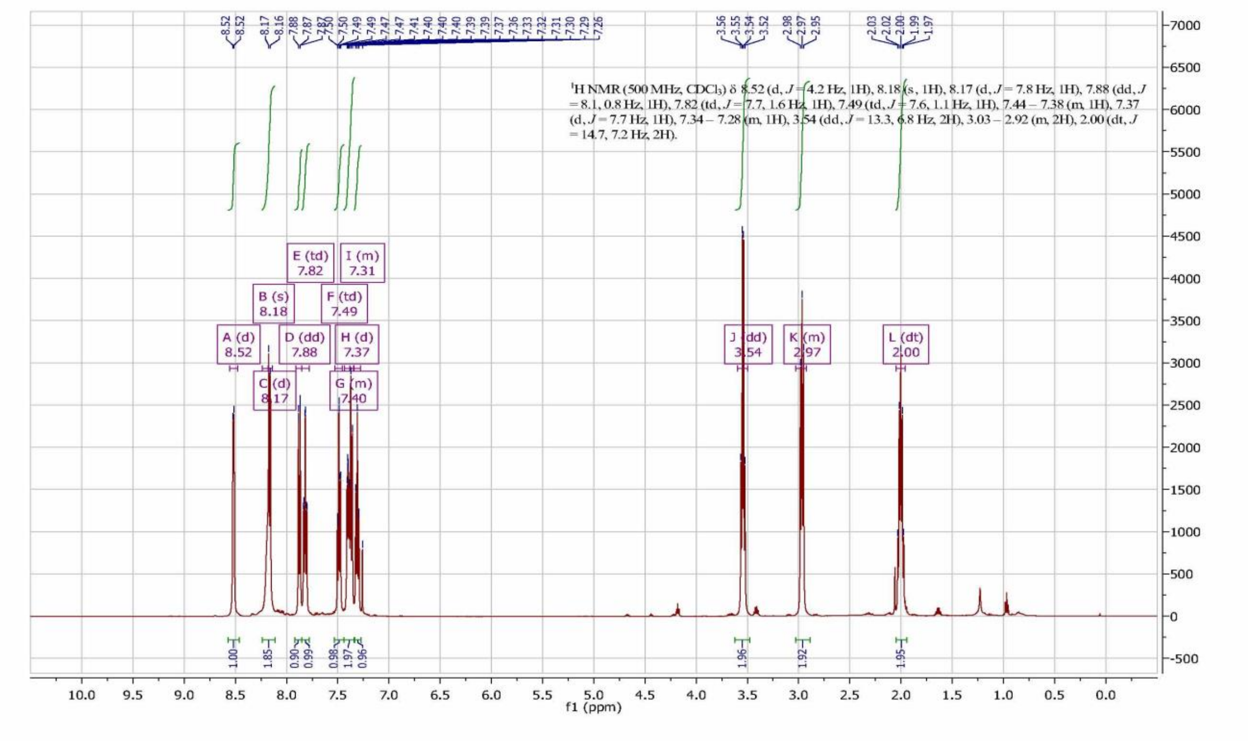

¹H NMR (500 MHz, CDCl₃) δ 8.52 (d, J = 4.2 Hz, 1H), 8.18 (s, 1H), 8.17 (d, J = 7.8 Hz, 1H), 7.88 (dd, J = 8.1, 0.8 Hz, 1H), 7.82 (td, J = 7.7, 1.6 Hz, 1H), 7.49 (td, J = 7.6, 1.1 Hz, 1H), 7.44 – 7.38 (m, 1H), 7.37 (d, J = 7.7 Hz, 1H), 7.34 – 7.28 (m, 1H), 3.54 (dd, J = 13.3, 6.8 Hz, 2H), 3.03 – 2.92 (m, 2H), 2.00 (dt, J = 14.7, 7.2 Hz, 2H).
A (d) 8.52
B (s) 8.18
C (d) 8.17
D (dd) 7.88
E (td) 7.82
F (td) 7.49
G (m) 7.40
H (d) 7.37
I (m) 7.31
J (dd) 3.54
K (m) 2.97
L (dt) 2.00
f1 (ppm)

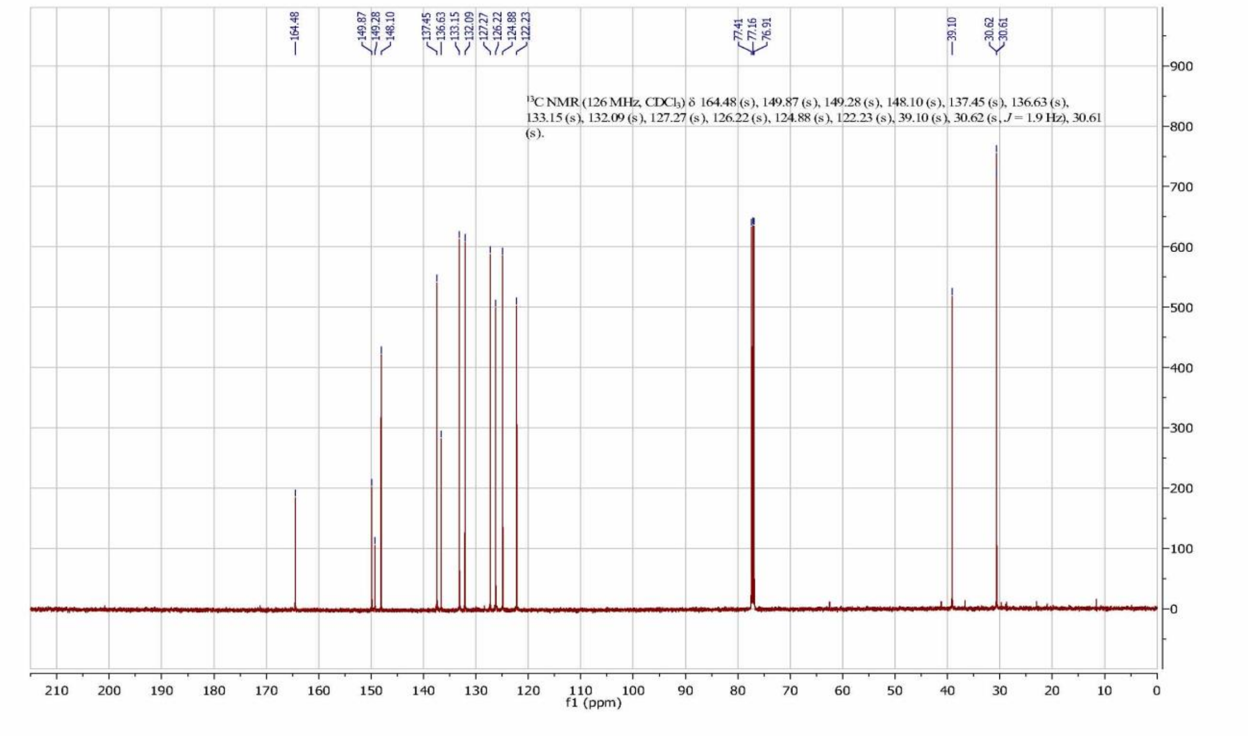

¹³C NMR (126 MHz, CDCl₃) δ 164.48 (s), 149.87 (s), 149.28 (s), 148.10 (s), 137.45 (s), 136.63 (s), 133.15 (s), 132.09 (s), 127.27 (s), 126.22 (s), 124.88 (s), 122.23 (s), 39.10 (s), 30.62 (s, J = 1.9 Hz), 30.61 (s).
f1 (ppm)

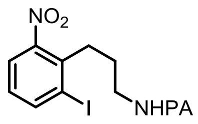
NO2
I
NHPA

16

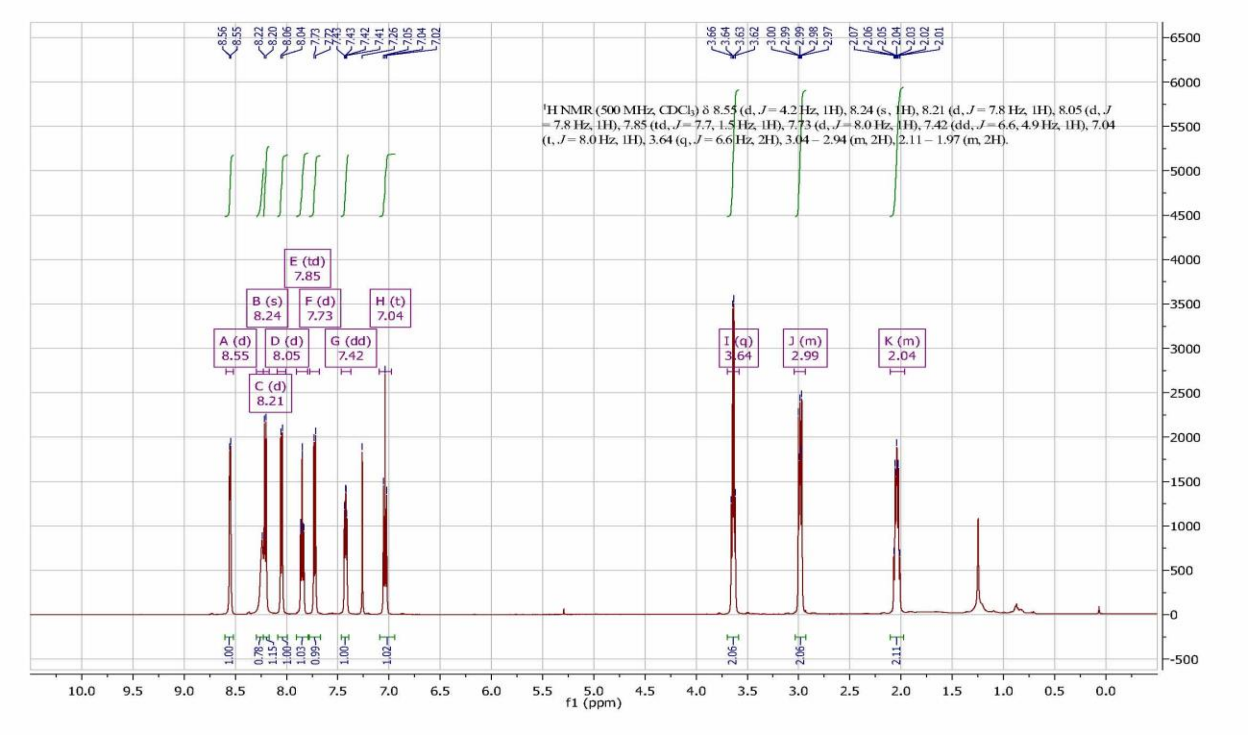
¹H NMR (500 MHz, CDCl₃) δ 8.55 (d, J = 4.2 Hz, 1H), 8.24 (s, 1H), 8.21 (d, J = 7.8 Hz, 1H), 8.05 (d, J = 7.8 Hz, 1H), 7.85 (td, J = 7.7, 1.5 Hz, 1H), 7.73 (d, J = 8.0 Hz, 1H), 7.42 (dd, J = 6.6, 4.9 Hz, 1H), 7.04 (t, J = 8.0 Hz, 1H), 3.64 (q, J = 6.6 Hz, 2H), 3.04 – 2.94 (m, 2H), 2.11 – 1.97 (m, 2H).
A (d) 8.55
B (s) 8.24
C (d) 8.21
D (d) 8.05
E (td) 7.85
F (d) 7.73
G (dd) 7.42
H (t) 7.04
I (q) 3.64
J (m) 2.99
K (m) 2.04
f1 (ppm)

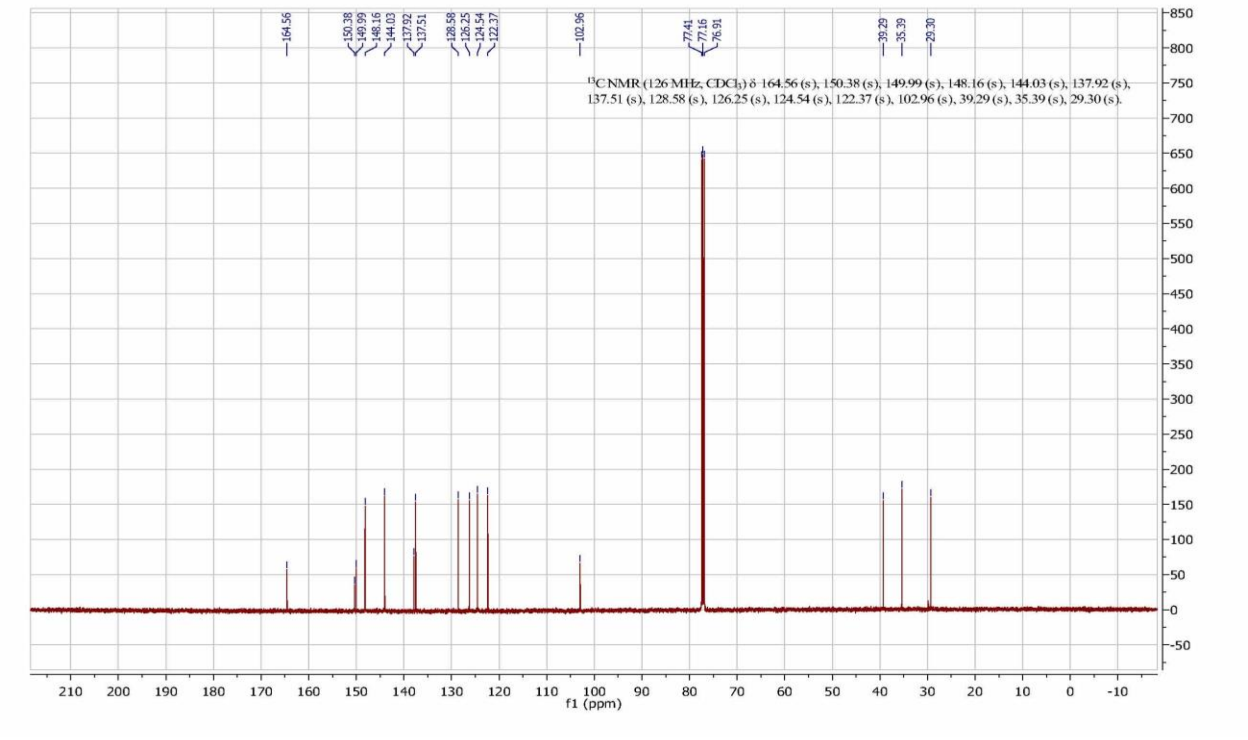
¹³C NMR (126 MHz, CDCl₃) δ 164.56 (s), 150.38 (s), 149.99 (s), 148.16 (s), 144.03 (s), 137.92 (s), 137.51 (s), 128.58 (s), 126.25 (s), 124.54 (s), 122.37 (s), 102.96 (s), 39.29 (s), 35.39 (s), 29.30 (s).
f1 (ppm)

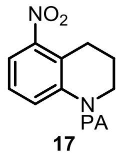
NO2
N
PA

17

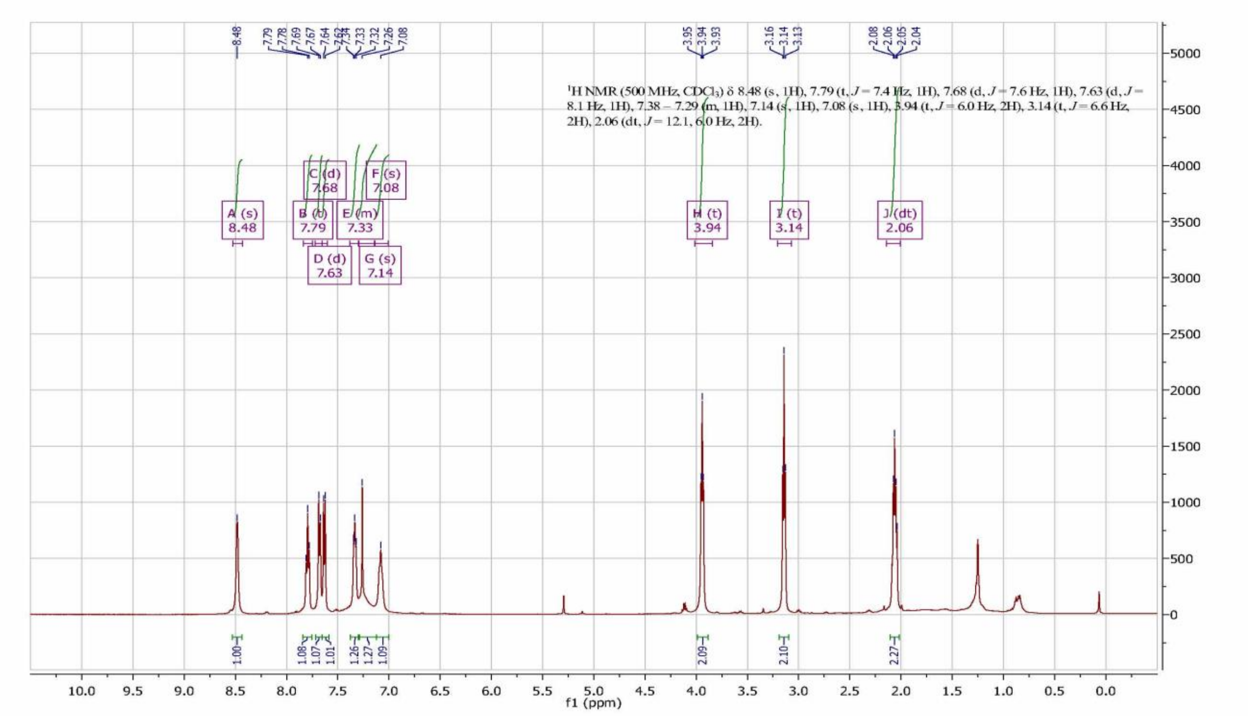
1H NMR (500 MHz, CDCl3) δ 8.48 (s, 1H), 7.79 (t, J = 7.4 Hz, 1H), 7.68 (d, J = 7.6 Hz, 1H), 7.63 (d, J = 8.1 Hz, 1H), 7.38 – 7.29 (m, 1H), 7.14 (s, 1H), 7.08 (s, 1H), 3.94 (t, J = 6.0 Hz, 2H), 3.14 (t, J = 6.6 Hz, 2H), 2.06 (dt, J = 12.1, 6.0 Hz, 2H).
f1 (ppm)

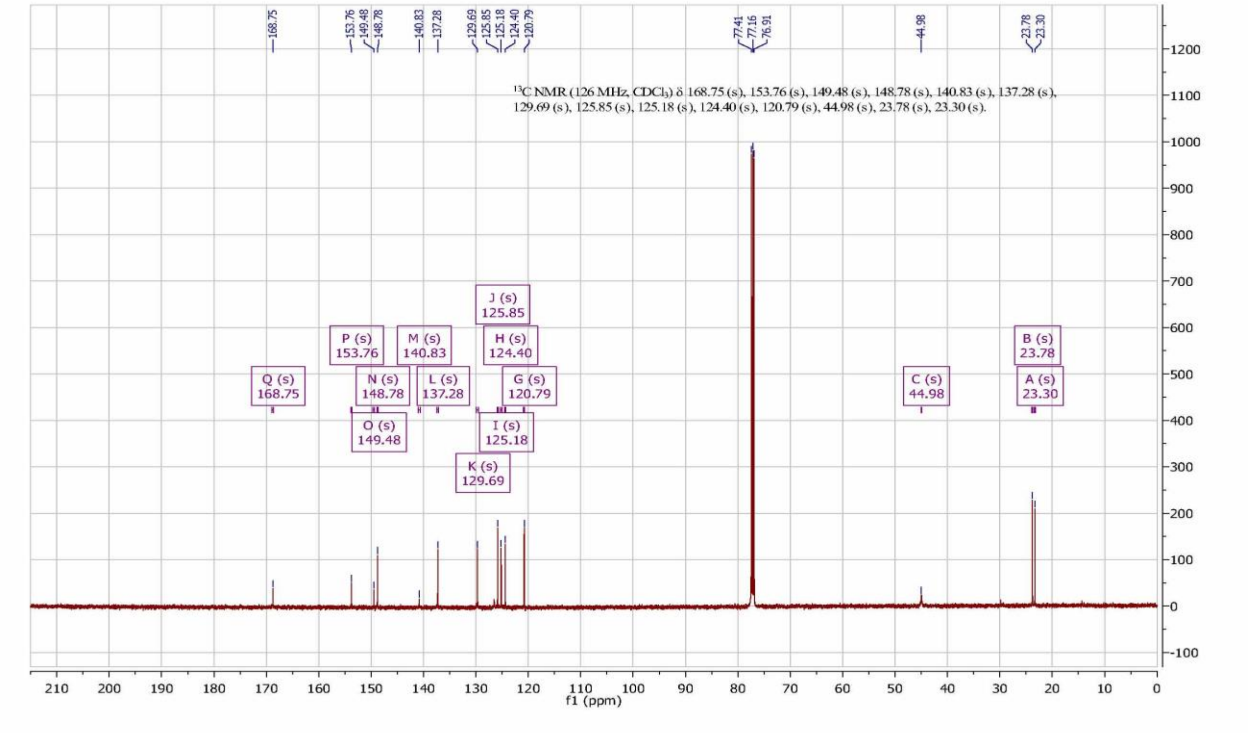
13C NMR (126 MHz, CDCl3) δ 168.75 (s), 153.76 (s), 149.48 (s), 148.78 (s), 140.83 (s), 137.28 (s), 129.69 (s), 125.85 (s), 125.18 (s), 124.40 (s), 120.79 (s), 44.98 (s), 23.78 (s), 23.30 (s).
f1 (ppm)

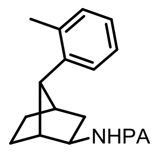

18

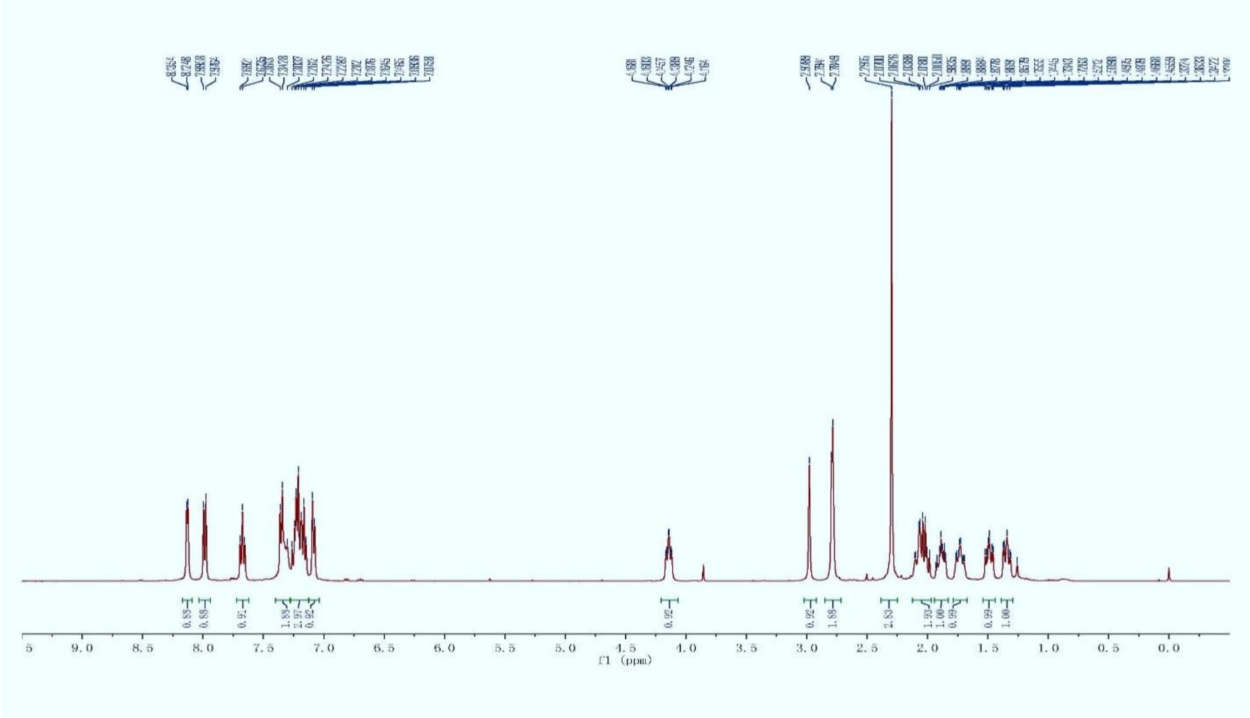

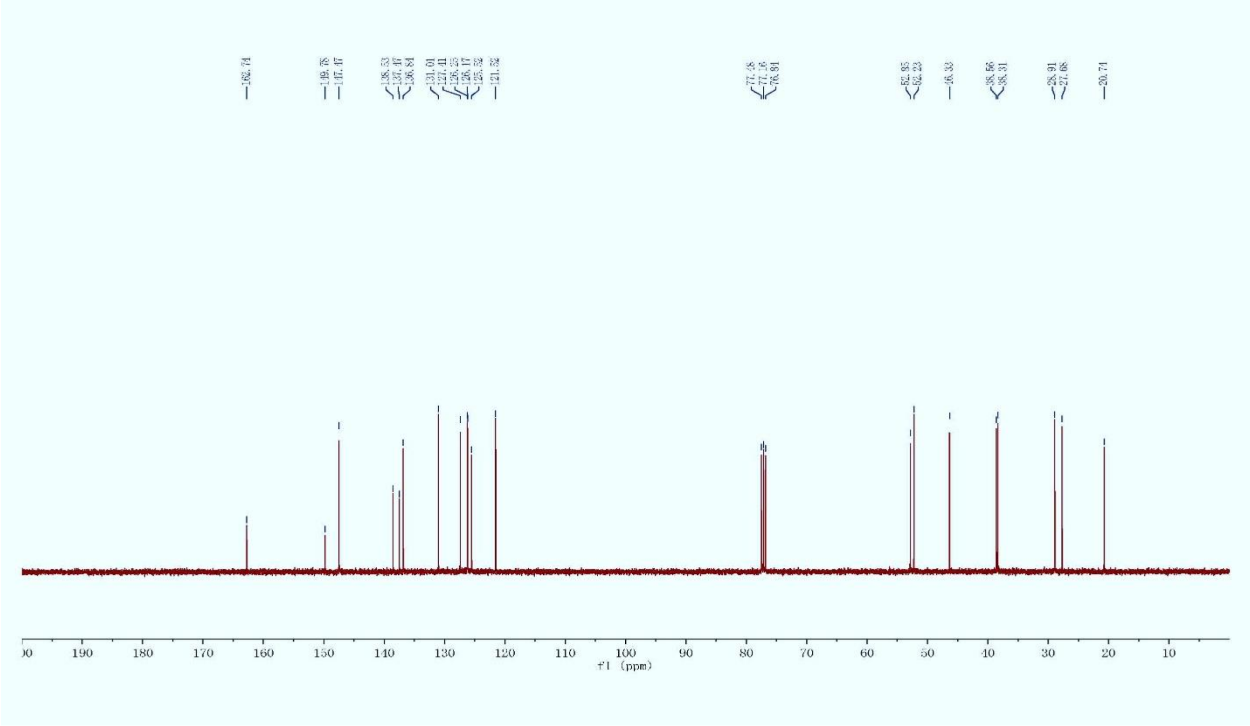

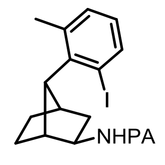
I
NHPA

19

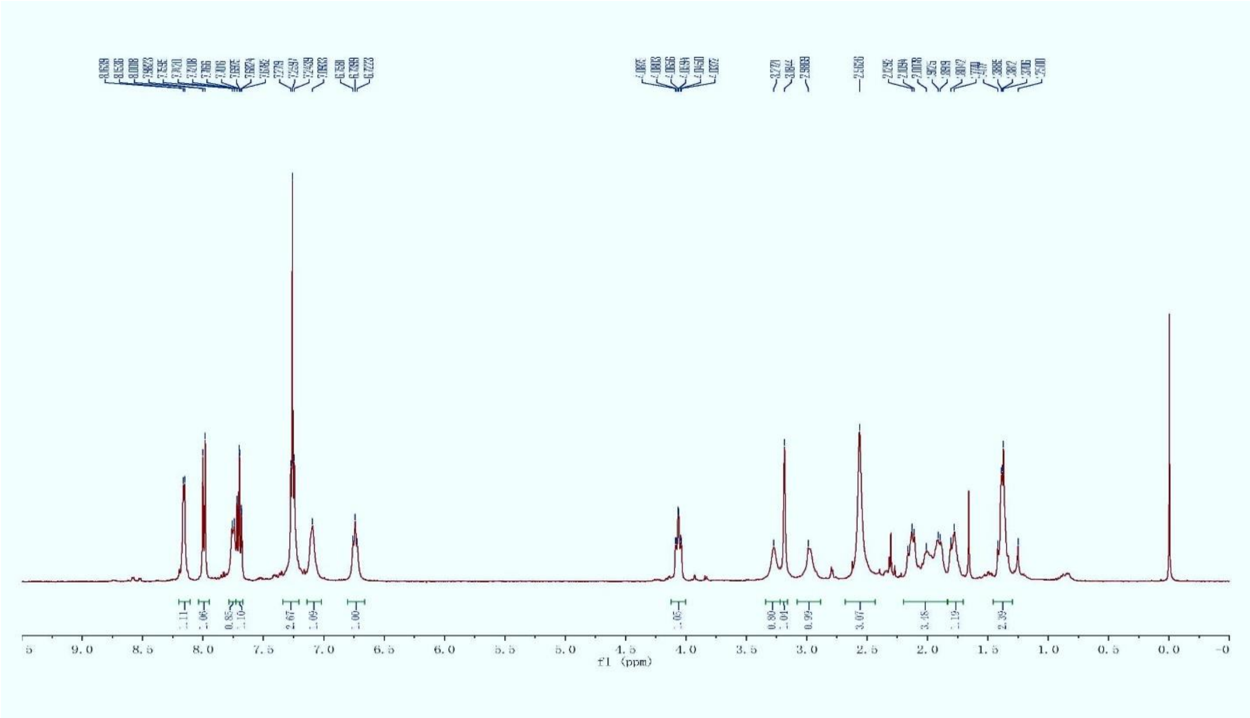
f1 (ppm)

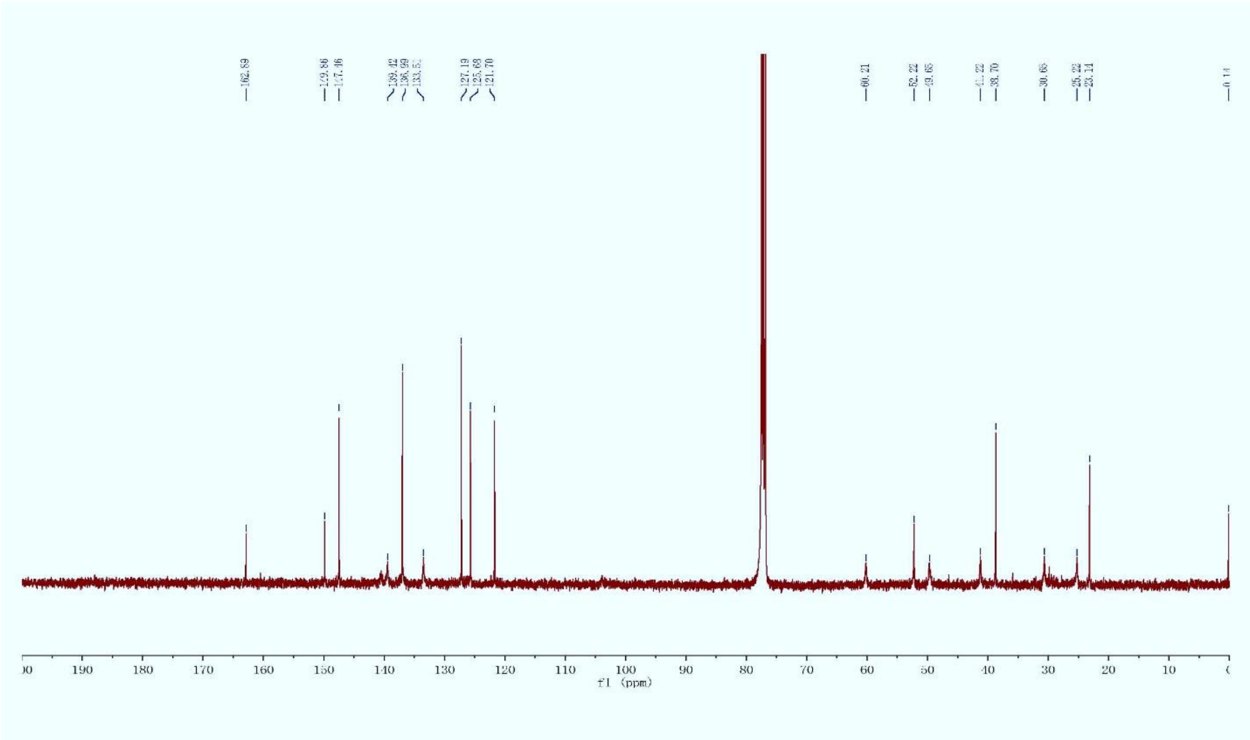
162.89
149.86
147.46
139.42
136.59
133.55
127.19
125.68
121.70
60.21
52.22
49.65
41.22
38.70
30.66
25.22
23.14
f1 (ppm)

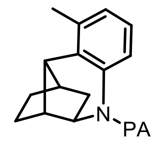
N
PA

20

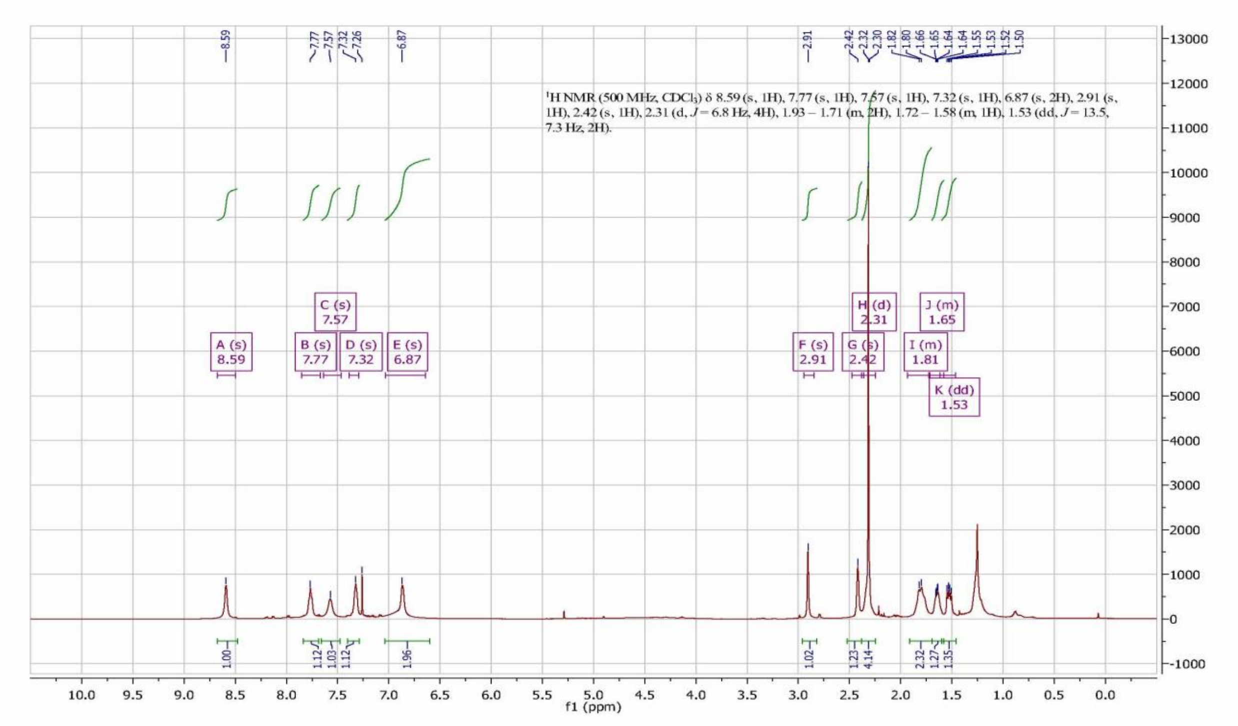
^{1}H NMR (500 MHz, $CDCl_3$) δ 8.59 (s, 1H), 7.77 (s, 1H), 7.57 (s, 1H), 7.32 (s, 1H), 6.87 (s, 2H), 2.91 (s, 1H), 2.42 (s, 1H), 2.31 (d, J = 6.8 Hz, 4H), 1.93 – 1.71 (m, 2H), 1.72 – 1.58 (m, 1H), 1.53 (dd, J = 13.5, 7.3 Hz, 2H).
f1 (ppm)

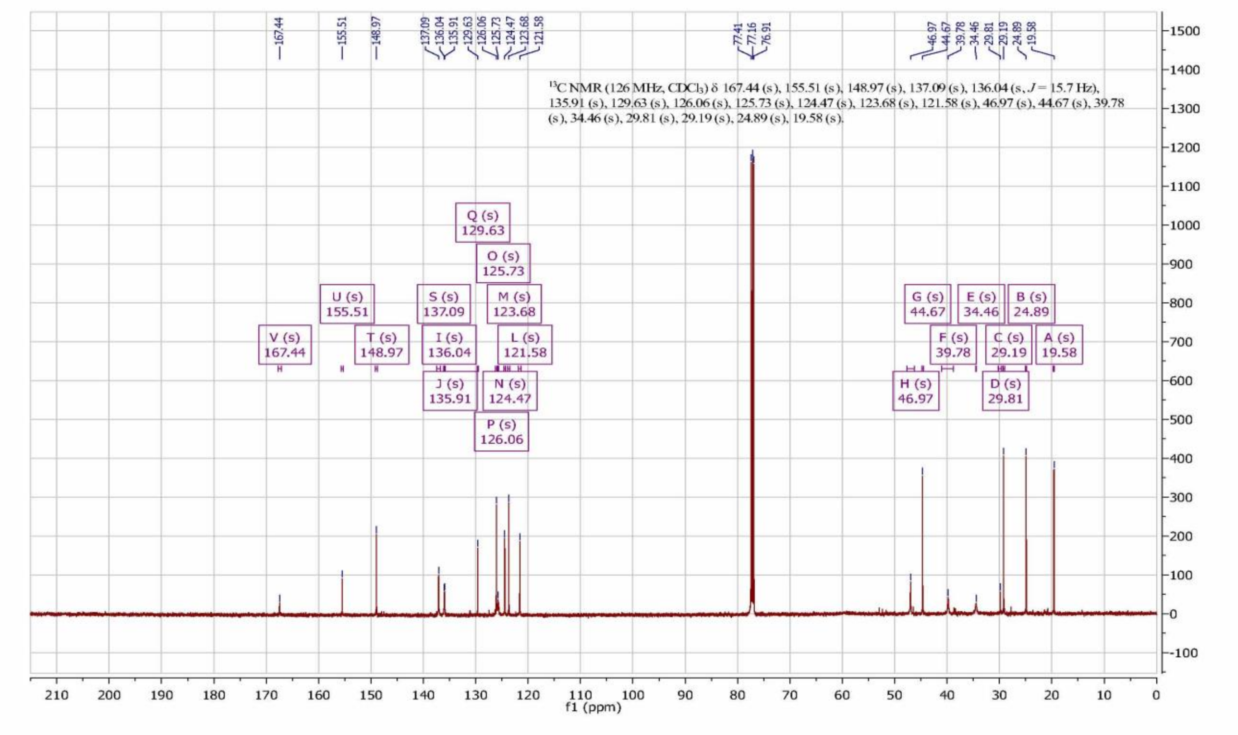
^{13}C NMR (126 MHz, $CDCl_3$) δ 167.44 (s), 155.51 (s), 148.97 (s), 137.09 (s), 136.04 (s, J = 15.7 Hz), 135.91 (s), 129.63 (s), 126.06 (s), 125.73 (s), 124.47 (s), 123.68 (s), 121.58 (s), 46.97 (s), 44.67 (s), 39.78 (s), 34.46 (s), 29.81 (s), 29.19 (s), 24.89 (s), 19.58 (s).
f1 (ppm)

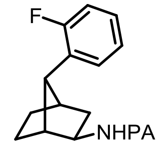
F
NHPA

21

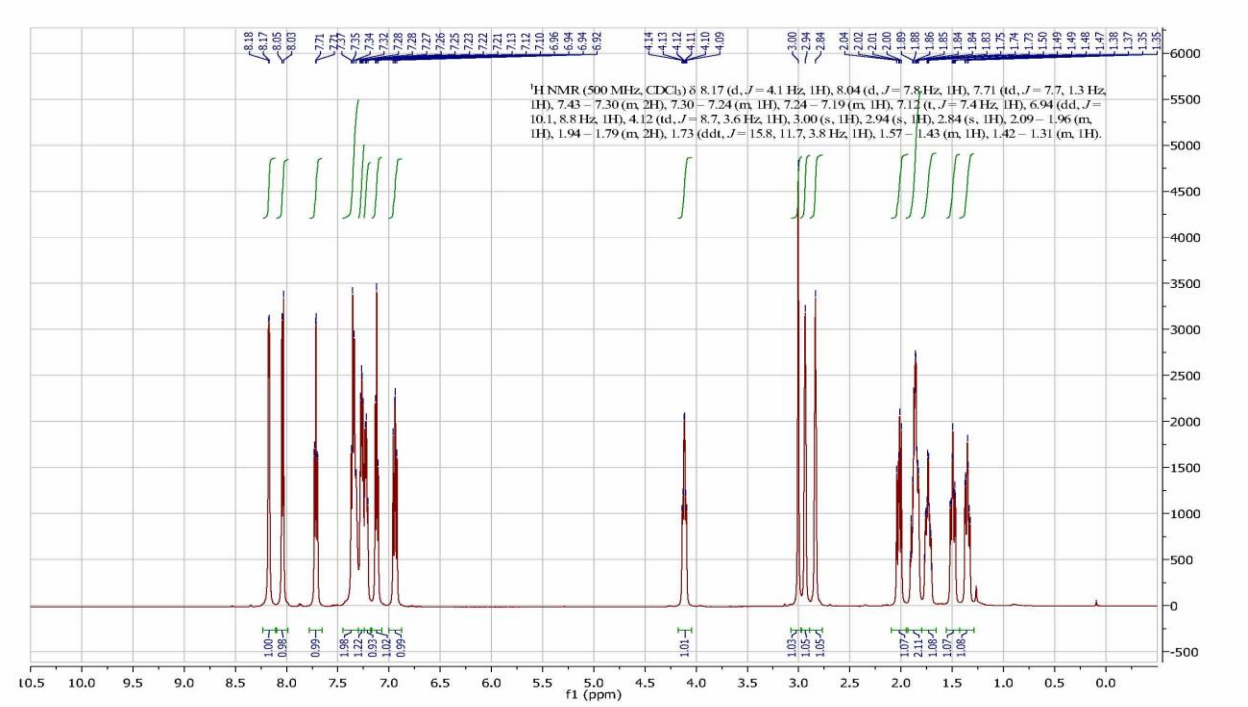
^{1}H NMR (500 MHz, CDCl$_3$) δ 8.17 (d, J = 4.1 Hz, 1H), 8.04 (d, J = 7.8 Hz, 1H), 7.71 (td, J = 7.7, 1.3 Hz, 1H), 7.43 – 7.30 (m, 2H), 7.30 – 7.24 (m, 1H), 7.24 – 7.19 (m, 1H), 7.12 (t, J = 7.4 Hz, 1H), 6.94 (dd, J = 10.1, 8.8 Hz, 1H), 4.12 (td, J = 8.7, 3.6 Hz, 1H), 3.00 (s, 1H), 2.94 (s, 1H), 2.84 (s, 1H), 2.09 – 1.96 (m, 1H), 1.94 – 1.79 (m, 2H), 1.73 (ddt, J = 15.8, 11.7, 3.8 Hz, 1H), 1.57 – 1.43 (m, 1H), 1.42 – 1.31 (m, 1H).
f1 (ppm)

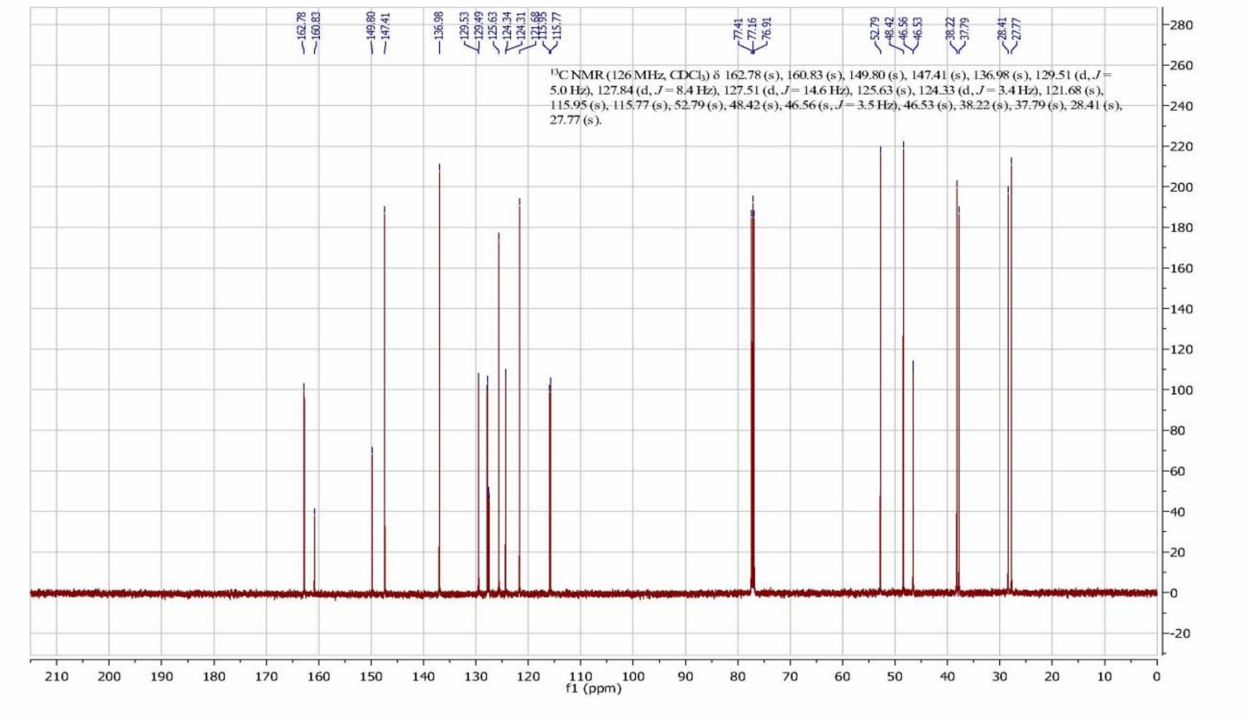
^{13}C NMR (126 MHz, CDCl$_3$) δ 162.78 (s), 160.83 (s), 149.80 (s), 147.41 (s), 136.98 (s), 129.51 (d, J = 5.0 Hz), 127.84 (d, J = 8.4 Hz), 127.51 (d, J = 14.6 Hz), 125.63 (s), 124.33 (d, J = 3.4 Hz), 121.68 (s), 115.95 (s), 115.77 (s), 52.79 (s), 48.42 (s), 46.56 (s, J = 3.5 Hz), 46.53 (s), 38.22 (s), 37.79 (s), 28.41 (s), 27.77 (s).
f1 (ppm)

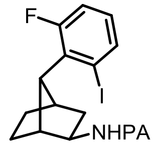

22

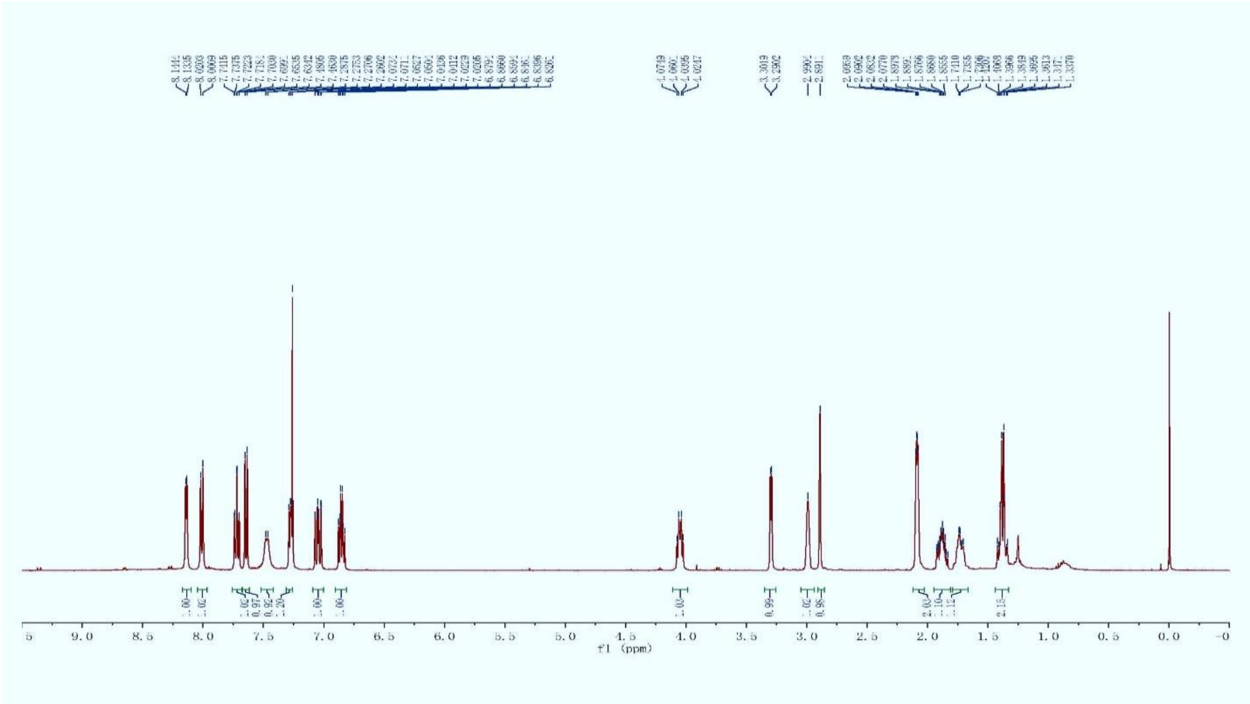

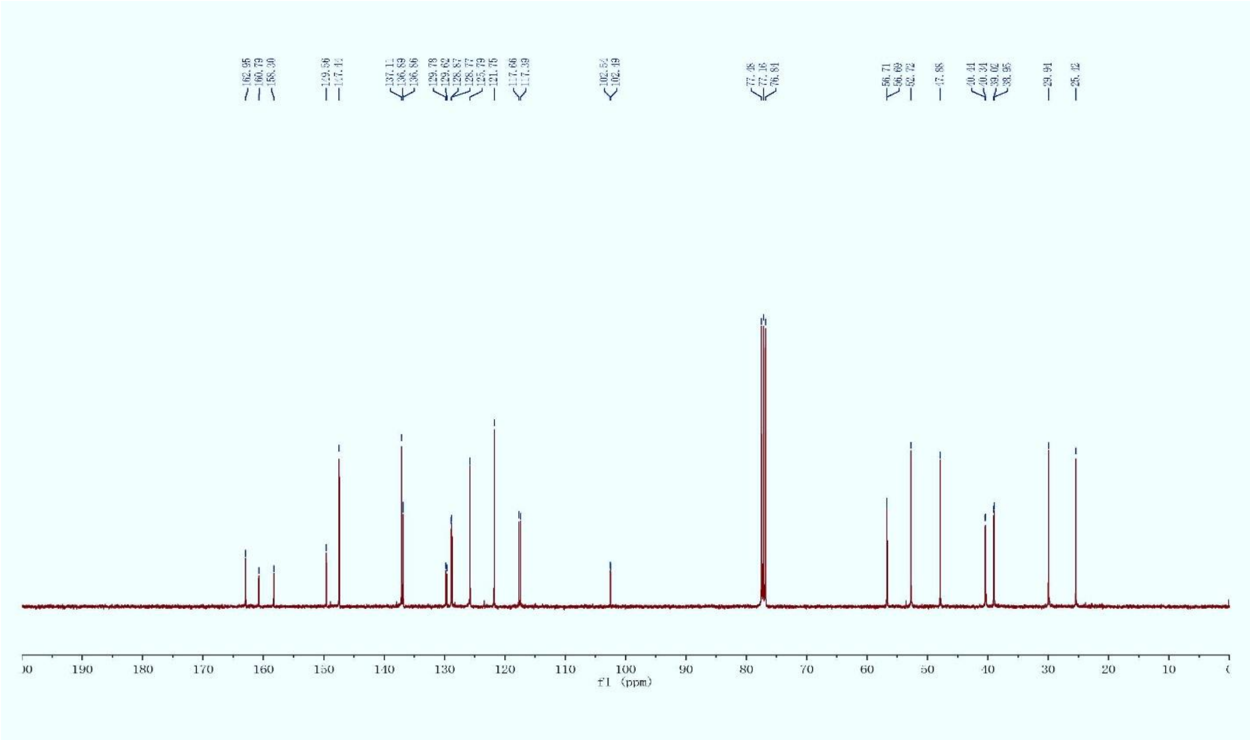

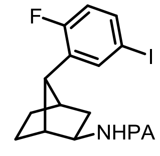
F
I
NHPA

23

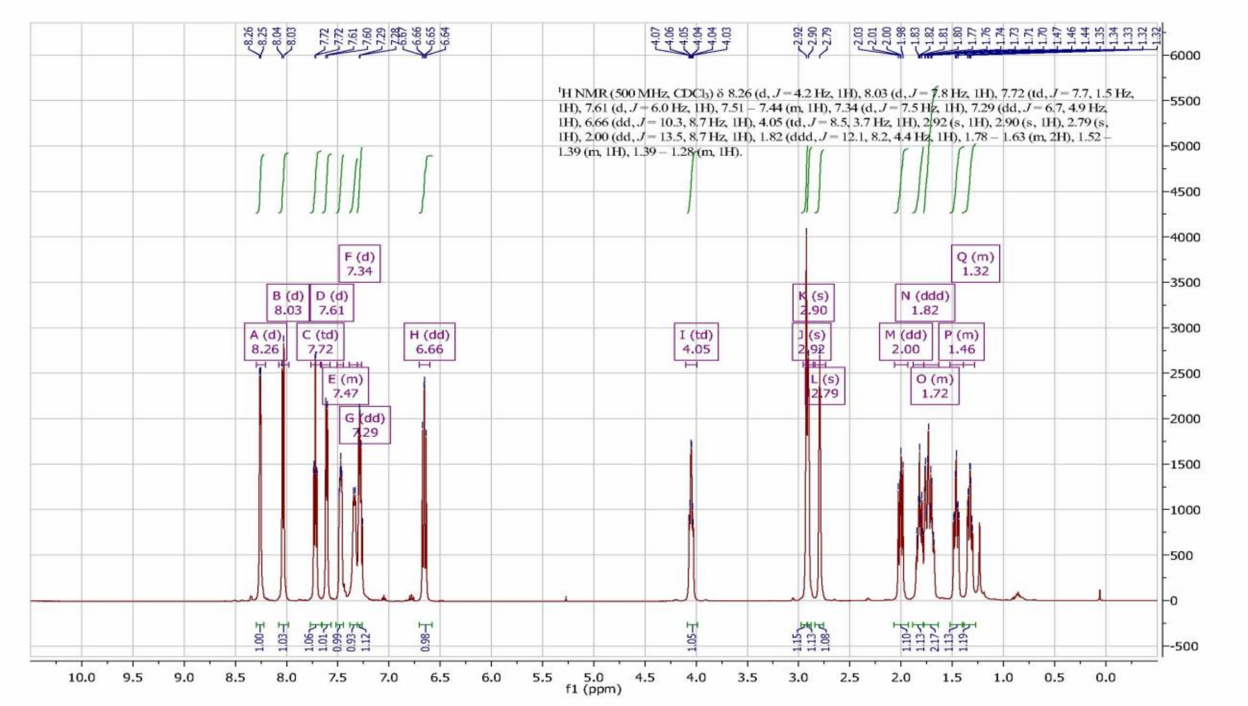
¹H NMR (500 MHz, CDCl₃) δ 8.26 (d, J = 4.2 Hz, 1H), 8.03 (d, J = 7.8 Hz, 1H), 7.72 (td, J = 7.7, 1.5 Hz, 1H), 7.61 (d, J = 6.0 Hz, 1H), 7.51 – 7.44 (m, 1H), 7.34 (d, J = 7.5 Hz, 1H), 7.29 (dd, J = 6.7, 4.9 Hz, 1H), 6.66 (dd, J = 10.3, 8.7 Hz, 1H), 4.05 (td, J = 8.5, 3.7 Hz, 1H), 2.92 (s, 1H), 2.90 (s, 1H), 2.79 (s, 1H), 2.00 (dd, J = 13.5, 8.7 Hz, 1H), 1.82 (ddd, J = 12.1, 8.2, 4.4 Hz, 1H), 1.78 – 1.63 (m, 2H), 1.52 – 1.39 (m, 1H), 1.39 – 1.28 (m, 1H).
f1 (ppm)

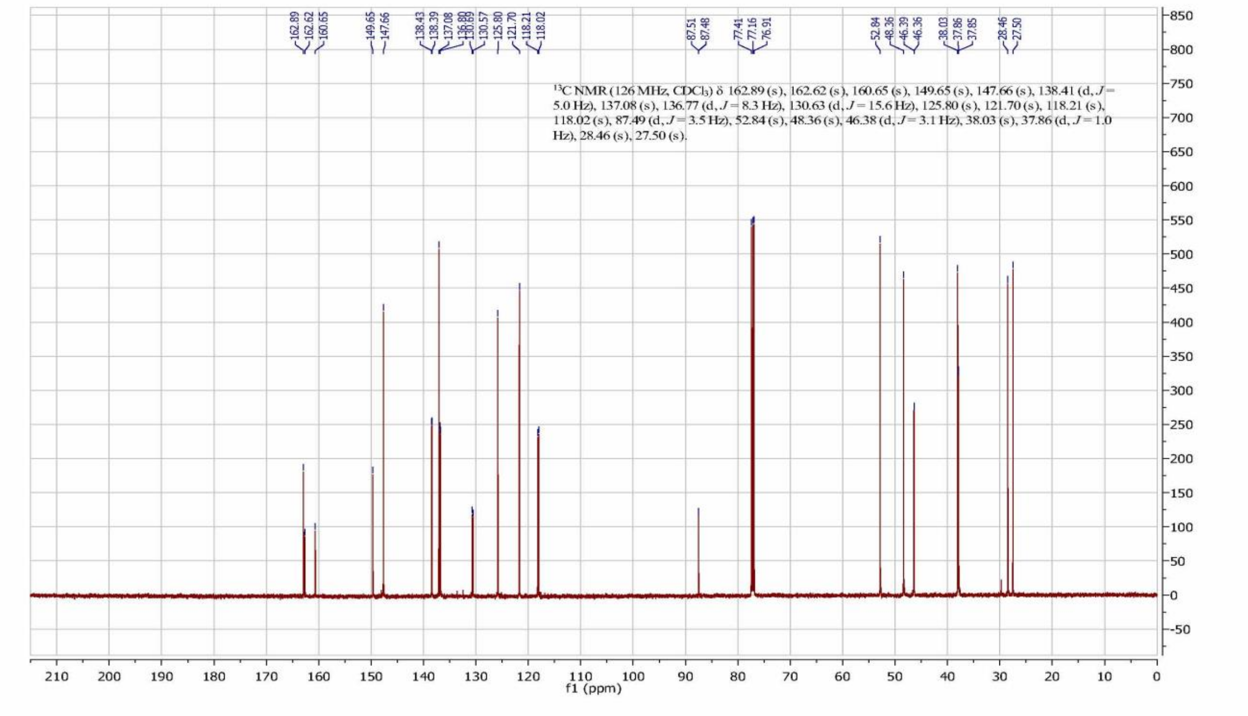
¹³C NMR (126 MHz, CDCl₃) δ 162.89 (s), 162.62 (s), 160.65 (s), 149.65 (s), 147.66 (s), 138.41 (d, J = 5.0 Hz), 137.08 (s), 136.77 (d, J = 8.3 Hz), 130.63 (d, J = 15.6 Hz), 125.80 (s), 121.70 (s), 118.21 (s), 118.02 (s), 87.49 (d, J = 3.5 Hz), 52.84 (s), 48.36 (s), 46.38 (d, J = 3.1 Hz), 38.03 (s), 37.86 (d, J = 1.0 Hz), 28.46 (s), 27.50 (s).
f1 (ppm)

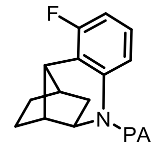

24

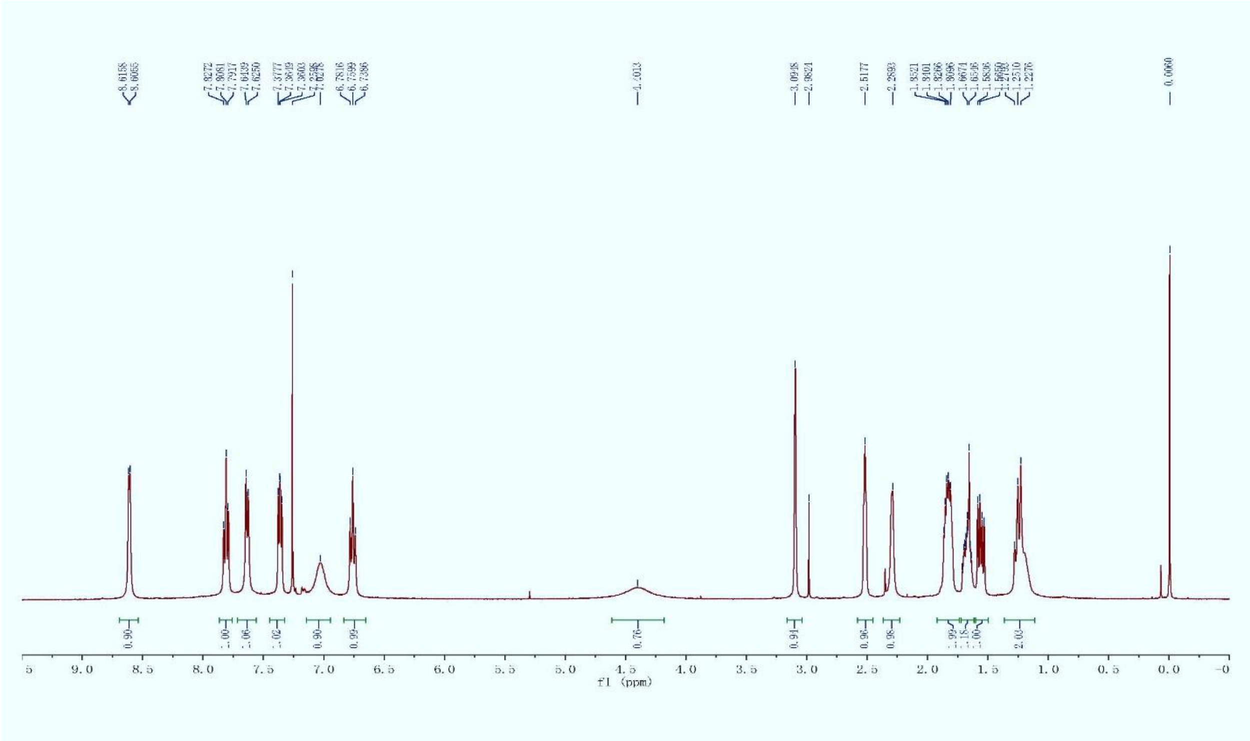

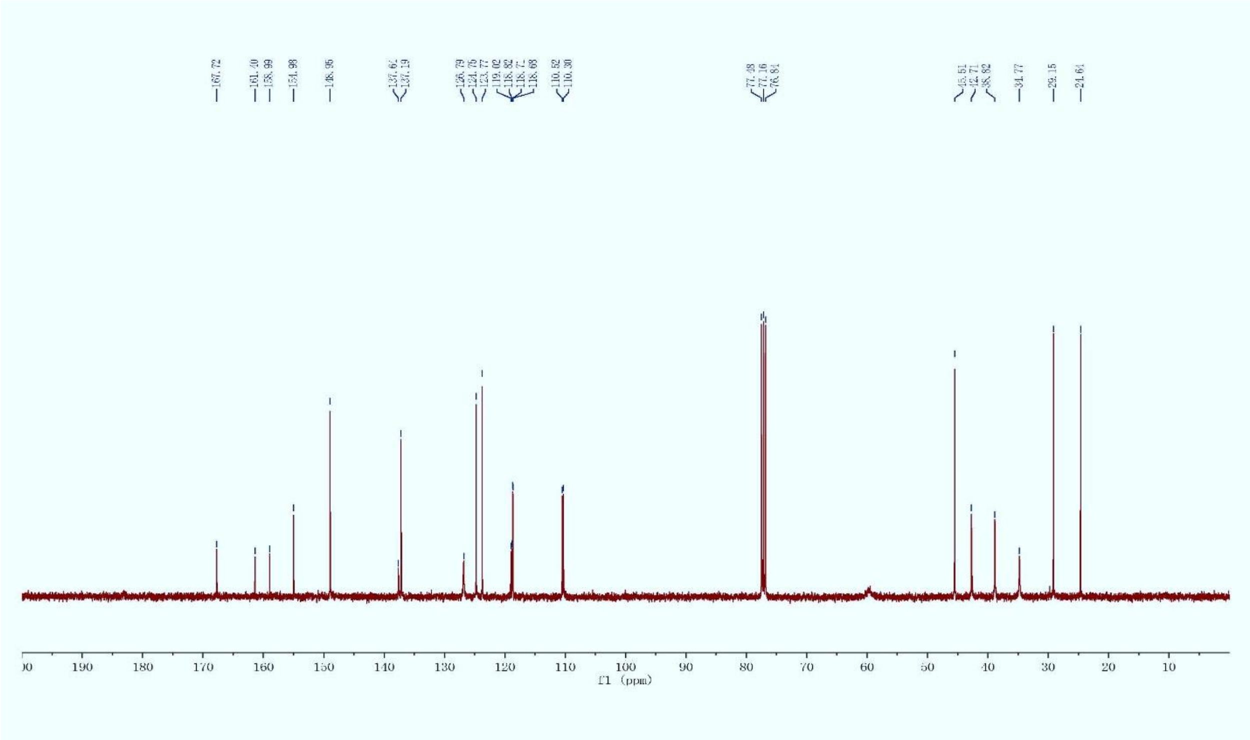

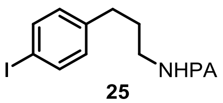
I
NHPA

25

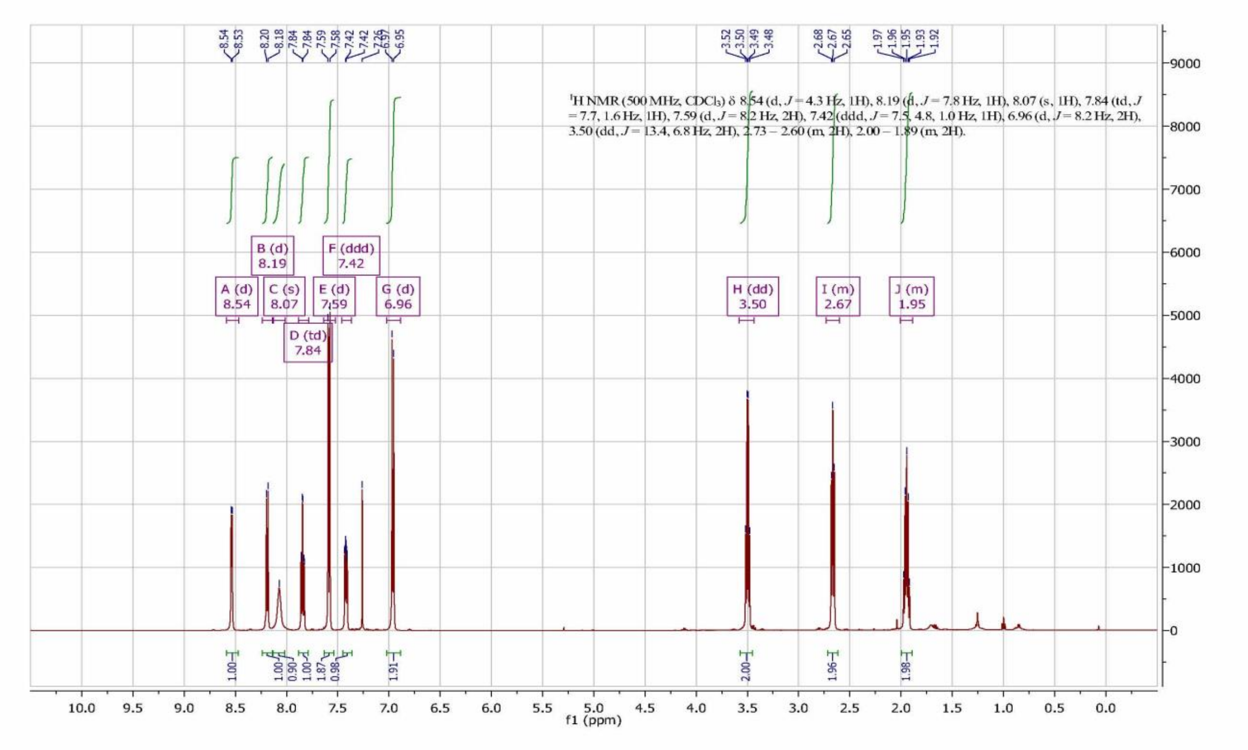
^{1}H NMR (500 MHz, CDCl3) δ 8.54 (d, J = 4.3 Hz, 1H), 8.19 (d, J = 7.8 Hz, 1H), 8.07 (s, 1H), 7.84 (td, J = 7.7, 1.6 Hz, 1H), 7.59 (d, J = 8.2 Hz, 2H), 7.42 (ddd, J = 7.5, 4.8, 1.0 Hz, 1H), 6.96 (d, J = 8.2 Hz, 2H), 3.50 (dd, J = 13.4, 6.8 Hz, 2H), 2.73 – 2.60 (m, 2H), 2.00 – 1.89 (m, 2H).
f1 (ppm)

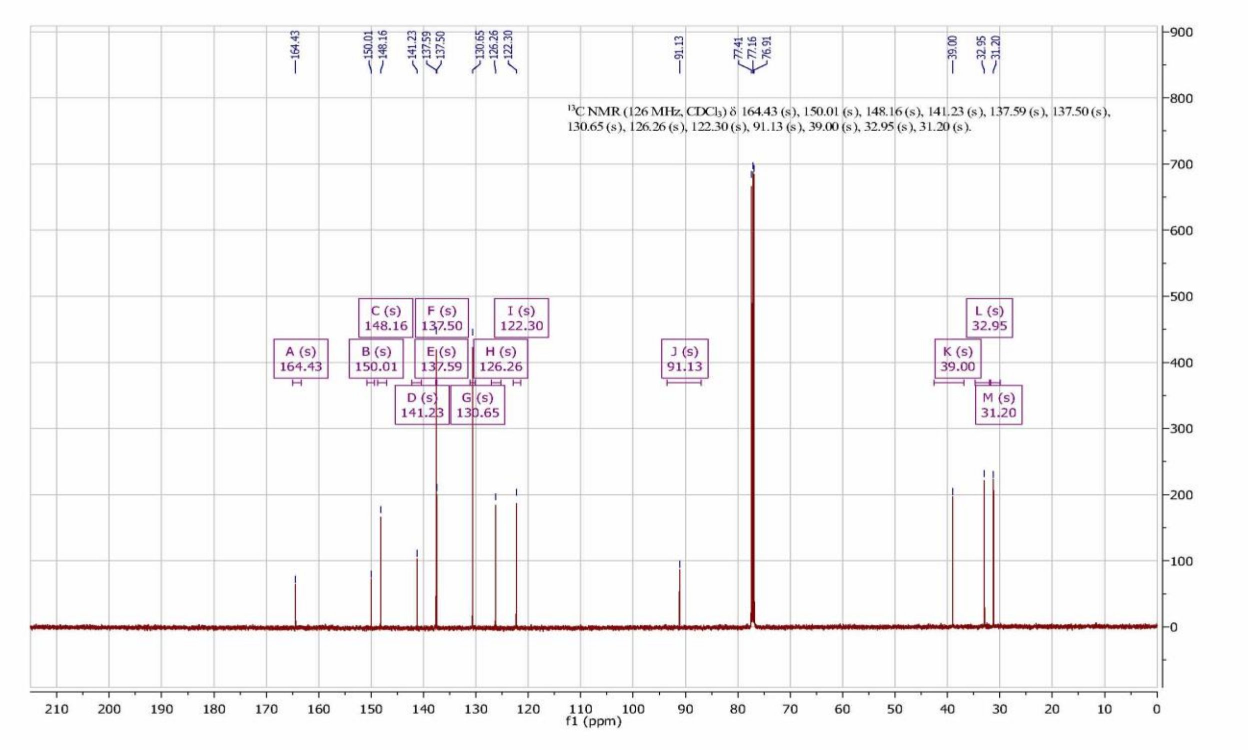
^{13}C NMR (126 MHz, CDCl3) δ 164.43 (s), 150.01 (s), 148.16 (s), 141.23 (s), 137.59 (s), 137.50 (s), 130.65 (s), 126.26 (s), 122.30 (s), 91.13 (s), 39.00 (s), 32.95 (s), 31.20 (s).
f1 (ppm)

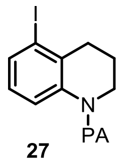
I
N
PA

27

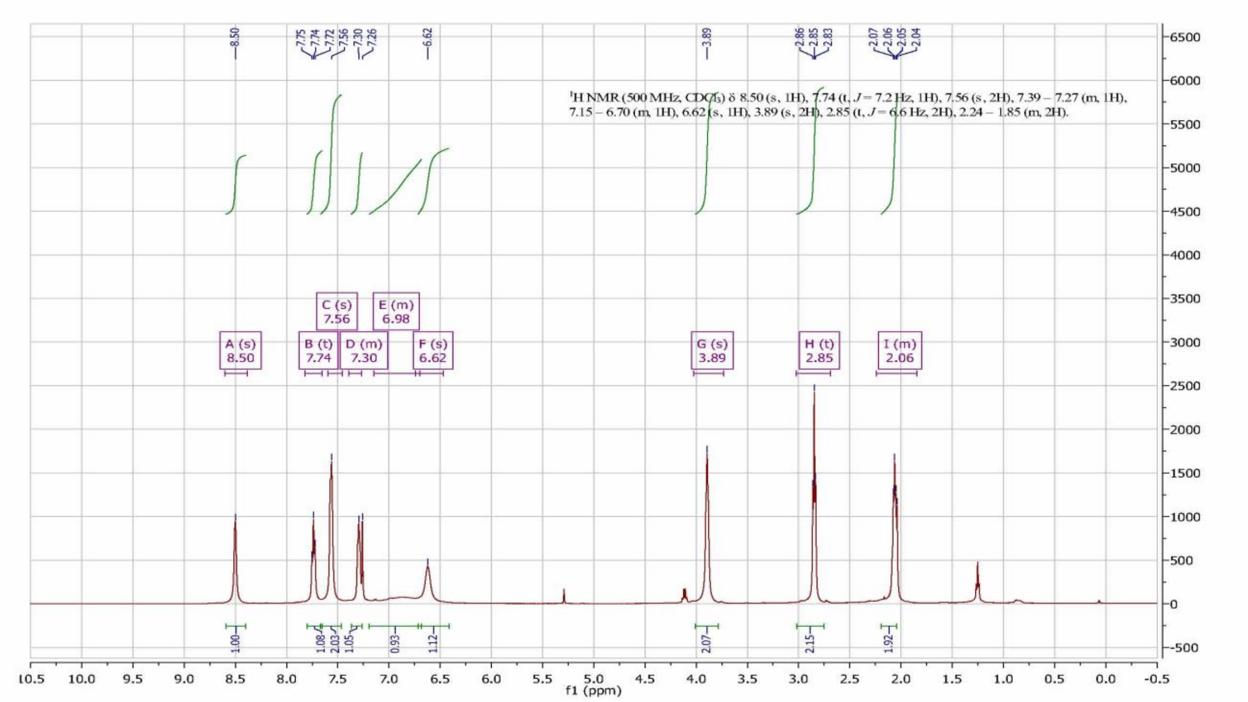
1H NMR (500 MHz, CDCl3) δ 8.50 (s, 1H), 7.74 (t, J = 7.2 Hz, 1H), 7.56 (s, 2H), 7.39 – 7.27 (m, 1H), 7.15 – 6.70 (m, 1H), 6.62 (s, 1H), 3.89 (s, 2H), 2.85 (t, J = 6.6 Hz, 2H), 2.24 – 1.85 (m, 2H).
f1 (ppm)

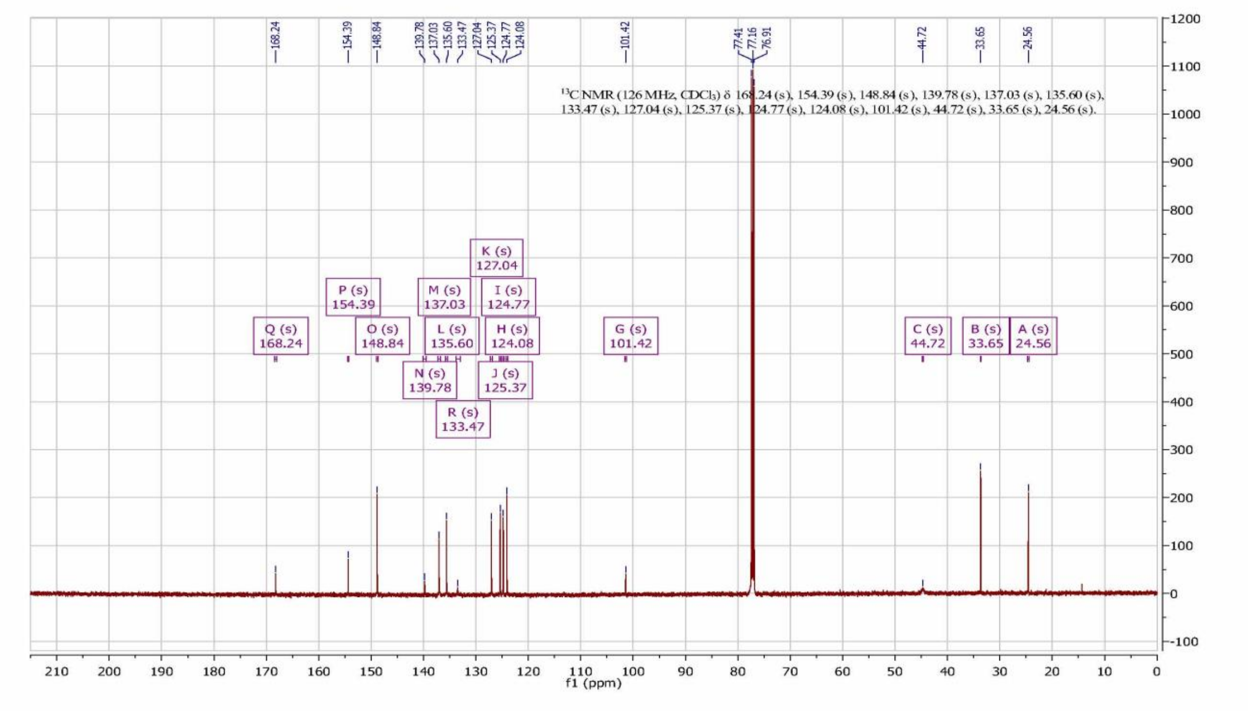
13C NMR (126 MHz, CDCl3) δ 168.24 (s), 154.39 (s), 148.84 (s), 139.78 (s), 137.03 (s), 135.60 (s), 133.47 (s), 127.04 (s), 125.37 (s), 124.77 (s), 124.08 (s), 101.42 (s), 44.72 (s), 33.65 (s), 24.56 (s).
f1 (ppm)

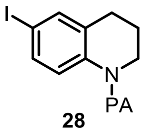
I
N
PA

28

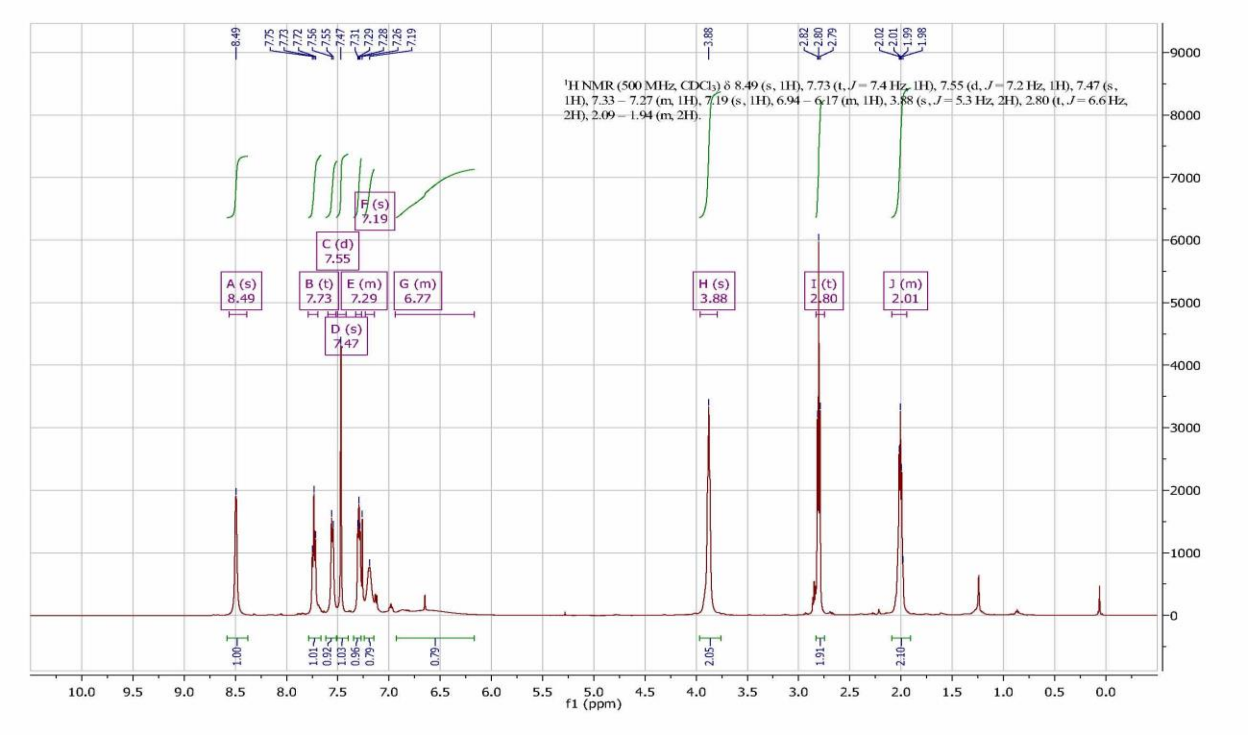
^{1}H NMR (500 MHz, $CDCl_3$) δ 8.49 (s, 1H), 7.73 (t, J = 7.4 Hz, 1H), 7.55 (d, J = 7.2 Hz, 1H), 7.47 (s, 1H), 7.33 – 7.27 (m, 1H), 7.19 (s, 1H), 6.94 – 6.17 (m, 1H), 3.88 (s, J = 5.3 Hz, 2H), 2.80 (t, J = 6.6 Hz, 2H), 2.09 – 1.94 (m, 2H).
A (s) 8.49
B (t) 7.73
C (d) 7.55
D (s) 7.47
E (m) 7.29
F (s) 7.19
G (m) 6.77
H (s) 3.88
I (t) 2.80
J (m) 2.01
f1 (ppm)

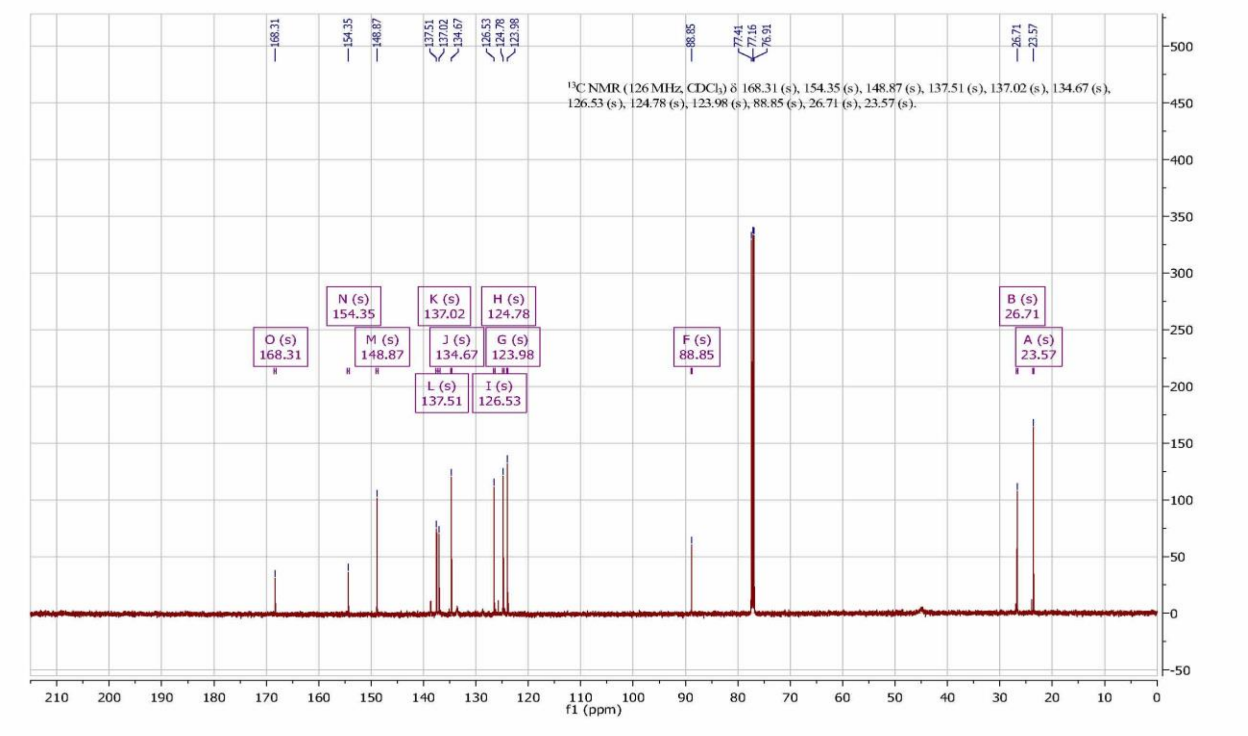
^{13}C NMR (126 MHz, $CDCl_3$) δ 168.31 (s), 154.35 (s), 148.87 (s), 137.51 (s), 137.02 (s), 134.67 (s), 126.53 (s), 124.78 (s), 123.98 (s), 88.85 (s), 26.71 (s), 23.57 (s).
O (s) 168.31
N (s) 154.35
M (s) 148.87
L (s) 137.51
K (s) 137.02
J (s) 134.67
I (s) 126.53
H (s) 124.78
G (s) 123.98
F (s) 88.85
B (s) 26.71
A (s) 23.57
f1 (ppm)

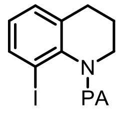
N
I
PA

29

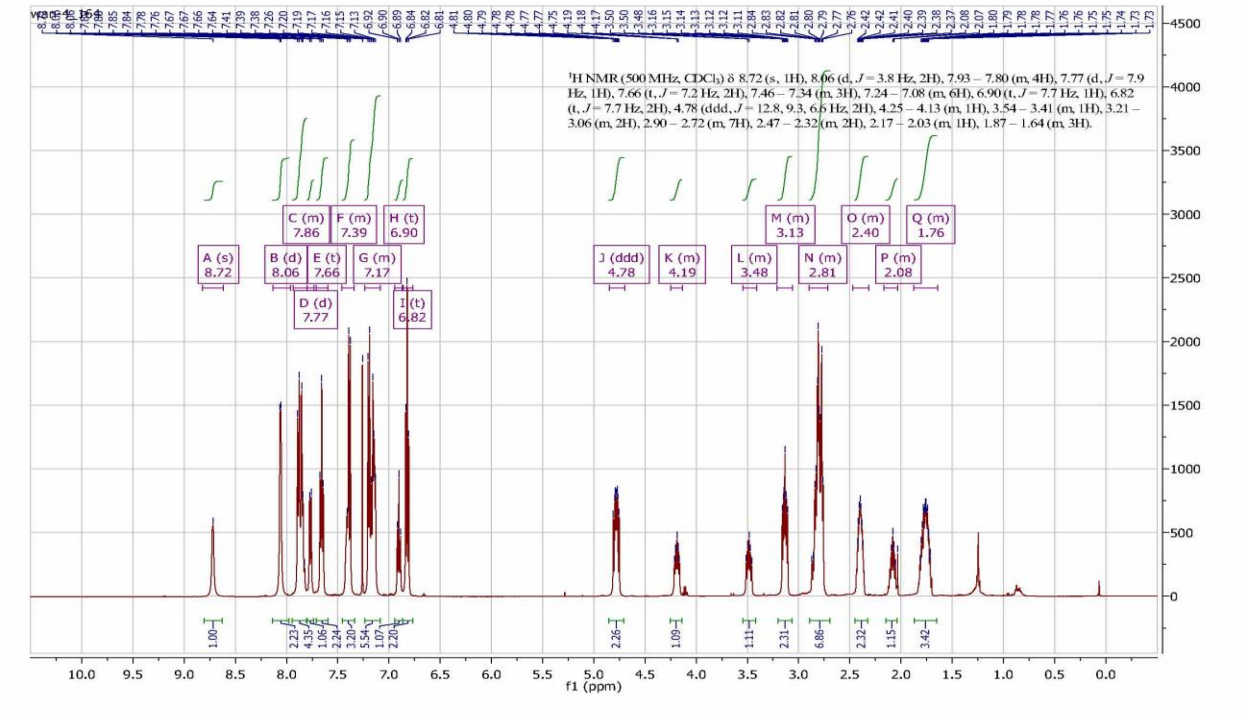
^{1}H NMR (500 MHz, CDCl$_3$) δ 8.72 (s, 1H), 8.06 (d, J = 3.8 Hz, 2H), 7.93 – 7.80 (m, 4H), 7.77 (d, J = 7.9 Hz, 1H), 7.66 (t, J = 7.2 Hz, 2H), 7.46 – 7.34 (m, 3H), 7.24 – 7.08 (m, 6H), 6.90 (t, J = 7.7 Hz, 1H), 6.82 (t, J = 7.7 Hz, 2H), 4.78 (ddd, J = 12.8, 9.3, 6.6 Hz, 2H), 4.25 – 4.13 (m, 1H), 3.54 – 3.41 (m, 1H), 3.21 – 3.06 (m, 2H), 2.90 – 2.72 (m, 7H), 2.47 – 2.32 (m, 2H), 2.17 – 2.03 (m, 1H), 1.87 – 1.64 (m, 3H).
f1 (ppm)

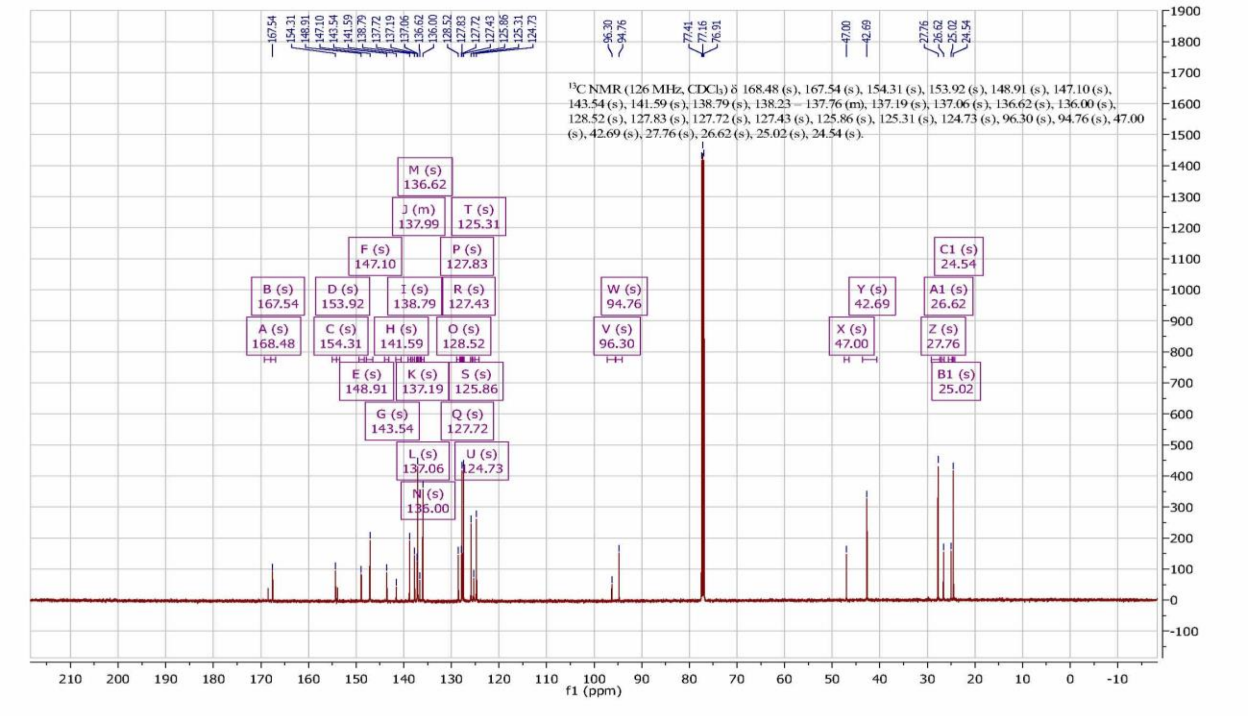
^{13}C NMR (126 MHz, CDCl$_3$) δ 168.48 (s), 167.54 (s), 154.31 (s), 153.92 (s), 148.91 (s), 147.10 (s), 143.54 (s), 141.59 (s), 138.79 (s), 138.23 – 137.76 (m), 137.19 (s), 137.06 (s), 136.62 (s), 136.00 (s), 128.52 (s), 127.83 (s), 127.72 (s), 127.43 (s), 125.86 (s), 125.31 (s), 124.73 (s), 96.30 (s), 94.76 (s), 47.00 (s), 42.69 (s), 27.76 (s), 26.62 (s), 25.02 (s), 24.54 (s).
f1 (ppm)

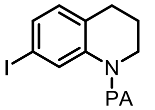
I
N
PA

30

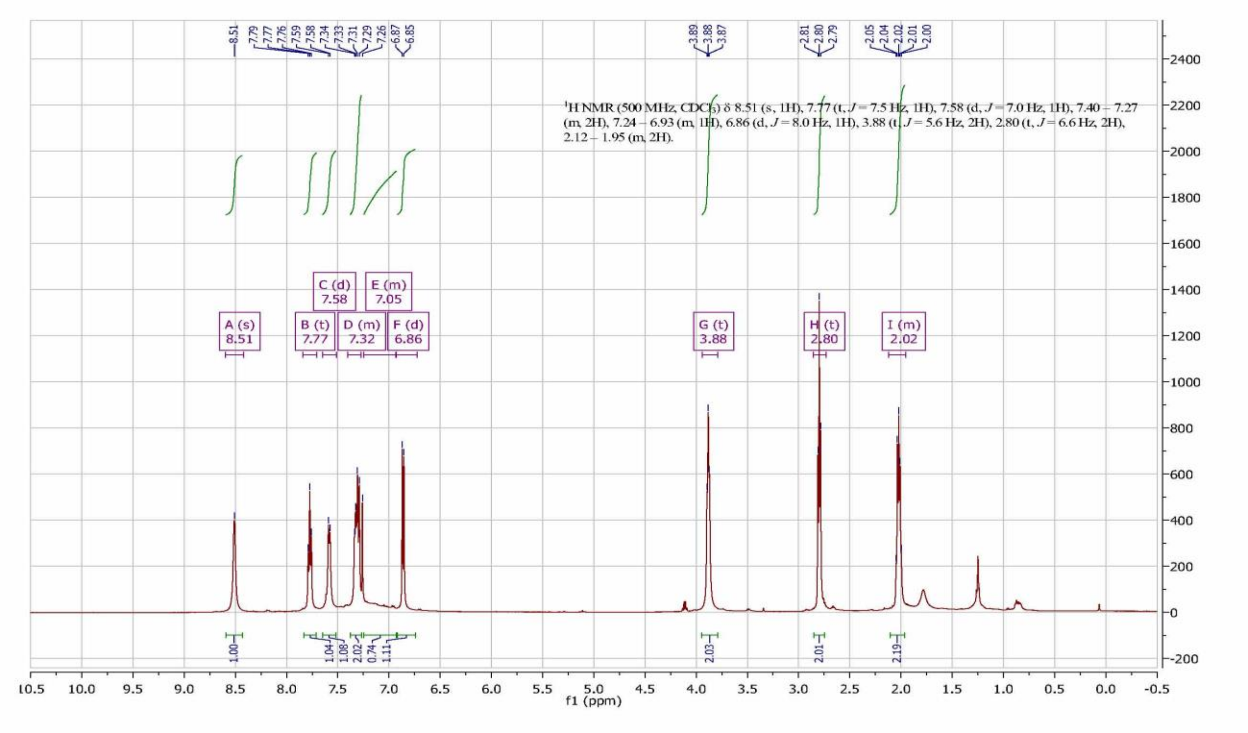
^{1}H NMR (500 MHz, $CDCl_3$) δ 8.51 (s, 1H), 7.77 (t, J = 7.5 Hz, 1H), 7.58 (d, J = 7.0 Hz, 1H), 7.40 – 7.27 (m, 2H), 7.24 – 6.93 (m, 1H), 6.86 (d, J = 8.0 Hz, 1H), 3.88 (t, J = 5.6 Hz, 2H), 2.80 (t, J = 6.6 Hz, 2H), 2.12 – 1.95 (m, 2H).
A (s) 8.51
B (t) 7.77
C (d) 7.58
D (m) 7.32
E (m) 7.05
F (d) 6.86
G (t) 3.88
H (t) 2.80
I (m) 2.02
f1 (ppm)

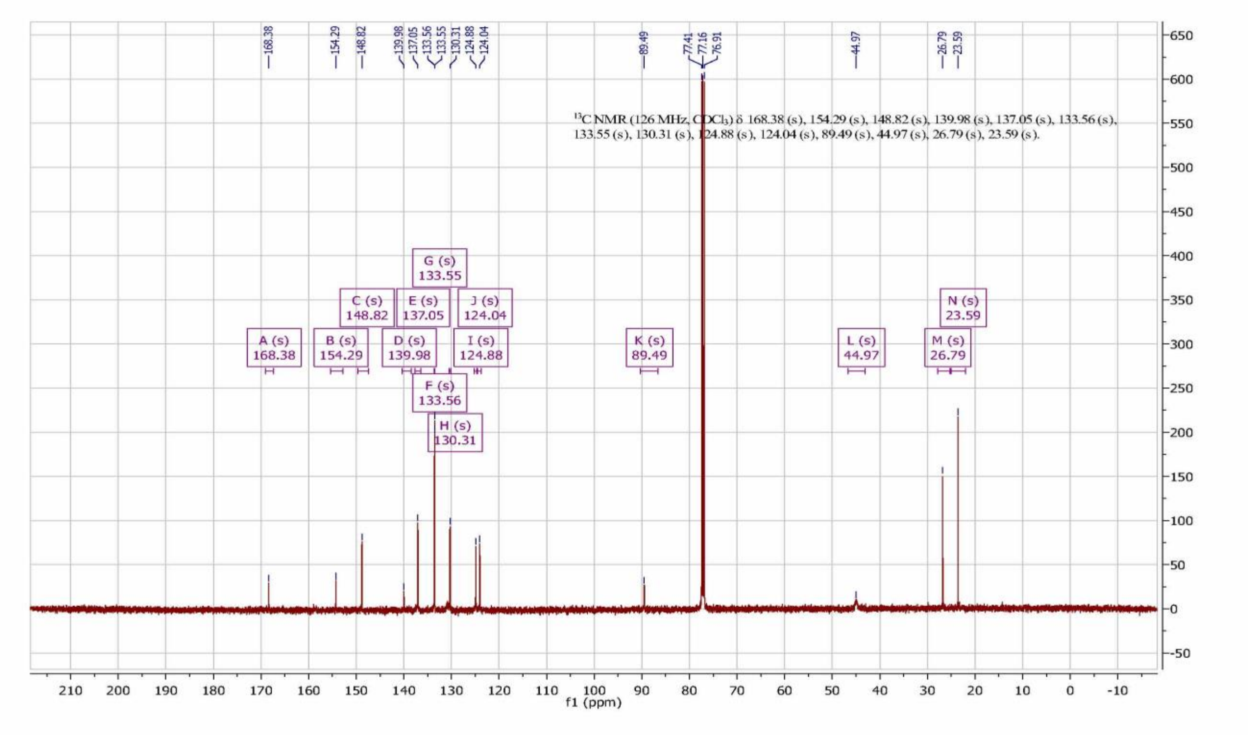
^{13}C NMR (126 MHz, $CDCl_3$) δ 168.38 (s), 154.29 (s), 148.82 (s), 139.98 (s), 137.05 (s), 133.56 (s), 133.55 (s), 130.31 (s), 124.88 (s), 124.04 (s), 89.49 (s), 44.97 (s), 26.79 (s), 23.59 (s).
A (s) 168.38
B (s) 154.29
C (s) 148.82
D (s) 139.98
E (s) 137.05
F (s) 133.56
G (s) 133.55
H (s) 130.31
I (s) 124.88
J (s) 124.04
K (s) 89.49
L (s) 44.97
M (s) 26.79
N (s) 23.59
f1 (ppm)

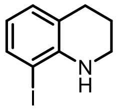
N
H
I

31

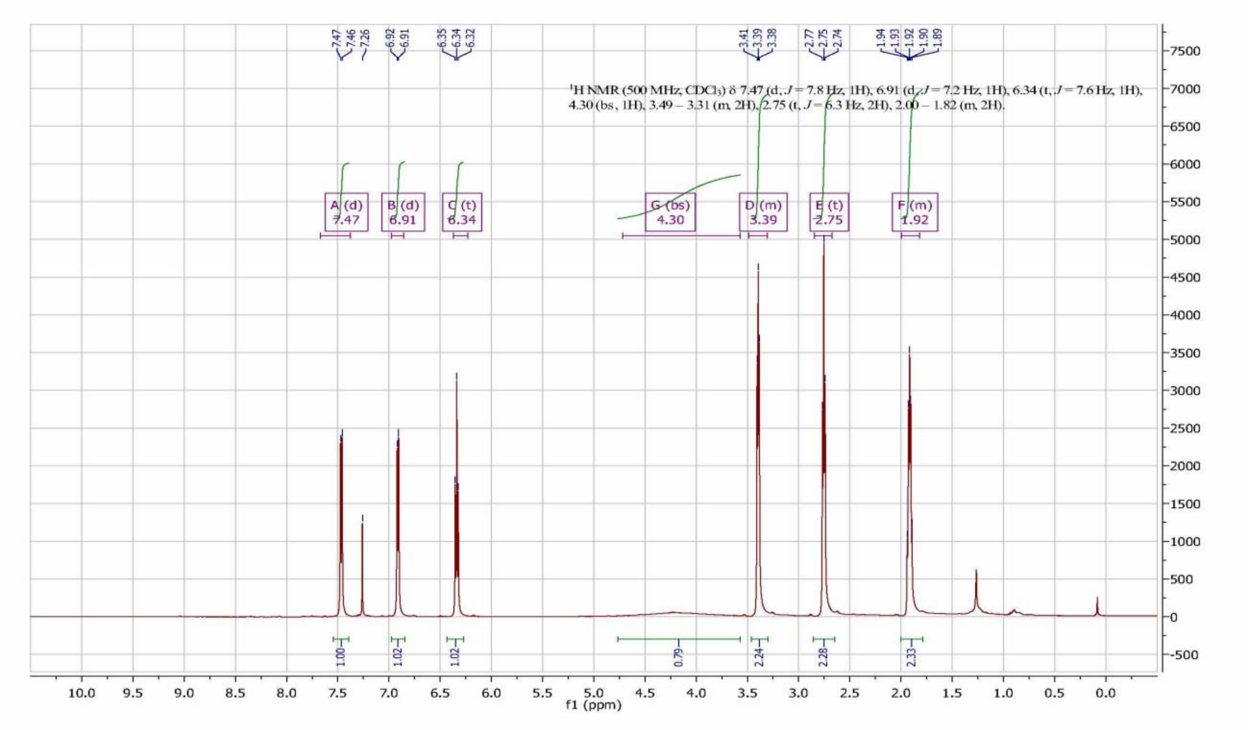
7.47
7.46
7.26
6.92
6.91
6.35
6.34
6.32
3.41
3.39
3.38
2.77
2.75
2.74
1.94
1.93
1.92
1.90
1.89
^{1}H NMR (500 MHz, $CDCl_3$) δ 7.47 (d, J = 7.8 Hz, 1H), 6.91 (d, J = 7.2 Hz, 1H), 6.34 (t, J = 7.6 Hz, 1H), 4.30 (bs , 1H), 3.49 – 3.31 (m, 2H), 2.75 (t, J = 6.3 Hz, 2H), 2.00 – 1.82 (m, 2H).
A (d) 7.47
B (d) 6.91
C (t) 6.34
G (bs) 4.30
D (m) 3.39
E (t) 2.75
F (m) 1.92
1.00
1.02
1.02
0.79
2.24
2.28
2.33
f1 (ppm)

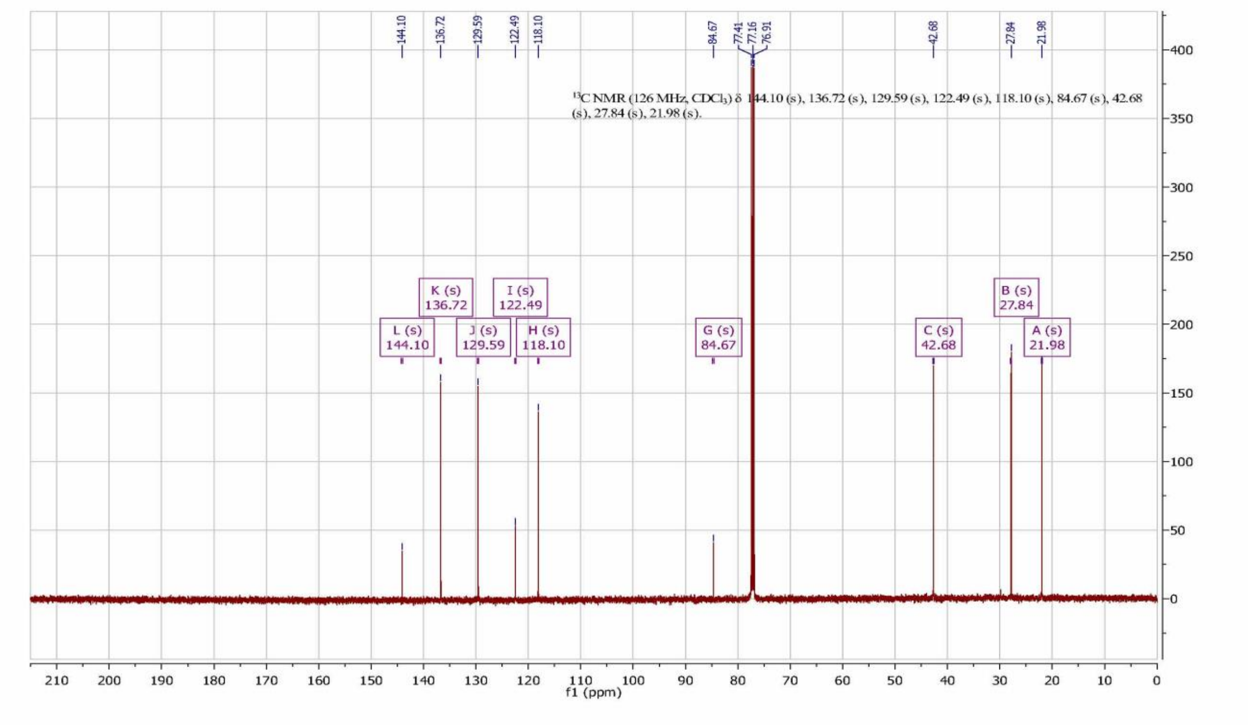
144.10
136.72
129.59
122.49
118.10
84.67
77.41
77.16
76.91
42.68
27.84
21.98
^{13}C NMR (126 MHz, $CDCl_3$) δ 144.10 (s), 136.72 (s), 129.59 (s), 122.49 (s), 118.10 (s), 84.67 (s), 42.68 (s), 27.84 (s), 21.98 (s).
K (s) 136.72
I (s) 122.49
L (s) 144.10
J (s) 129.59
H (s) 118.10
G (s) 84.67
C (s) 42.68
B (s) 27.84
A (s) 21.98
f1 (ppm)
